# Supplementary material for: Prioritization of risk genes for Alzheimer’s disease: an analysis framework using spatial and temporal gene expression data in the human brain based on support vector machine
Source: Front Genet. 2023 Oct 6;14:1190863. doi: 10.3389/fgene.2023.1190863 (PMC10587557; doi:10.3389/fgene.2023.1190863)
Supplement: Supplementary file 2 [file Table7.DOCX]

**Supplementary Table S7.** Comparative studies with their AUC.

| Research | Method | AUC |
| --- | --- | --- |
| Wang et al. 2022^[1]^ | GO and KEGG pathway enrichment analysis and PPI | 0.643 |
| Luo et al. 2017^[2]^ | dgSeq (including PPI) | 0.800 |
| Pei et al. 2023^[3]^ | Limma, PPI, functional enrichment analysis and WGCNA | 0.806 |
| Lagisetty et al. 2022^[4]^ | GeneEMBED | 0.63-0.84 |

And the detailed information of the studies in the table is listed as follows：

1. Wang Y, Chen G, Shao W. Identification of Ferroptosis-Related Genes in Alzheimer's Disease Based on Bioinformatic Analysis. Front Neurosci. 2022 Feb 7;16:823741. doi: 10.3389/fnins.2022.823741.
2. Luo P, Tian LP, Ruan J, Wu FX. Disease Gene Prediction by Integrating PPI Networks, Clinical RNA-Seq Data and OMIM Data. IEEE/ACM Trans Comput Biol Bioinform. 2019 Jan-Feb;16(1):222-232. doi: 10.1109/TCBB.2017.2770120. Epub 2017 Nov 7.
3. Pei Y, Chen S, Zhou F, Xie T, Cao H. Construction and evaluation of Alzheimer's disease diagnostic prediction model based on genes involved in mitophagy. Front Aging Neurosci. 2023 Mar 23;15:1146660. doi: 10.3389/fnagi.2023.1146660.
4. Lagisetty Y, Bourquard T, Al-Ramahi I, Mangleburg CG, Mota S, Soleimani S, Shulman JM, Botas J, Lee K, Lichtarge O. Identification of risk genes for Alzheimer's disease by gene embedding. Cell Genom. 2022 Sep 14;2(9):100162. doi: 10.1016/j.xgen.2022.100162.
