# Supplementary figures and images for: Prioritization of risk genes for Alzheimer’s disease: an analysis framework using spatial and temporal gene expression data in the human brain based on support vector machine
Source: Front Genet. 2023 Oct 6;14:1190863. doi: 10.3389/fgene.2023.1190863 (PMC10587557; doi:10.3389/fgene.2023.1190863)

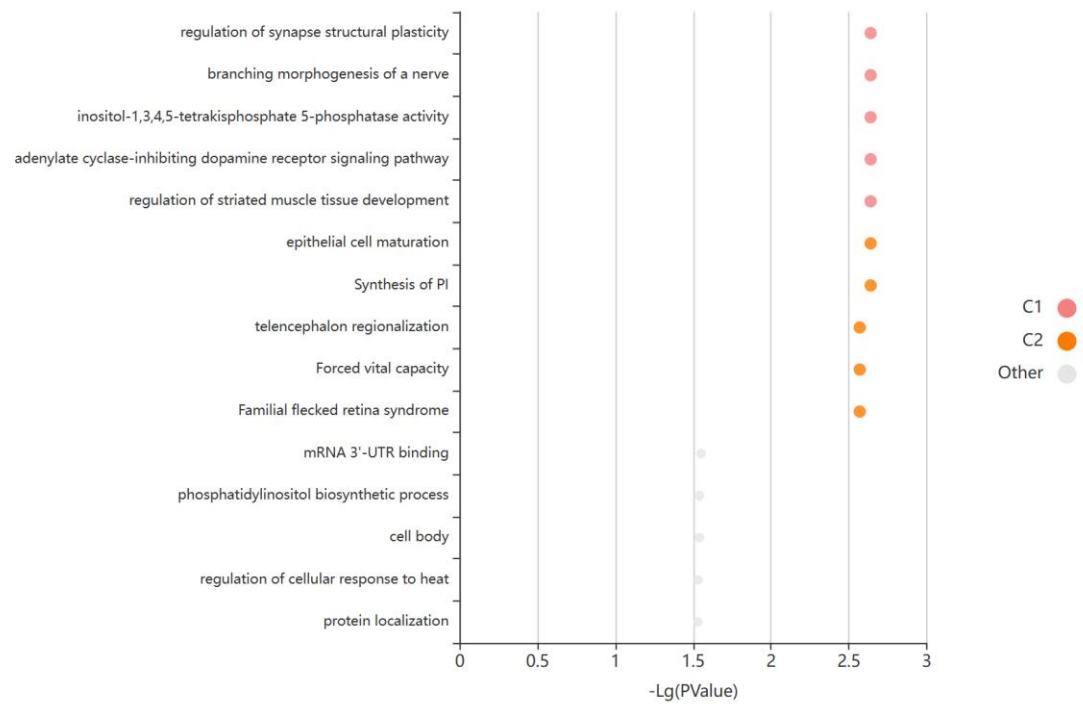

**Supplementary Figure S4.** Enriched terms visualized in bubble plot.

Supplement: Supplementary file 3 [file Image4.PDF]

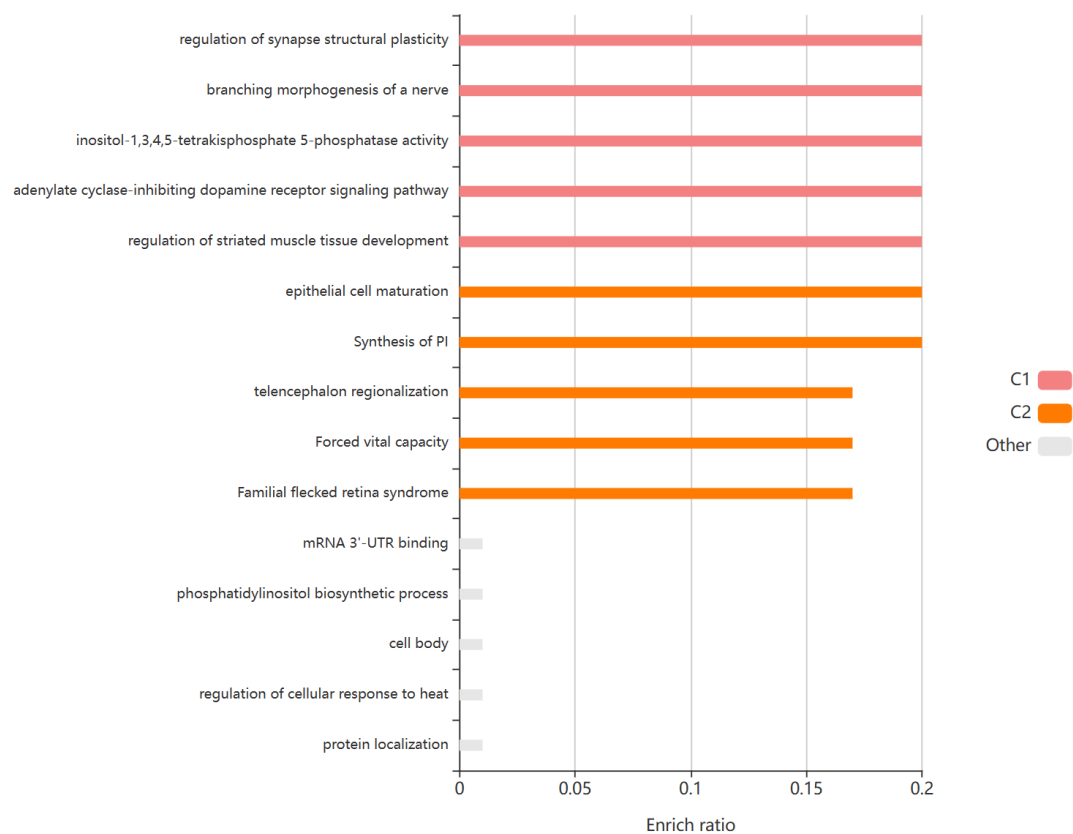

**Supplementary Figure S3.** Enriched terms visualized in barplot.

Supplement: Supplementary file 5 [file Image3.PDF]

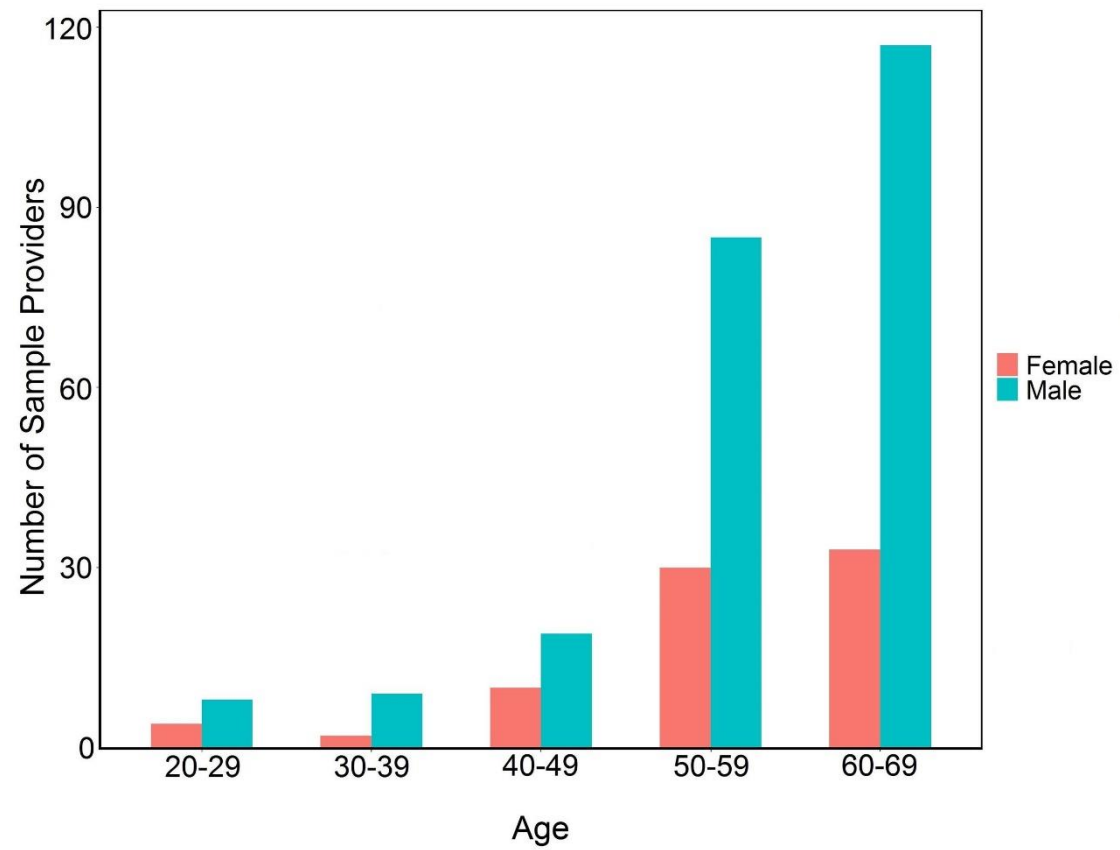

**Supplementary Figure S1.** Sex and age distribution of the tissue sample providers.

Supplement: Supplementary file 11 [file Image1.PDF]
