# Supplementary material for: Prioritization of risk genes for Alzheimer’s disease: an analysis framework using spatial and temporal gene expression data in the human brain based on support vector machine
Source: Front Genet. 2023 Oct 6;14:1190863. doi: 10.3389/fgene.2023.1190863 (PMC10587557; doi:10.3389/fgene.2023.1190863)
Supplement: Supplementary file 6 [file Table2.DOCX]

**Supplementary Table S2.** 3899 genes' CFG scores and GWAS traits.

| Gene | CFG | traits_name |
| --- | --- | --- |
| *KIF1B* | 4 | Intelligence |
| *FBLIM1* | 4 | Intelligence |
| *CDC42* | 4 | Intelligence |
| *WNT4* | 4 | Intelligence |
| *CNR2* | 4 | Intelligence |
| *LAPTM5* | 4 | Intelligence |
| *HCRTR1* | 4 | Intelligence |
| *LCK* | 4 | Intelligence |
| *EPS15* | 5 | Intelligence |
| *PCSK9* | 4 | Intelligence |
| *PRKAA2* | 5 | Intelligence |
| *JUN* | 4 | Intelligence |
| *GADD45A* | 4 | Peak expiratory flow |
| *NEGR1* | 4 | Peak expiratory flow |
| *CRYZ* | 4 | Peak expiratory flow |
| *PRKACB* | 4 | Peak expiratory flow |
| *CDC7* | 4 | Trauma exposure |
| *TGFBR3* | 4 | Trauma exposure |
| *VCAM1* | 4 | Trauma exposure |
| *VAV3* | 4 | Trauma exposure |
| *OTUD7B* | 4 | Trauma exposure |
| *MCL1* | 4 | Trauma exposure |
| *FCER1G* | 4 | Trauma exposure |
| *PBX1* | 4 | Trauma exposure |
| *RXRG* | 5 | Trauma exposure |
| *RGS2* | 4 | Trauma exposure |
| *NFASC* | 4 | Trauma exposure |
| *RASSF5* | 4 | Trauma exposure |
| *MAPKAPK2* | 4 | Trauma exposure |
| *MARK1* | 4 | Trauma exposure |
| *RHOU* | 4 | Trauma exposure |
| *AGT* | 4 | Trauma exposure |
| *GNG4* | 4 | Trauma exposure |
| *CHRM3* | 4 | Trauma exposure |
| *MYT1L* | 4 | Waist-to-hip ratio adjusted for BMI |
| *NTSR2* | 4 | Waist-to-hip ratio adjusted for BMI |
| *RHOB* | 4 | Waist-to-hip ratio adjusted for BMI |
| *DPYSL5* | 4 | Waist-to-hip ratio adjusted for BMI |
| *NRXN1* | 4 | Waist-to-hip ratio adjusted for BMI |
| *RTN4* | 4 | Waist-to-hip ratio adjusted for BMI |
| *TMEM17* | 4 | Waist-to-hip ratio adjusted for BMI |
| *CD8B* | 4 | Waist-to-hip ratio adjusted for BMI |
| *FHL2* | 4 | Hematocrit |
| *NCK2* | 4 | Hematocrit |
| *DBI* | 4 | Hematocrit |
| *GPR17* | 4 | Hematocrit |
| *HS6ST1* | 4 | Hematocrit |
| *KCNJ3* | 4 | Hematocrit |
| *EPHA4* | 4 | Hematocrit |
| *DNER* | 5 | Hematocrit |
| *INPP5D* | 4 | Hematocrit |
| *MLPH* | 4 | Hematocrit |
| *ARL8B* | 4 | Hematocrit |
| *IRAK2* | 4 | Hematocrit |
| *RARB* | 4 | Hematocrit |
| *RBMS3* | 4 | Hematocrit |
| *TGFBR2* | 4 | Hematocrit |
| *CMTM7* | 4 | Hematocrit |
| *CCR5* | 4 | Hematocrit |
| *CADPS* | 4 | Hematocrit |
| *LRIG1* | 4 | Hematocrit |
| *PROK2* | 4 | Hematocrit |
| *EPHA3* | 4 | Eosinophil counts |
| *IGSF11* | 4 | Eosinophil counts |
| *ITGB5* | 4 | Eosinophil counts |
| *MBNL1* | 4 | Eosinophil counts |
| *P2RY1* | 4 | Eosinophil counts |
| *MME* | 5 | Eosinophil counts |
| *SERPINI1* | 4 | Eosinophil counts |
| *EHHADH* | 4 | Eosinophil counts |
| *CRMP1* | 4 | Adolescent idiopathic scoliosis |
| *SLIT2* | 5 | Adolescent idiopathic scoliosis |
| *APBB2* | 4 | Adolescent idiopathic scoliosis |
| *IGFBP7* | 4 | Adolescent idiopathic scoliosis |
| *CXCL13* | 4 | Gut microbiota (bacterial taxa |
| *MAPK10* | 5 | hurdle binary method) |
| *GRID2* | 4 | Gut microbiota (bacterial taxa |
| *DAPP1* | 4 | Gut microbiota (bacterial taxa |
| *CASP6* | 4 | Gut microbiota (bacterial taxa |
| *CAMK2D* | 4 | Gut microbiota (bacterial taxa |
| *MAD2L1* | 4 | Alzheimer's disease |
| *TRPC3* | 4 | Gut microbiota (bacterial taxa |
| *ANKRD50* | 4 | hurdle binary method) |
| *IL15* | 4 | Gut microbiota (bacterial taxa |
| *GAB1* | 4 | Gut microbiota (bacterial taxa |
| *SMAD1* | 4 | hurdle binary method) |
| *EDNRA* | 5 | hurdle binary method) |
| *PLRG1* | 4 | Gut microbiota (bacterial taxa |
| *VEGFC* | 4 | Gut microbiota (bacterial taxa |
| *ADAMTS16* | 4 | hurdle binary method) |
| *CDH12* | 4 | Gut microbiota (bacterial taxa |
| *CDH6* | 5 | Waist-to-hip ratio adjusted for BMI |
| *SLC1A3* | 4 | Waist-to-hip ratio adjusted for BMI |
| *HTR1A* | 4 | Waist-to-hip ratio adjusted for BMI |
| *MEF2C* | 4 | Waist-to-hip ratio adjusted for BMI |
| *EFNA5* | 4 | Waist-to-hip ratio adjusted for BMI |
| *FEM1C* | 4 | Waist-to-hip ratio adjusted for BMI |
| *CDO1* | 4 | Waist-to-hip ratio adjusted for BMI |
| *MEGF10* | 4 | Waist-to-hip ratio adjusted for BMI |
| *KIF3A* | 4 | Waist-to-hip ratio adjusted for BMI |
| *ETF1* | 4 | Waist-to-hip ratio adjusted for BMI |
| *CTNNA1* | 4 | Waist-to-hip ratio adjusted for BMI |
| *CD14* | 4 | Waist-to-hip ratio adjusted for BMI |
| *DPYSL3* | 4 | Rate of cognitive decline in Alzheimer's disease |
| *ANXA6* | 4 | Rate of cognitive decline in Alzheimer's disease |
| *GALNT10* | 4 | Rate of cognitive decline in Alzheimer's disease |
| *GABRB2* | 4 | Rate of cognitive decline in Alzheimer's disease |
| *GABRG2* | 4 | Rate of cognitive decline in Alzheimer's disease |
| *CCNG1* | 4 | Rate of cognitive decline in Alzheimer's disease |
| *COL23A1* | 4 | Rate of cognitive decline in Alzheimer's disease |
| *F13A1* | 4 | Rate of cognitive decline in Alzheimer's disease |
| *NEDD9* | 4 | Rate of cognitive decline in Alzheimer's disease |
| *GPLD1* | 4 | Rate of cognitive decline in Alzheimer's disease |
| *HIST1H2BN* | 4 | Rate of cognitive decline in Alzheimer's disease |
| *NOTCH4* | 4 | Rate of cognitive decline in Alzheimer's disease |
| *HMGA1* | 4 | Rate of cognitive decline in Alzheimer's disease |
| *MAPK14* | 4 | Attention deficit hyperactivity disorder (hyperactivity-impulsivity symptoms) |
| *CCND3* | 4 | Attention deficit hyperactivity disorder (inattention symptoms) |
| *VEGFA* | 4 | Attention deficit hyperactivity disorder (inattention symptoms) |
| *CDC5L* | 4 | Attention deficit hyperactivity disorder (inattention symptoms) |
| *ELOVL5* | 4 | Attention deficit hyperactivity disorder (inattention symptoms) |
| *RIMS1* | 4 | Attention deficit hyperactivity disorder (inattention symptoms) |
| *MYO6* | 4 | Attention deficit hyperactivity disorder (inattention symptoms) |
| *AIM1* | 4 | Eosinophil counts |
| *HDAC2* | 4 | Eosinophil counts |
| *GJA1* | 5 | Eosinophil counts |
| *AKAP7* | 4 | Eosinophil counts |
| *RPS12* | 4 | Alzheimer's disease |
| *IFNGR1* | 4 | Eosinophil counts |
| *CITED2* | 4 | Rate of cognitive decline in Alzheimer's disease |
| *NMBR* | 4 | Rate of cognitive decline in Alzheimer's disease |
| *SYNJ2* | 4 | Rate of cognitive decline in Alzheimer's disease |
| *RPS6KA2* | 4 | Attention deficit hyperactivity disorder (inattention symptoms) |
| *THBS2* | 4 | Attention deficit hyperactivity disorder (inattention symptoms) |
| *DLL1* | 4 | Attention deficit hyperactivity disorder (inattention symptoms) |
| *PRKAR1B* | 4 | Attention deficit hyperactivity disorder |
| *MAD1L1* | 4 | Type 2 diabetes |
| *GNA12* | 4 | Type 2 diabetes |
| *ACTB* | 5 | Type 2 diabetes |
| *ETV1* | 4 | Type 2 diabetes |
| *HDAC9* | 4 | Type 2 diabetes |
| *NPY* | 4 | Type 2 diabetes |
| *SEC61G* | 4 | Type 2 diabetes |
| *CCT6A* | 4 | Type 2 diabetes |
| *SEMA3A* | 4 | Type 2 diabetes |
| *DMTF1* | 4 | Type 2 diabetes |
| *ADAM22* | 4 | Type 2 diabetes |
| *AKAP9* | 4 | Type 2 diabetes |
| *TAC1* | 4 | Type 2 diabetes |
| *AP1S1* | 4 | Type 2 diabetes |
| *RELN* | 4 | Type 2 diabetes |
| *CAV1* | 5 | Type 2 diabetes |
| *GRM8* | 5 | Type 2 diabetes |
| *IRF5* | 4 | Type 2 diabetes |
| *CHRM2* | 4 | Type 2 diabetes |
| *ZYX* | 4 | Type 2 diabetes |
| *CUL1* | 5 | Eosinophil percentage of white cells |
| *DLC1* | 4 | Eosinophil percentage of white cells |
| *NEFM* | 4 | Eosinophil percentage of white cells |
| *EPHX2* | 4 | Eosinophil percentage of white cells |
| *CLU* | 4 | Eosinophil percentage of white cells |
| *ELP3* | 4 | Hematocrit |
| *NRG1* | 5 | Hematocrit |
| *WWP1* | 4 | Hematocrit |
| *PLEKHF2* | 4 | Hematocrit |
| *SDC2* | 4 | Hematocrit |
| *PABPC1* | 4 | Hematocrit |
| *YWHAZ* | 4 | Hematocrit |
| *TRHR* | 4 | Hematocrit |
| *EBAG9* | 4 | Hematocrit |
| *ADCY8* | 4 | Hematocrit |
| *SLA* | 4 | Hematocrit |
| *SMARCA2* | 4 | Hematocrit |
| *SH3GL2* | 4 | Hematocrit |
| *B4GALT1* | 4 | Hematocrit |
| *APBA1* | 4 | Hematocrit |
| *RORB* | 4 | Hematocrit |
| *TLE1* | 4 | Hematocrit |
| *GADD45G* | 4 | Hematocrit |
| *ROR2* | 4 | Type 2 diabetes |
| *TMEM38B* | 4 | White matter microstructure (radial diusivities) |
| *SLC31A2* | 4 | White matter microstructure (radial diusivities) |
| *CDK5RAP2* | 4 | White matter microstructure (radial diusivities) |
| *STXBP1* | 4 | White matter microstructure (radial diusivities) |
| *ANAPC2* | 4 | White matter microstructure (radial diusivities) |
| *USP6NL* | 4 | White matter microstructure (radial diusivities) |
| *VIM* | 4 | White matter microstructure (radial diusivities) |
| *MSRB2* | 4 | White matter microstructure (radial diusivities) |
| *CREM* | 4 | White matter microstructure (radial diusivities) |
| *CXCL12* | 4 | White matter microstructure (radial diusivities) |
| *CAMK2G* | 4 | Major depressive disorder |
| *NRG3* | 4 | Major depressive disorder |
| *IFIT2* | 4 | Major depressive disorder |
| *SLC25A28* | 4 | Major depressive disorder |
| *SORCS3* | 5 | Major depressive disorder |
| *SORCS1* | 4 | Major depressive disorder |
| *ADRA2A* | 5 | Major depressive disorder |
| *FGFR2* | 4 | Major depressive disorder in trauma exposed individuals |
| *DOCK1* | 4 | Major depressive disorder in trauma exposed individuals |
| *IFITM2* | 4 | Trauma exposure |
| *IFITM3* | 4 | Trauma exposure |
| *TSPAN4* | 4 | Trauma exposure in major depressive disorder |
| *AP2A2* | 5 | Trauma exposure in major depressive disorder |
| *RHOG* | 4 | Trauma exposure in major depressive disorder |
| *PLEKHA7* | 4 | Trauma exposure in major depressive disorder |
| *RPS13* | 4 | Trauma exposure in major depressive disorder |
| *CAT* | 4 | Trauma exposure in major depressive disorder |
| *TRAF6* | 4 | Trauma exposure in major depressive disorder |
| *NR1H3* | 5 | Trauma exposure in major depressive disorder |
| *RAPSN* | 5 | Trauma exposure in major depressive disorder-negative individuals |
| *NDUFS3* | 4 | Trauma exposure in major depressive disorder-negative individuals |
| *STIP1* | 4 | Asthma |
| *CAPN1* | 4 | Asthma |
| *RELA* | 4 | Asthma |
| *ADRBK1* | 4 | Asthma |
| *CCND1* | 4 | Asthma |
| *FADD* | 4 | Asthma |
| *PAK1* | 4 | Asthma |
| *ARHGAP20* | 4 | Asthma |
| *BACE1* | 4 | Asthma |
| *ETS1* | 5 | Asthma |
| *CACNA1C* | 4 | Asthma |
| *FKBP4* | 4 | Asthma |
| *SCNN1A* | 4 | Peak expiratory flow |
| *HEBP1* | 4 | Peak expiratory flow |
| *CNTN1* | 4 | Peak expiratory flow |
| *ADAMTS20* | 4 | Peak expiratory flow |
| *TUBA1A* | 4 | Peak expiratory flow |
| *CSAD* | 4 | Peak expiratory flow |
| *RARG* | 4 | Eosinophil counts |
| *GRIP1* | 4 | Eosinophil counts |
| *RAP1B* | 4 | Eosinophil counts |
| *DCN* | 4 | Eosinophil counts |
| *UBE2N* | 4 | Eosinophil counts |
| *NEDD1* | 5 | Eosinophil counts |
| *IGF1* | 4 | Eosinophil counts |
| *RPH3A* | 4 | Eosinophil counts |
| *OAS2* | 4 | Eosinophil counts |
| *CAMKK2* | 5 | Postoperative myocardial infarction after cardiac surgery |
| *FLT1* | 5 | Postoperative stroke after cardiac surgery |
| *FOXO1* | 4 | Postoperative acute renal failure after cardiac surgery |
| *DLEU7* | 4 | Postoperative acute renal failure after cardiac surgery |
| *TNFSF13B* | 4 | Postoperative delirium after cardiac surgery |
| *CDC16* | 4 | Postoperative delirium after cardiac surgery |
| *TTC5* | 4 | Postoperative complication after cardiac surgery |
| *AKAP6* | 4 | Postoperative complication after cardiac surgery |
| *NFKBIA* | 4 | Insomnia |
| *FBXO33* | 4 | Insomnia |
| *PELI2* | 4 | Insomnia |
| *RTN1* | 4 | Insomnia |
| *SMOC1* | 4 | Insomnia |
| *RGS6* | 4 | Insomnia |
| *LTBP2* | 4 | Insomnia |
| *DLST* | 4 | Autoimmune thyroid disease |
| *SEL1L* | 4 | Autoimmune thyroid disease |
| *PTPN21* | 4 | Autoimmune thyroid disease |
| *EVL* | 4 | Autoimmune thyroid disease |
| *JAG2* | 4 | Autoimmune thyroid disease |
| *APBA2* | 4 | Autoimmune thyroid disease |
| *FSIP1* | 4 | Autoimmune thyroid disease |
| *PAK6* | 4 | Autoimmune thyroid disease |
| *FGF7* | 4 | Autoimmune thyroid disease |
| *ANXA2* | 4 | Autoimmune thyroid disease |
| *RORA* | 4 | Autoimmune thyroid disease |
| *RAB11A* | 4 | Autoimmune thyroid disease |
| *SMAD3* | 4 | Autoimmune thyroid disease |
| *MFGE8* | 4 | Eosinophil counts |
| *IQGAP1* | 4 | Eosinophil counts |
| *FES* | 4 | Eosinophil counts |
| *NR2F2* | 4 | Eosinophil counts |
| *MPG* | 4 | Eosinophil counts |
| *ABCA3* | 4 | Ease of getting up in the morning |
| *NDE1* | 4 | Ease of getting up in the morning |
| *GPRC5B* | 4 | Ease of getting up in the morning |
| *PRKCB* | 4 | Ease of getting up in the morning |
| *MAPK3* | 4 | Ease of getting up in the morning |
| *MMP2* | 4 | Ease of getting up in the morning |
| *CES3* | 4 | Ease of getting up in the morning |
| *BCAR1* | 4 | Ease of getting up in the morning |
| *PLCG2* | 4 | Ease of getting up in the morning |
| *CDH13* | 5 | Ease of getting up in the morning |
| *HSBP1* | 4 | Ease of getting up in the morning |
| *USP10* | 4 | Ease of getting up in the morning |
| *SERPINF2* | 4 | Ease of getting up in the morning |
| *CXCL16* | 4 | Ease of getting up in the morning |
| *RABEP1* | 4 | Ease of getting up in the morning |
| *NF1* | 4 | Ease of getting up in the morning |
| *STAT3* | 4 | Ease of getting up in the morning |
| *MAPT* | 5 | White matter microstructure (radial diusivities) |
| *GNGT2* | 4 | White matter microstructure (radial diusivities) |
| *BCAS3* | 4 | White matter microstructure (radial diusivities) |
| *RGS9* | 4 | Eosinophil percentage of white cells |
| *PRKCA* | 4 | Eosinophil percentage of white cells |
| *PITPNC1* | 4 | Hematocrit |
| *SLC9A3R1* | 4 | Hematocrit |
| *RAB12* | 4 | Hematocrit |
| *NAPG* | 4 | Hematocrit |
| *MIB1* | 4 | Hematocrit |
| *AQP4* | 4 | Hematocrit |
| *CDH7* | 4 | Hematocrit |
| *CBLN2* | 4 | Protein quantitative trait loci |
| *SALL3* | 4 | Protein quantitative trait loci |
| *NFATC1* | 4 | Protein quantitative trait loci |
| *PTPRS* | 4 | Protein quantitative trait loci |
| *TRPC3* | 4 | Protein quantitative trait loci |
| *VAV1* | 4 | Protein quantitative trait loci |
| *ELAVL1* | 4 | Protein quantitative trait loci |
| *CCL25* | 4 | Protein quantitative trait loci |
| *TYK2* | 4 | Protein quantitative trait loci |
| *IFI30* | 4 | Protein quantitative trait loci |
| *LSM4* | 4 | Protein quantitative trait loci |
| *NCAN* | 4 | Protein quantitative trait loci |
| *PPP1R14A* | 4 | Protein quantitative trait loci |
| *RYR1* | 4 | Protein quantitative trait loci |
| *BCL3* | 4 | Protein quantitative trait loci |
| *APOC1* | 4 | Protein quantitative trait loci |
| *PRKD2* | 4 | L-selectin levels |
| *RPS11* | 4 | L-selectin levels |
| *IRF3* | 4 | L-selectin levels |
| *PTPRA* | 4 | L-selectin levels |
| *SMOX* | 4 | L-selectin levels |
| *PROKR2* | 4 | Protein quantitative trait loci |
| *SNX5* | 4 | Protein quantitative trait loci |
| *BCL2L1* | 4 | Protein quantitative trait loci |
| *MAPRE1* | 4 | Nonalcoholic fatty liver disease |
| *PLCG1* | 4 | Type 2 diabetes |
| *SDC4* | 4 | Type 2 diabetes |
| *CD40* | 4 | Type 2 diabetes |
| *CTSZ* | 4 | Type 2 diabetes |
| *CDH4* | 5 | Type 2 diabetes |
| *DAPP1* | 4 | Type 2 diabetes |
| *DSCAM* | 4 | Type 2 diabetes |
| *MAPK10* | 5 | Type 2 diabetes |
| *HMOX1* | 4 | Type 2 diabetes |
| *ATF4* | 4 | Type 2 diabetes |
| *RANGAP1* | 4 | Type 2 diabetes |
| *PRKX* | 4 | Type 2 diabetes |
| *FIGF* | 4 | Type 2 diabetes |
| *MARK1* | 4 | Type 2 diabetes |
| *F9* | 4 | Type 2 diabetes |
| *NADK* | 2 | Type 2 diabetes |
| *GNB1* | 2 | Type 2 diabetes |
| *PRKCZ* | 3 | General cognitive ability |
| *No data avaiable about C1ORF86 in this dataset!* | NA | General cognitive ability |
| *No data avaiable about AL590822.1 in this dataset!* | NA | General cognitive ability |
| *No data avaiable about RP11-181G12.4 in this dataset!* | NA | General cognitive ability |
| *SKI* | 0 | General cognitive ability |
| *MORN1* | 2 | Eosinophil percentage of white cells |
| *RER1* | 1 | Eosinophil percentage of white cells |
| *PLCH2* | 1 | Eosinophil percentage of white cells |
| *PANK4* | 2 | Eosinophil percentage of white cells |
| *TNFRSF14* | 1 | Hematocrit |
| *FAM213B* | 0 | Hematocrit |
| *MMEL1* | 0 | Hematocrit |
| *TTC34* | 0 | Hematocrit |
| *ACTRT2* | 0 | Hematocrit |
| *LINC00982* | 0 | Hematocrit |
| *PRDM16* | 0 | Hematocrit |
| *HNRNPR* | 1 | Suicide attempts |
| *ZNF436* | 1 | Suicide attempts |
| *RASSF5* | 4 | Suicide attempts |
| *SELP* | 0 | Suicide attempts |
| *C1orf112* | NA | Suicide attempts |
| *SELL* | 0 | Interleukin-6 levels |
| *SELE* | 1 | Deliberate self-harm |
| *KIFAP3* | 1 | Deliberate self-harm |
| *METTL11B* | 2 | Deliberate self-harm |
| *No data avaiable about RP11-297H3.3 in this dataset!* | NA | Deliberate self-harm |
| *ENO1* | 0 | White matter microstructure (radial diusivities) |
| *ENO1-AS1* | 0 | White matter microstructure (radial diusivities) |
| *CA6* | 1 | White matter microstructure (radial diusivities) |
| *No data avaiable about RP3-510D11.2 in this dataset!* | NA | White matter microstructure (radial diusivities) |
| *SELENBP1* | 3 | White matter microstructure (radial diusivities) |
| *POGZ* | 1 | White matter microstructure (radial diusivities) |
| *OAZ3* | 1 | White matter microstructure (radial diusivities) |
| *TDRKH* | 3 | White matter microstructure (radial diusivities) |
| *LINGO4* | 1 | White matter microstructure (mode of anisotropy) |
| *RORC* | 2 | White matter microstructure (mode of anisotropy) |
| *THEM5* | 0 | Reaction time |
| *THEM4* | 2 | Reaction time |
| *S100A10* | 3 | Reaction time |
| *TCHHL1* | 0 | Reaction time |
| *TCHHL1* | 0 | Reaction time |
| *RPTN* | 0 | Major depressive disorder |
| *FLG-AS1* | 0 | Major depressive disorder |
| *No data avaiable about RP11-107M16.2 in this dataset!* | NA | Major depressive disorder |
| *FLG-AS1* | 0 | Major depressive disorder |
| *SRP9* | 2 | Major depressive disorder |
| *EPHX1* | 1 | Major depressive disorder |
| *H3F3A* | 0 | Major depressive disorder |
| *No data avaiable about RP11-396C23.3 in this dataset!* | NA | Major depressive disorder |
| *ACBD3* | 1 | Major depressive disorder |
| *LIN9* | 1 | Major depressive disorder |
| *C2orf16* | NA | Major depressive disorder |
| *ZNF512* | 1 | Major depressive disorder |
| *CCDC121* | 1 | Major depressive disorder |
| *GPN1* | 1 | Major depressive disorder |
| *SLC4A1AP* | 3 | Eosinophil counts |
| *MRPL33* | 2 | Eosinophil counts |
| *RBKS* | 1 | Eosinophil counts |
| *BRE* | 1 | Eosinophil counts |
| *FOSL2* | 1 | Eosinophil counts |
| *PLB1* | 1 | Eosinophil counts |
| *CAMKMT* | 0 | Eosinophil counts |
| *SIX3-AS1* | 0 | Eosinophil counts |
| *SIX3-AS1* | 0 | Eosinophil counts |
| *SRBD1* | 1 | Eosinophil counts |
| *PRKCE* | 2 | White matter microstructure (axial diusivities) |
| *EPAS1* | 3 | White matter microstructure (axial diusivities) |
| *No data avaiable about AC016912.3 in this dataset!* | NA | White matter microstructure (axial diusivities) |
| *ATP6V1E2* | 0 | White matter microstructure (axial diusivities) |
| *SOCS5* | 2 | White matter microstructure (axial diusivities) |
| *No data avaiable about AC016722.4 in this dataset!* | NA | White matter microstructure (axial diusivities) |
| *MCFD2* | 1 | White matter microstructure (axial diusivities) |
| *TTC7A* | 1 | White matter microstructure (axial diusivities) |
| *CALM2* | 1 | White matter microstructure (axial diusivities) |
| *BCYRN1* | 0 | White matter microstructure (axial diusivities) |
| *EPCAM* | 0 | White matter microstructure (radial diusivities) |
| *MSH2* | 2 | White matter microstructure (radial diusivities) |
| *TRABD2A* | 0 | White matter microstructure (radial diusivities) |
| *TCF7L1* | 0 | White matter microstructure (radial diusivities) |
| *No data avaiable about AC093162.5 in this dataset!* | NA | White matter microstructure (radial diusivities) |
| *TGOLN2* | 1 | White matter microstructure (radial diusivities) |
| *SLC4A10* | 1 | White matter microstructure (radial diusivities) |
| *AHCTF1P1* | 0 | Gut microbiota (bacterial taxa |
| *DPP4* | 2 | hurdle binary method) |
| *KIFAP3* | 1 | hurdle binary method) |
| *IFIH1* | 0 | Gut microbiota (bacterial taxa |
| *GCA* | 0 | hurdle binary method) |
| *KCNH7* | 1 | Gut microbiota (bacterial taxa |
| *FIGN* | 1 | Gut microbiota (bacterial taxa |
| *No data avaiable about AC092684.1 in this dataset!* | NA | Gut microbiota (bacterial taxa |
| *NAB1* | 0 | Gut microbiota (bacterial taxa |
| *GLS* | 0 | Urine 6-bromotryptophan levels in chronic kidney disease |
| *STAT1* | 2 | General cognitive ability |
| *STAT4* | 1 | General cognitive ability |
| *MYO1B* | 3 | General cognitive ability |
| *NABP1* | 0 | General cognitive ability |
| *SDPR* | 0 | General cognitive ability |
| *TMEFF2* | 2 | Hematocrit |
| *PCGEM1* | 0 | Hematocrit |
| *SMARCAL1* | 0 | Hematocrit |
| *IGFBP2* | 3 | Hematocrit |
| *IGFBP5* | 3 | Hematocrit |
| *No data avaiable about AC007563.5 in this dataset!* | NA | Hematocrit |
| *TNP1* | 1 | Hematocrit |
| *No data avaiable about AC007557.1 in this dataset!* | NA | Hematocrit |
| *No data avaiable about AC017028.1 in this dataset!* | NA | Eosinophil counts |
| *No data avaiable about AC079612.1 in this dataset!* | NA | Eosinophil counts |
| *NDUFA10* | 1 | Eosinophil counts |
| *OR6B2* | 0 | Eosinophil counts |
| *OR9S24P* | 0 | Eosinophil counts |
| *OTOS* | 2 | Eosinophil counts |
| *GPC1* | 3 | Gut microbiota (bacterial taxa |
| *ANKMY1* | 1 | hurdle binary method) |
| *DUSP28* | 0 | Gut microbiota (bacterial taxa |
| *RNPEPL1* | 1 | hurdle binary method) |
| *GPR35* | 1 | hurdle binary method) |
| *KIF1A* | 0 | hurdle binary method) |
| *AGXT* | 1 | Gut microbiota (bacterial taxa |
| *C2orf54* | NA | hurdle binary method) |
| *XYLB* | 1 | Gut microbiota (bacterial taxa |
| *ACVR2B* | 0 | Gut microbiota (bacterial taxa |
| *EXOG* | 0 | Gut microbiota (bacterial taxa |
| *SCN5A* | 1 | Gut microbiota (bacterial taxa |
| *SCN10A* | 0 | hurdle binary method) |
| *SCN11A* | 3 | hurdle binary method) |
| *WDR48* | 1 | Gut microbiota (bacterial taxa |
| *CSRNP1* | 0 | hurdle binary method) |
| *XIRP1* | 1 | Gut microbiota (bacterial taxa |
| *CX3CR1* | 2 | hurdle binary method) |
| *CCR8* | 2 | hurdle binary method) |
| *MOBP* | 2 | hurdle binary method) |
| *MYRIP* | 3 | Brain amyloid deposition (PET imaging) |
| *No data avaiable about AC097493.1 in this dataset!* | NA | Brain amyloid deposition (PET imaging) |
| *RPS23P3* | 0 | Brain amyloid deposition (PET imaging) |
| *RNU6-699P* | 0 | Brain amyloid deposition (PET imaging) |
| *No data avaiable about RP11-584P21.2 in this dataset!* | NA | Brain amyloid deposition (PET imaging) |
| *CENPC* | 1 | White matter microstructure (radial diusivities) |
| *TMPRSS11D* | 1 | White matter microstructure (radial diusivities) |
| *TMPRSS11F* | 1 | White matter microstructure (radial diusivities) |
| *YTHDC1* | 2 | White matter microstructure (radial diusivities) |
| *TMPRSS11E* | 0 | White matter microstructure (radial diusivities) |
| *UGT2B29P* | 0 | General cognitive ability |
| *UGT2B17* | 0 | General cognitive ability |
| *UGT2B15* | 0 | General cognitive ability |
| *No data avaiable about CTD-2005D20.1 in this dataset!* | NA | General cognitive ability |
| *No data avaiable about RP11-468N14.1 in this dataset!* | NA | General cognitive ability |
| *UGT2B10* | 0 | General cognitive ability |
| *PLA2G12A* | 2 | General cognitive ability |
| *CFI* | 1 | General cognitive ability |
| *VEGFC* | 4 | General cognitive ability |
| *ELOVL6* | 3 | General cognitive ability |
| *ENPEP* | 0 | General cognitive ability |
| *PITX2* | 2 | General cognitive ability |
| *No data avaiable about AC008834.1 in this dataset!* | NA | General cognitive ability |
| *RGMB* | 2 | General cognitive ability |
| *CHD1* | 0 | General cognitive ability |
| *No data avaiable about CTD-2007H13.3 in this dataset!* | NA | General cognitive ability |
| *No data avaiable about RP11-93O17.1 in this dataset!* | NA | General cognitive ability |
| *No data avaiable about CTD-2160D9.1 in this dataset!* | NA | Eosinophil percentage of white cells |
| *EEF1A1P20* | 0 | Eosinophil percentage of white cells |
| *FAM174A* | 1 | Eosinophil percentage of white cells |
| *ST8SIA4* | 2 | Eosinophil percentage of white cells |
| *RN7SL802P* | 0 | Eosinophil percentage of white cells |
| *OR7H2P* | 0 | Hematocrit |
| *PPARGC1B* | 2 | Hematocrit |
| *MIR378A* | 0 | Hematocrit |
| *SLC26A2* | 0 | Hematocrit |
| *TIGD6* | 1 | Hematocrit |
| *HMGXB3* | 0 | Hematocrit |
| *CSF1R* | 2 | Hematocrit |
| *PDGFRB* | 1 | Hematocrit |
| *CDX1* | 1 | Hematocrit |
| *CAMK2A* | 2 | Migraine and/or pulse pressure |
| *ARSI* | 1 | Migraine and/or pulse pressure |
| *CD74* | 0 | Migraine with aura and/or systolic blood pressure |
| *RPS14* | 3 | Migraine without aura and/or diastolic blood pressure |
| *NDST1* | 2 | Migraine without aura and/or systolic blood pressure |
| *TPMT* | 0 | Migraine without aura and/or pulse pressure |
| *KDM1B* | 1 | Migraine without aura and/or pulse pressure |
| *DCDC2* | 0 | Waist-to-hip ratio adjusted for BMI |
| *KIAA0319* | 1 | Waist-to-hip ratio adjusted for BMI |
| *C6orf62* | NA | Waist-to-hip ratio adjusted for BMI |
| *GMNN* | 2 | Waist-to-hip ratio adjusted for BMI |
| *FAM65B* | 1 | Waist-to-hip ratio adjusted for BMI |
| *KLHL31* | 0 | Waist-to-hip ratio adjusted for BMI |
| *LRRC1* | 1 | White matter microstructure (radial diusivities) |
| *MLIP* | 1 | White matter microstructure (radial diusivities) |
| *TINAG* | 1 | White matter microstructure (radial diusivities) |
| *KRASP1* | 0 | White matter microstructure (radial diusivities) |
| *FAM83B* | 1 | White matter microstructure (radial diusivities) |
| *HCRTR2* | 2 | White matter microstructure (radial diusivities) |
| *GFRAL* | 1 | White matter microstructure (radial diusivities) |
| *DGKB* | 1 | White matter microstructure (radial diusivities) |
| *No data avaiable about AC006458.3 in this dataset!* | NA | White matter microstructure (radial diusivities) |
| *AGMO* | 2 | White matter microstructure (radial diusivities) |
| *MEOX2* | 0 | Peginterferon alfa-2a treatment response in chronic hepatitis B infection |
| *ISPD* | 1 | Hemoglobin concentration |
| *NOD1* | 0 | Hemoglobin concentration |
| *CRHR2* | 2 | Hemoglobin concentration |
| *INMT* | 3 | Hemoglobin concentration |
| *FAM188B* | 1 | Parental lifespan |
| *GS1-124K5.11* | 0 | Parental lifespan |
| *KCTD7* | 1 | Parental lifespan |
| *RABGEF1* | 2 | Parental lifespan |
| *GTF2IRD1P1* | 0 | Parental lifespan |
| *TFEC* | 0 | Neuroticism |
| *TES* | 1 | Neuroticism |
| *CAV2* | 3 | Neuroticism |
| *METTL11B* | 2 | Neuroticism |
| *CAPZA2* | 0 | Neuroticism |
| *MCPH1* | 3 | Neuroticism |
| *ANGPT2* | 1 | Neuroticism |
| *AGPAT5* | 0 | Neuroticism |
| *XKR5* | 0 | Neuroticism |
| *GS1-24F4.2* | 0 | Neuroticism |
| *DEFB1* | 0 | Neuroticism |
| *ATP6V0D2* | 0 | Neuroticism |
| *SLC7A13* | 0 | Neuroticism |
| *RMDN1* | 0 | Caffeine consumption from coffee or tea |
| *CNGB3* | 0 | Caffeine consumption from coffee or tea |
| *CNBD1* | 0 | Caffeine consumption from coffee or tea |
| *No data avaiable about RP11-31K23.1 in this dataset!* | NA | Caffeine consumption from coffee or tea |
| *No data avaiable about RP11-44N17.2 in this dataset!* | NA | Caffeine consumption from coffee or tea |
| *PTDSS1* | 1 | Caffeine consumption from coffee or tea |
| *CPQ* | 1 | Caffeine consumption from coffee or tea |
| *TSPYL5* | 1 | Caffeine consumption from coffee or tea |
| *No data avaiable about KB-1683C8.1 in this dataset!* | NA | Waist-to-hip ratio adjusted for BMI |
| *MTDH* | 2 | Waist-to-hip ratio adjusted for BMI |
| *LAPTM4B* | 1 | Waist-to-hip ratio adjusted for BMI |
| *MIR4675* | 0 | Waist-to-hip ratio adjusted for BMI |
| *NEBL* | 1 | Waist-to-hip ratio adjusted for BMI |
| *C10orf113* | NA | Waist-to-hip ratio adjusted for BMI |
| *SKIDA1* | 0 | Waist-to-hip ratio adjusted for BMI |
| *MLLT10* | 2 | Waist-to-hip ratio adjusted for BMI |
| *DNAJC1* | 0 | Waist-to-hip ratio adjusted for BMI |
| *BMS1* | 2 | Caffeine consumption from coffee or tea |
| *No data avaiable about RP11-124O11.1 in this dataset!* | NA | Caffeine consumption from coffee or tea |
| *RET* | 2 | Caffeine consumption from coffee or tea |
| *CSGALNACT2* | 1 | Caffeine consumption from coffee or tea |
| *RASGEF1A* | 1 | Caffeine consumption from coffee or tea |
| *FXYD4* | 1 | Caffeine consumption from coffee or tea |
| *HNRNPF* | 1 | Major depressive disorder in trauma exposed individuals |
| *ZNF487* | 0 | Major depressive disorder in trauma exposed individuals |
| *No data avaiable about RP11-257I14.1 in this dataset!* | NA | Major depressive disorder in trauma exposed individuals |
| *MIR548F1* | 0 | Major depressive disorder in trauma-unexposed individuals |
| *MTRNR2L5* | 0 | Major depressive disorder in trauma-unexposed individuals |
| *ZWINT* | 3 | Major depressive disorder in trauma-unexposed individuals |
| *MRPS35P3* | 0 | Major depressive disorder in trauma-unexposed individuals |
| *IPMK* | 2 | Major depressive disorder in trauma-unexposed individuals |
| *UBE2D1* | 3 | Asthma |
| *DUSP13* | 1 | White matter microstructure (axial diusivities) |
| *No data avaiable about RP11-399K21.11 in this dataset!* | NA | White matter microstructure (axial diusivities) |
| *ZNF503* | 0 | Asthma |
| *No data avaiable about RP11-399K21.10 in this dataset!* | NA | Asthma |
| *C10orf113* | NA | Asthma |
| *CHST15* | 2 | Asthma |
| *LHPP* | 0 | Asthma |
| *FAM53B* | 0 | Asthma |
| *METTL10* | 0 | Blood protein levels |
| *FAM175B* | 1 | Blood protein levels |
| *No data avaiable about RP11-298J20.4 in this dataset!* | NA | Blood protein levels |
| *ZRANB1* | 1 | Blood protein levels |
| *CTBP2* | 1 | Blood protein levels |
| *RPS27P18* | 0 | Blood protein levels |
| *BET1L* | 1 | Hematocrit |
| *SCGB1C1* | 0 | Hematocrit |
| *ODF3* | 1 | Hematocrit |
| *RIC8A* | 1 | Hematocrit |
| *SIRT3* | 3 | Hematocrit |
| *PSMD13* | 2 | Hematocrit |
| *NLRP6* | 1 | Hematocrit |
| *ATHL1* | 0 | Eosinophil counts |
| *IFITM5* | 0 | Eosinophil counts |
| *OR8A1* | 0 | Eosinophil counts |
| *SIAE* | 1 | Trauma exposure in major depressive disorder-negative individuals |
| *NRGN* | 1 | Trauma exposure in major depressive disorder-negative individuals |
| *ESAM* | 1 | Trauma exposure in major depressive disorder-negative individuals |
| *LTBR* | 2 | Trauma exposure in major depressive disorder-negative individuals |
| *No data avaiable about RP1-102E24.8 in this dataset!* | NA | Trauma exposure in major depressive disorder-negative individuals |
| *CD27-AS1* | 0 | Trauma exposure in major depressive disorder-negative individuals |
| *TAPBPL* | 2 | Trauma exposure in major depressive disorder-negative individuals |
| *VAMP1* | 2 | Trauma exposure in major depressive disorder-negative individuals |
| *GAPDH* | 2 | Trauma exposure in major depressive disorder-negative individuals |
| *CHD4* | 0 | Insomnia |
| *LPAR5* | 3 | Insomnia |
| *No data avaiable about AC091814.2 in this dataset!* | NA | Insomnia |
| *CLEC12A* | 1 | Insomnia |
| *CLEC1B* | 1 | Insomnia |
| *CLEC12B* | 0 | Glycated hemoglobin levels in type 1 diabetes |
| *CLEC1A* | 1 | Essential hypertension (time to event) |
| *KLRD1* | 0 | Essential hypertension (time to event) |
| *KLRK1* | 0 | Essential hypertension (time to event) |
| *KLRC4* | 0 | Hyperlipidemia (time to event) |
| *KLRC3* | 1 | Osteoarthrosis (time to event) |
| *KLRC2* | 2 | Asthma (time to event) |
| *KLRC1* | 2 | Coronary atherosclerosis (time to event) |
| *H6PD* | 2 | Hematocrit |
| *SPSB1* | 1 | Hematocrit |
| *PIK3CD* | 3 | Hematocrit |
| *CTNNBIP1* | 0 | Hematocrit |
| *UBE4B* | 1 | Hematocrit |
| *RNU6-37P* | 0 | Hematocrit |
| *PGD* | 3 | Eosinophil counts |
| *DFFA* | 2 | Eosinophil counts |
| *C1orf127* | NA | Eosinophil counts |
| *TARDBP* | 2 | Eosinophil counts |
| *EXOSC10* | 2 | Eosinophil counts |
| *GPX7* | 1 | Eosinophil counts |
| *MIR5095* | 1 | Essential hypertension (time to event) |
| *SLC1A7* | 0 | Essential hypertension (time to event) |
| *CPT2* | 1 | Essential hypertension (time to event) |
| *LRP8* | 1 | hurdle binary method) |
| *GLIS1* | 1 | hurdle binary method) |
| *C8B* | 0 | Gut microbiota (bacterial taxa |
| *MYSM1* | 2 | hurdle binary method) |
| *C1orf87* | NA | Gut microbiota (bacterial taxa |
| *No data avaiable about INADL in this dataset!* | NA | hurdle binary method) |
| *L1TD1* | 2 | Gut microbiota (bacterial taxa |
| *KANK4* | 1 | hurdle binary method) |
| *USP10* | 4 | Gut microbiota (bacterial taxa |
| *No data avaiable about RP11-230B22.1 in this dataset!* | NA | hurdle binary method) |
| *LINC00466* | 1 | Gut microbiota (bacterial taxa |
| *CELSR2* | 2 | hurdle binary method) |
| *MYBPHL* | 1 | Gut microbiota (bacterial taxa |
| *PSMA5* | 3 | Gut microbiota (bacterial taxa |
| *ATXN7L2* | 2 | hurdle binary method) |
| *AMIGO1* | 1 | hurdle binary method) |
| *GNAI3* | 2 | Gut microbiota (bacterial taxa |
| *GNAT2* | 2 | Gut microbiota (bacterial taxa |
| *GSTM1* | 2 | Gut microbiota (bacterial taxa |
| *GSTM5* | 2 | hurdle binary method) |
| *GSTM3* | 1 | hurdle binary method) |
| *CSF1R* | 2 | hurdle binary method) |
| *ALX3* | 0 | Gut microbiota (bacterial taxa |
| *UBL4B* | 1 | Gut microbiota (bacterial taxa |
| *SYT6* | 0 | Gut microbiota (bacterial taxa |
| *AMPD1* | 1 | hurdle binary method) |
| *NRAS* | 2 | Coronary atherosclerosis (time to event) |
| *TSHB* | 2 | Type 2 diabetes (time to event) |
| *NGF* | 1 | Type 2 diabetes (time to event) |
| *VANGL1* | 2 | Waist-to-hip ratio adjusted for BMI |
| *NHLH2* | 1 | Waist-to-hip ratio adjusted for BMI |
| *SLC22A15* | 0 | Waist-to-hip ratio adjusted for BMI |
| *RHBG* | 1 | Waist-to-hip ratio adjusted for BMI |
| *IQGAP3* | 1 | Waist-to-hip ratio adjusted for BMI |
| *ISG20L2* | 2 | Waist-to-hip ratio adjusted for BMI |
| *NTRK1* | 1 | Waist-to-hip ratio adjusted for BMI |
| *PEAR1* | 1 | Waist-to-hip ratio adjusted for BMI |
| *ARHGEF11* | 1 | Waist-to-hip ratio adjusted for BMI |
| *UCHL5* | 3 | Waist-to-hip ratio adjusted for BMI |
| *GLRX2* | 1 | Rate of cognitive decline in Alzheimer's disease |
| *LINC01031* | 1 | Rate of cognitive decline in Alzheimer's disease |
| *RPL23AP22* | 0 | Rate of cognitive decline in Alzheimer's disease |
| *EEF1A1P14* | 0 | Rate of cognitive decline in Alzheimer's disease |
| *KCNT2* | 2 | Rate of cognitive decline in Alzheimer's disease |
| *CFH* | 2 | Rate of cognitive decline in Alzheimer's disease |
| *CFHR1* | 0 | Rate of cognitive decline in Alzheimer's disease |
| *CFHR2* | 0 | Rate of cognitive decline in Alzheimer's disease |
| *TNFSF13B* | 4 | Rate of cognitive decline in Alzheimer's disease |
| *CRB1* | 0 | Rate of cognitive decline in Alzheimer's disease |
| *DENND1B* | 1 | Rate of cognitive decline in Alzheimer's disease |
| *C1orf53* | NA | Rate of cognitive decline in Alzheimer's disease |
| *NEK7* | 1 | Rate of cognitive decline in Alzheimer's disease |
| *ADCK3* | 0 | Rate of cognitive decline in Alzheimer's disease |
| *CDC42BPA* | 0 | Rate of cognitive decline in Alzheimer's disease |
| *No data avaiable about CTD-2090I13.1 in this dataset!* | NA | Rate of cognitive decline in Alzheimer's disease |
| *ZNF678* | 1 | White matter microstructure (radial diusivities) |
| *SNAP47* | 1 | White matter microstructure (radial diusivities) |
| *WNT9A* | 0 | White matter microstructure (radial diusivities) |
| *ARF1* | 1 | White matter microstructure (radial diusivities) |
| *OBSCN* | 0 | White matter microstructure (radial diusivities) |
| *HIST3H2A* | 2 | White matter microstructure (radial diusivities) |
| *EXOC8* | 2 | White matter microstructure (radial diusivities) |
| *EGLN1* | 1 | White matter microstructure (radial diusivities) |
| *SIPA1L2* | 1 | White matter microstructure (radial diusivities) |
| *MAP10* | 2 | White matter microstructure (radial diusivities) |
| *No data avaiable about PCNXL2 in this dataset!* | NA | White matter microstructure (radial diusivities) |
| *SLC35F3* | 1 | Gut microbiota (bacterial taxa |
| *No data avaiable about RP5-827C21.1 in this dataset!* | NA | hurdle binary method) |
| *No data avaiable about U8 in this dataset!* | NA | hurdle binary method) |
| *LINC00184* | 1 | Gut microbiota (bacterial taxa |
| *No data avaiable about RP4-781K5.4 in this dataset!* | NA | Gut microbiota (bacterial taxa |
| *No data avaiable about RP4-781K5.6 in this dataset!* | NA | Gut microbiota (bacterial taxa |
| *No data avaiable about RP4-781K5.8 in this dataset!* | NA | hurdle binary method) |
| *No data avaiable about RP11-443B7.1 in this dataset!* | NA | hurdle binary method) |
| *TOMM20* | 1 | Gut microbiota (bacterial taxa |
| *B3GALNT2* | 0 | Gut microbiota (bacterial taxa |
| *HS1BP3* | 2 | hurdle binary method) |
| *GDF7* | 1 | Gut microbiota (bacterial taxa |
| *No data avaiable about C2ORF43 in this dataset!* | NA | Gut microbiota (bacterial taxa |
| *TDRD15* | 0 | hurdle binary method) |
| *KLHL29* | 0 | Waist-to-hip ratio adjusted for BMI |
| *ATAD2B* | 1 | Waist-to-hip ratio adjusted for BMI |
| *MFSD2B* | 0 | Waist-to-hip ratio adjusted for BMI |
| *No data avaiable about AC097724.3 in this dataset!* | NA | Waist-to-hip ratio adjusted for BMI |
| *TRMT61B* | 1 | Waist-to-hip ratio adjusted for BMI |
| *FAM179A* | 1 | Waist-to-hip ratio adjusted for BMI |
| *C2orf71* | NA | Receptive language ability |
| *ALK* | 3 | Receptive language ability |
| *YPEL5* | 1 | Parent of origin effect on receptive language ability (paternal) |
| *LCLAT1* | 0 | Parent of origin effect on receptive language ability (paternal) |
| *CAPN14* | 1 | Parent of origin effect on receptive language ability (paternal) |
| *EHD3* | 0 | Parent of origin effect on receptive language ability (paternal) |
| *XDH* | 3 | Parent of origin effect on receptive language ability (paternal) |
| *SLC30A6* | 2 | Parent of origin effect on receptive language ability (paternal) |
| *BIRC6* | 1 | Parent of origin effect on receptive language ability (maternal) |
| *LTBP1* | 1 | Eosinophil percentage of white cells |
| *No data avaiable about AC009499.1 in this dataset!* | NA | Eosinophil percentage of white cells |
| *No data avaiable about AC073218.1 in this dataset!* | NA | Hematocrit |
| *MRPL50P1* | 0 | Hematocrit |
| *FEZ2* | 2 | Hematocrit |
| *No data avaiable about AC007382.1 in this dataset!* | NA | Hematocrit |
| *HEATR5B* | 1 | Hematocrit |
| *EIF2AK2* | 0 | Hematocrit |
| *PRKD3* | 2 | White matter microstructure (radial diusivities) |
| *CDC42EP3* | 1 | Eosinophil percentage of white cells |
| *No data avaiable about AC010878.3 in this dataset!* | NA | Eosinophil percentage of white cells |
| *CYP1B1* | 1 | Deliberate self-harm |
| *ZNF638* | 2 | Deliberate self-harm |
| *EXOC6B* | 1 | Deliberate self-harm |
| *SFXN5* | 1 | Deliberate self-harm |
| *CCT7* | 1 | Deliberate self-harm |
| *NAT8* | 1 | White matter microstructure (fractional anisotropy) |
| *BOLA3* | 0 | White matter microstructure (fractional anisotropy) |
| *SH2D6* | 1 | White matter microstructure (fractional anisotropy) |
| *No data avaiable about RN7SL830P in this dataset!* | NA | White matter microstructure (fractional anisotropy) |
| *GGCX* | 3 | White matter microstructure (fractional anisotropy) |
| *USP39* | 1 | White matter microstructure (fractional anisotropy) |
| *SFTPB* | 1 | White matter microstructure (fractional anisotropy) |
| *GNLY* | 0 | White matter microstructure (fractional anisotropy) |
| *ATOH8* | 0 | White matter microstructure (fractional anisotropy) |
| *REEP1* | 2 | Lung function (FEV1/FVC) |
| *CHMP3* | 1 | Lung function (FEV1/FVC) |
| *RNF103* | 1 | Lung function (FEV1/FVC) |
| *CD8A* | 2 | Lung function (FEV1/FVC) |
| *No data avaiable about Z98044.1 in this dataset!* | NA | Lung function (FEV1/FVC) |
| *No data avaiable about RN7SL721P in this dataset!* | NA | Lung function (FEV1/FVC) |
| *PEX14* | 1 | Lung function (FEV1/FVC) |
| *CASZ1* | 0 | Lung function (FEV1/FVC) |
| *MASP2* | 0 | Hematocrit |
| *ZYG11B* | 2 | Hematocrit |
| *ZYG11A* | 0 | Hematocrit |
| *PODN* | 0 | Hematocrit |
| *DAB1* | 0 | Hematocrit |
| *FGGY* | 1 | Hematocrit |
| *HOOK1* | 1 | Hematocrit |
| *NFIA* | 1 | Hematocrit |
| *DOCK7* | 0 | Hematocrit |
| *ANGPTL3* | 1 | Hematocrit |
| *ATG4C* | 1 | Hematocrit |
| *ALG6* | 2 | Hematocrit |
| *PGM1* | 1 | Hematocrit |
| *ROR1* | 1 | Hematocrit |
| *TMEM167B* | 0 | Hematocrit |
| *KIAA1324* | 0 | Hematocrit |
| *PSRC1* | 1 | Hematocrit |
| *SORT1* | 3 | Hematocrit |
| *SYPL2* | 1 | Hematocrit |
| *CYB561D1* | 2 | Hematocrit |
| *GPR61* | 1 | Hematocrit |
| *AMPD2* | 2 | Hematocrit |
| *GSTM2* | 0 | Hematocrit |
| *EPS8L3* | 1 | Hematocrit |
| *No data avaiable about RP4-735C1.6 in this dataset!* | NA | Hematocrit |
| *No data avaiable about RP11-195M16.3 in this dataset!* | NA | Hematocrit |
| *No data avaiable about RP5-1028L10.2 in this dataset!* | NA | Hematocrit |
| *TRIM33* | 3 | White matter microstructure (axial diusivities) |
| *DENND2C* | 0 | Asthma |
| *TSPAN2* | 1 | Adolescent idiopathic scoliosis |
| *No data avaiable about RP4-663N10.1 in this dataset!* | NA | Adolescent idiopathic scoliosis |
| *CASQ2* | 2 | Adolescent idiopathic scoliosis |
| *No data avaiable about RP4-655J12.4 in this dataset!* | NA | Adolescent idiopathic scoliosis |
| *CCT3* | 1 | Adolescent idiopathic scoliosis |
| *MEF2D* | 2 | Adolescent idiopathic scoliosis |
| *BCAN* | 3 | Adolescent idiopathic scoliosis |
| *HDGF* | 1 | Adolescent idiopathic scoliosis |
| *SH2D2A* | 0 | Adolescent idiopathic scoliosis |
| *INSRR* | 1 | Adolescent idiopathic scoliosis |
| *LRRC71* | 1 | Adolescent idiopathic scoliosis |
| *ETV3L* | 0 | Adolescent idiopathic scoliosis |
| *CDC73* | 2 | Adolescent idiopathic scoliosis |
| *B3GALT2* | 1 | Adolescent idiopathic scoliosis |
| *No data avaiable about RP11-476H20.1 in this dataset!* | NA | Adolescent idiopathic scoliosis |
| *CFHR3* | 1 | Recurrence of Clostridium difficile infection x bezlotuxumab treatment interaction (2df test) |
| *CFHR4* | 1 | Asthma |
| *CFHR5* | 0 | Asthma |
| *ASPM* | 0 | Asthma |
| *ZBTB41* | 1 | Asthma |
| *LHX9* | 1 | Asthma |
| *PSEN2* | 2 | Asthma |
| *JMJD4* | 1 | Asthma |
| *PRSS38* | 1 | Asthma |
| *WNT3A* | 2 | Asthma |
| *GUK1* | 2 | Asthma |
| *TRIM11* | 2 | Chronic rhinosinusitis |
| *TRIM67* | 0 | Chronic rhinosinusitis |
| *TSNAX* | 3 | Chronic rhinosinusitis |
| *DISC1* | 0 | Nasal polyps |
| *No data avaiable about MLK4 in this dataset!* | NA | Nasal polyps |
| *KCNK1* | 2 | Nasal polyps |
| *IRF2BP2* | 0 | Nasal polyps |
| *No data avaiable about RP4-781K5.7 in this dataset!* | NA | Nasal polyps |
| *RNY4P16* | 0 | Asthma |
| *No data avaiable about RP11-443B7.3 in this dataset!* | NA | Asthma |
| *ARID4B* | 2 | Benign childhood epilepsy with centro-temporal spikes |
| *TBCE* | 1 | Benign childhood epilepsy with centro-temporal spikes |
| *LYST* | 0 | Benign childhood epilepsy with centro-temporal spikes |
| *No data avaiable about RP5-940F7.2 in this dataset!* | NA | Benign childhood epilepsy with centro-temporal spikes |
| *No data avaiable about AC012065.4 in this dataset!* | NA | Central corneal thickness |
| *No data avaiable about AC012361.1 in this dataset!* | NA | Central corneal thickness |
| *APOB* | 1 | Central corneal thickness |
| *No data avaiable about AC096570.1 in this dataset!* | NA | Central corneal thickness |
| *UBXN2A* | 1 | Central corneal thickness |
| *FKBP1B* | 1 | Central corneal thickness |
| *DYSF* | 3 | Central corneal thickness |
| *CYP26B1* | 3 | Central corneal thickness |
| *EMX1* | 0 | White matter microstructure (radial diusivities) |
| *RAB11FIP5* | 2 | White matter microstructure (radial diusivities) |
| *NOTO* | 0 | White matter microstructure (radial diusivities) |
| *FBXO41* | 1 | White matter microstructure (radial diusivities) |
| *ALMS1* | 2 | White matter microstructure (radial diusivities) |
| *TPRKB* | 1 | White matter microstructure (radial diusivities) |
| *DUSP11* | 3 | White matter microstructure (radial diusivities) |
| *STAMBP* | 1 | White matter microstructure (radial diusivities) |
| *ACTG2* | 1 | White matter microstructure (radial diusivities) |
| *TET3* | 0 | Rate of cognitive decline in Alzheimer's disease |
| *SLC4A5* | 0 | Rate of cognitive decline in Alzheimer's disease |
| *MYOM3* | 1 | Rate of cognitive decline in Alzheimer's disease |
| *IFNLR1* | 0 | Rate of cognitive decline in Alzheimer's disease |
| *GRHL3* | 1 | Rate of cognitive decline in Alzheimer's disease |
| *NIPAL3* | 1 | Rate of cognitive decline in Alzheimer's disease |
| *SRRM1* | 1 | Rate of cognitive decline in Alzheimer's disease |
| *CLIC4* | 2 | Rate of cognitive decline in Alzheimer's disease |
| *RUNX3* | 0 | Rate of cognitive decline in Alzheimer's disease |
| *No data avaiable about RP11-84D1.2 in this dataset!* | NA | Rate of cognitive decline in Alzheimer's disease |
| *SYF2* | 1 | Rate of cognitive decline in Alzheimer's disease |
| *No data avaiable about C1ORF63 in this dataset!* | NA | Rate of cognitive decline in Alzheimer's disease |
| *TMEM50A* | 0 | Rate of cognitive decline in Alzheimer's disease |
| *RHCE* | 1 | Rate of cognitive decline in Alzheimer's disease |
| *FAM69A* | 0 | Rate of cognitive decline in Alzheimer's disease |
| *CCDC18* | 1 | Rate of cognitive decline in Alzheimer's disease |
| *DR1* | 0 | Rate of cognitive decline in Alzheimer's disease |
| *FNBP1L* | 0 | Rate of cognitive decline in Alzheimer's disease |
| *BCAR3* | 0 | Rate of cognitive decline in Alzheimer's disease |
| *DNTTIP2* | 1 | Hematocrit |
| *GCLM* | 1 | Hematocrit |
| *PI4KB* | 1 | Hematocrit |
| *LCE5A* | 1 | Hematocrit |
| *CRCT1* | 0 | Hematocrit |
| *LCE3E* | 1 | Hematocrit |
| *LCE3D* | 1 | Hematocrit |
| *LCE3B* | 1 | Hematocrit |
| *LCE3A* | 0 | Hematocrit |
| *LCE1E* | 1 | Hematocrit |
| *IVL* | 0 | Hematocrit |
| *SPRR2A* | 2 | Hematocrit |
| *SPRR2G* | 1 | hurdle binary method) |
| *NPR1* | 2 | Gut microbiota (bacterial taxa |
| *SLC27A3* | 1 | hurdle binary method) |
| *VANGL2* | 0 | Gut microbiota (bacterial taxa |
| *RALGPS2* | 2 | hurdle binary method) |
| *ANGPTL1* | 2 | Gut microbiota (bacterial taxa |
| *ABL2* | 2 | hurdle binary method) |
| *COX5BP8* | 0 | hurdle binary method) |
| *SOAT1* | 1 | Gut microbiota (bacterial taxa |
| *AXDND1* | 0 | hurdle binary method) |
| *ESRRG* | 2 | Gut microbiota (bacterial taxa |
| *GPATCH2* | 2 | hurdle binary method) |
| *SPATA17* | 1 | Gut microbiota (bacterial taxa |
| *RRP15* | 1 | hurdle binary method) |
| *TGFB2* | 2 | White matter microstructure (radial diusivities) |
| *LYPLAL1* | 3 | White matter microstructure (radial diusivities) |
| *SNTG2* | 1 | White matter microstructure (radial diusivities) |
| *TPO* | 2 | White matter microstructure (radial diusivities) |
| *PXDN* | 3 | White matter microstructure (radial diusivities) |
| *TSSC1* | 2 | White matter microstructure (radial diusivities) |
| *TRAPPC12* | 0 | White matter microstructure (radial diusivities) |
| *ADI1* | 1 | Waist-to-hip ratio adjusted for BMI |
| *RPS7* | 1 | Waist-to-hip ratio adjusted for BMI |
| *COLEC11* | 0 | Waist-to-hip ratio adjusted for BMI |
| *ALLC* | 2 | Waist-to-hip ratio adjusted for BMI |
| *GAPDHP48* | 0 | Waist-to-hip ratio adjusted for BMI |
| *DCDC2C* | 0 | Autism spectrum disorders (social interaction) |
| *NPM1P48* | 0 | Autism spectrum disorders (social interaction) |
| *No data avaiable about AC019198.1 in this dataset!* | NA | Autism spectrum disorders (social interaction) |
| *No data avaiable about SMEK2 in this dataset!* | NA | Autism spectrum disorders (peer interaction) |
| *PNPT1* | 0 | Autism spectrum disorders (repetitive sensory-motor behaviour) |
| *EFEMP1* | 2 | Autism spectrum disorders (repetitive sensory-motor behaviour) |
| *No data avaiable about RP11-481J13.1 in this dataset!* | NA | Hematocrit |
| *MIR216B* | 0 | Hematocrit |
| *CCDC85A* | 1 | Hematocrit |
| *No data avaiable about AC008173.1 in this dataset!* | NA | Hematocrit |
| *No data avaiable about RP11-443I9.1 in this dataset!* | NA | Hematocrit |
| *VRK2* | 2 | Hematocrit |
| *FANCL* | 1 | Hematocrit |
| *EIF3FP3* | 0 | Hematocrit |
| *No data avaiable about AC007092.1 in this dataset!* | NA | Hematocrit |
| *No data avaiable about RP11-444A22.1 in this dataset!* | NA | Hematocrit |
| *No data avaiable about AC007381.3 in this dataset!* | NA | White matter microstructure (radial diusivities) |
| *No data avaiable about AC007381.2 in this dataset!* | NA | White matter microstructure (radial diusivities) |
| *MIR4432* | 0 | White matter microstructure (radial diusivities) |
| *BCL11A* | 1 | White matter microstructure (radial diusivities) |
| *MTUS2* | 1 | White matter microstructure (radial diusivities) |
| *KCNK13* | 2 | Rheumatic heart disease |
| *CALM1* | 2 | Asthma |
| *TTC7B* | 1 | NA |
| *RPS6KA5* | 1 | Systolic blood pressure |
| *C14orf159* | NA | Help-seeking from a GP |
| *CCDC88C* | 1 | Help-seeking from a GP |
| *No data avaiable about RP11-895M11.3 in this dataset!* | NA | Help-seeking from a GP |
| *No data avaiable about SMEK1 in this dataset!* | NA | Help-seeking from a GP |
| *No data avaiable about AL133373.1 in this dataset!* | NA | Help-seeking from a GP |
| *CATSPERB* | 1 | Help-seeking from a GP |
| *TC2N* | 1 | Neuroticism |
| *FBLN5* | 1 | Neuroticism |
| *TRIP11* | 1 | Major depressive disorder (lifetime) |
| *ATXN3* | 3 | Help-seeking from a psychiatrist |
| *NDUFB1* | 1 | Help-seeking from a psychiatrist |
| *CPSF2* | 2 | Help-seeking from a GP |
| *No data avaiable about RP11-472N19.3 in this dataset!* | NA | Help-seeking from a GP |
| *RIN3* | 1 | Help-seeking from a GP |
| *LGMN* | 3 | Hemoglobin concentration |
| *CHGA* | 3 | Hemoglobin concentration |
| *ITPK1* | 2 | Hemoglobin concentration |
| *No data avaiable about RP11-371E8.2 in this dataset!* | NA | Hemoglobin concentration |
| *TMEM251* | 0 | Hemoglobin concentration |
| *BTBD7* | 1 | Hemoglobin concentration |
| *UNC79* | 1 | Hemoglobin concentration |
| *PRIMA1* | 3 | Hemoglobin concentration |
| *ASB2* | 2 | Hemoglobin concentration |
| *LINC00521* | 1 | Hemoglobin concentration |
| *DDX24* | 2 | Hemoglobin concentration |
| *PPP4R4* | 1 | Hemoglobin concentration |
| *SERPINA10* | 1 | Hemoglobin concentration |
| *SERPINA6* | 1 | Deliberate self-harm |
| *SERPINA10* | 1 | Deliberate self-harm |
| *SERPINA11* | 0 | Deliberate self-harm |
| *SERPINA9* | 2 | Deliberate self-harm |
| *SERPINA12* | 0 | Deliberate self-harm |
| *SERPINA4* | 1 | Waist-to-hip ratio adjusted for BMI |
| *SERPINA5* | 0 | Waist-to-hip ratio adjusted for BMI |
| *SERPINA3* | 1 | Waist-to-hip ratio adjusted for BMI |
| *SERPINA13P* | 0 | Waist-to-hip ratio adjusted for BMI |
| *GSC* | 0 | Caffeine consumption from tea |
| *DICER1* | 1 | Caffeine consumption from tea |
| *CLMN* | 1 | Caffeine consumption from tea |
| *No data avaiable about RP11-1070N10.3 in this dataset!* | NA | Caffeine consumption from tea |
| *LIN28A* | 0 | Caffeine consumption from tea |
| *DHDDS* | 2 | Caffeine consumption from coffee or tea |
| *DPPA2P2* | 0 | Caffeine consumption from coffee |
| *RPS6KA1* | 3 | Caffeine consumption from coffee |
| *NDC1* | 0 | Caffeine consumption from coffee |
| *No data avaiable about RP11-339A11.1 in this dataset!* | NA | Caffeine consumption from coffee |
| *ADIPOR1* | 2 | Caffeine consumption from coffee |
| *PPFIA4* | 0 | Caffeine consumption from coffee |
| *ADORA1* | 3 | Caffeine consumption from coffee |
| *CHI3L1* | 3 | Caffeine consumption from coffee |
| *CHIT1* | 0 | Peak expiratory flow |
| *BTG2* | 0 | Peak expiratory flow |
| *FMOD* | 0 | Peak expiratory flow |
| *PPIAP7* | 0 | Peak expiratory flow |
| *OLFM3* | 1 | Peak expiratory flow |
| *HIST2H4B* | 0 | Peak expiratory flow |
| *HIST2H2BE* | 1 | Peak expiratory flow |
| *SV2A* | 3 | Peak expiratory flow |
| *SF3B4* | 1 | Eosinophil counts |
| *MTMR11* | 2 | Eosinophil counts |
| *VPS45* | 3 | Eosinophil counts |
| *ANP32E* | 2 | Eosinophil counts |
| *No data avaiable about KIAA1731 in this dataset!* | NA | Eosinophil counts |
| *No data avaiable about HPRTP4 in this dataset!* | NA | Eosinophil counts |
| *KDM4D* | 0 | Trauma exposure |
| *ENDOD1* | 2 | Trauma exposure |
| *No data avaiable about RP11-712B9.2 in this dataset!* | NA | Trauma exposure |
| *SESN3* | 2 | Trauma exposure |
| *No data avaiable about RP1-91G5.3 in this dataset!* | NA | Trauma exposure |
| *S100A9* | 2 | Trauma exposure |
| *S100A12* | 0 | Trauma exposure |
| *No data avaiable about AL591704.5 in this dataset!* | NA | Trauma exposure |
| *S100A7A* | 1 | Insomnia |
| *S100A7A* | 1 | Insomnia |
| *S100A6* | 3 | Insomnia |
| *S100A5* | 2 | Insomnia |
| *S100A4* | 2 | Insomnia |
| *NUP210L* | 0 | Insomnia |
| *TPM3* | 1 | Blood protein levels |
| *C1orf189* | NA | Blood protein levels |
| *UBAP2L* | 1 | Blood protein levels |
| *HAX1* | 1 | Blood protein levels |
| *No data avaiable about SNORD59 in this dataset!* | NA | Blood protein levels |
| *AQP10* | 0 | Blood protein levels |
| *ATP8B2* | 0 | Blood protein levels |
| *No data avaiable about RP11-317P15.6 in this dataset!* | NA | Blood protein levels |
| *NPL* | 0 | Insomnia |
| *SHCBP1L* | 1 | Insomnia |
| *LAMC1* | 3 | Insomnia |
| *LAMC2* | 3 | Insomnia |
| *NMNAT2* | 1 | Insomnia |
| *SMG7-AS1* | 0 | Insomnia |
| *SMG7-AS1* | 0 | Insomnia |
| *NCF2* | 0 | Insomnia |
| *RGL1* | 1 | Insomnia |
| *APOBEC4* | 1 | White matter microstructure (axial diusivities) |
| *COLGALT2* | 0 | White matter microstructure (axial diusivities) |
| *TSEN15* | 0 | White matter microstructure (axial diusivities) |
| *C1orf21* | NA | White matter microstructure (axial diusivities) |
| *EDEM3* | 0 | Venous thromboembolism |
| *FAM129A* | 1 | Venous thromboembolism |
| *RNF2* | 1 | Venous thromboembolism |
| *TRMT1L* | 1 | Venous thromboembolism |
| *SWT1* | 1 | Venous thromboembolism |
| *TMEM200A* | 1 | Venous thromboembolism |
| *EPB41L2* | 1 | General cognitive ability |
| *NCSTN* | 2 | General cognitive ability |
| *MRPS14* | 1 | General cognitive ability |
| *SDCCAG3P2* | 0 | General cognitive ability |
| *CTNNA1* | 4 | General cognitive ability |
| *KIAA0040* | 1 | General cognitive ability |
| *TNR* | 1 | General cognitive ability |
| *RFWD2* | 1 | General cognitive ability |
| *H19* | 2 | General cognitive ability |
| *IGF2* | 2 | Asthma |
| *INS-IGF2* | 1 | Asthma |
| *INSRR* | 1 | Asthma |
| *THBS2* | 4 | Asthma |
| *MIR4686* | 0 | Asthma |
| *ASCL2* | 2 | Asthma |
| *C11orf21* | NA | Hematocrit |
| *TSPAN32* | 2 | Hematocrit |
| *ROCK1* | 2 | Hematocrit |
| *ABHD3* | 2 | Hematocrit |
| *No data avaiable about RP11-595B24.2 in this dataset!* | NA | Eosinophil counts |
| *RNU6ATAC20P* | 0 | Eosinophil counts |
| *GATA6* | 1 | Eosinophil counts |
| *CTAGE1* | 0 | Eosinophil counts |
| *RBBP8* | 2 | Eosinophil counts |
| *CABLES1* | 1 | Eosinophil counts |
| *TMEM241* | 1 | Eosinophil counts |
| *RIOK3* | 0 | Crohn's disease |
| *C18orf8* | NA | Crohn's disease |
| *TTR* | 2 | Crohn's disease |
| *B4GALT6* | 1 | Crohn's disease |
| *LHB* | 0 | Crohn's disease |
| *No data avaiable about CGB in this dataset!* | NA | Crohn's disease |
| *CGB8* | 0 | Crohn's disease |
| *CGB7* | 0 | Crohn's disease |
| *NTF4* | 2 | Central corneal thickness |
| *SNRNP70* | 0 | Central corneal thickness |
| *PPFIA3* | 1 | Central corneal thickness |
| *SLC6A16* | 0 | Central corneal thickness |
| *DKKL1* | 1 | Central corneal thickness |
| *No data avaiable about CTD-3148I10.1 in this dataset!* | NA | Central corneal thickness |
| *ALDH16A1* | 0 | Central corneal thickness |
| *FLT3LG* | 1 | Central corneal thickness |
| *FCGRT* | 2 | Central corneal thickness |
| *RCN3* | 2 | Central corneal thickness |
| *NOSIP* | 2 | Central corneal thickness |
| *PRRG2* | 1 | Central corneal thickness |
| *PRR12* | 0 | Central corneal thickness |
| *RRAS* | 3 | Central corneal thickness |
| *SCAF1* | 0 | Central corneal thickness |
| *CPT1C* | 1 | Central corneal thickness |
| *No data avaiable about CTB-33G10.1 in this dataset!* | NA | Central corneal thickness |
| *TSKS* | 1 | Central corneal thickness |
| *MED25* | 3 | Central corneal thickness |
| *RN7SKP229* | 0 | White matter microstructure (radial diusivities) |
| *ZNF648* | 1 | White matter microstructure (radial diusivities) |
| *No data avaiable about GS1-122H1.2 in this dataset!* | NA | White matter microstructure (radial diusivities) |
| *GLUL* | 1 | White matter microstructure (radial diusivities) |
| *TEDDM1* | 0 | White matter microstructure (radial diusivities) |
| *RGSL1* | 1 | Hematocrit |
| *RNASEL* | 3 | Hematocrit |
| *RGS16* | 2 | Eosinophil counts |
| *No data avaiable about RP11-532L16.3 in this dataset!* | NA | Eosinophil counts |
| *ZNF281* | 1 | Eosinophil counts |
| *MROH3P* | 0 | Response to antiepileptic mood-stabilizing treatment in bipolar disorder |
| *CSPG5* | 1 | Response to antiepileptic mood-stabilizing treatment in bipolar disorder |
| *SMARCC1* | 3 | Gut microbiota (bacterial taxa |
| *ARIH2OS* | 0 | Gut microbiota (bacterial taxa |
| *ARIH2OS* | 0 | Gut microbiota (bacterial taxa |
| *ACTBP13* | 0 | hurdle binary method) |
| *MST1R* | 1 | hurdle binary method) |
| *No data avaiable about CTD-2330K9.3 in this dataset!* | NA | Gut microbiota (bacterial taxa |
| *MON1A* | 1 | Gut microbiota (bacterial taxa |
| *RBM6* | 1 | Gut microbiota (bacterial taxa |
| *RBM5* | 0 | hurdle binary method) |
| *No data avaiable about RP11-493K19.3 in this dataset!* | NA | Gut microbiota (bacterial taxa |
| *SEMA3F* | 1 | Gut microbiota (bacterial taxa |
| *SETD4* | 1 | hurdle binary method) |
| *CBR1* | 3 | Gut microbiota (bacterial taxa |
| *MUSTN1* | 0 | hurdle binary method) |
| *TMEM110* | 0 | Gut microbiota (bacterial taxa |
| *SFMBT1* | 1 | hurdle binary method) |
| *RFT1* | 2 | Gut microbiota (bacterial taxa |
| *PRKCD* | 2 | hurdle binary method) |
| *TKT* | 2 | hurdle binary method) |
| *DCP1A* | 1 | Gut microbiota (bacterial taxa |
| *IL17RB* | 2 | Gut microbiota (bacterial taxa |
| *SELK* | 3 | Gut microbiota (bacterial taxa |
| *CABYRP1* | 0 | hurdle binary method) |
| *CACNA2D3* | 3 | Gut microbiota (bacterial taxa |
| *LRTM1* | 1 | Blood protein levels |
| *No data avaiable about RP11-889D3.1 in this dataset!* | NA | Blood protein levels |
| *WNT5A* | 1 | Blood protein levels |
| *ERC2* | 0 | Blood protein levels |
| *FOXP1* | 1 | Blood protein levels |
| *EIF4E3* | 2 | Blood protein levels |
| *CCDC66* | 0 | Blood protein levels |
| *No data avaiable about RP11-648C16.1 in this dataset!* | NA | Blood protein levels |
| *LINC00877* | 0 | Blood protein levels |
| *PLSCR5* | 1 | CV-A6-associated hand |
| *FAM208A* | 2 | and mouth disease (severe vs mild) |
| *ARHGEF3* | 2 | CV-A6-associated hand |
| *IL17RD* | 1 | and mouth disease (severe vs mild) |
| *DNAH12* | 0 | and mouth disease (severe vs mild) |
| *SLMAP* | 1 | foot |
| *FLNB* | 2 | and mouth disease (severe vs mild) |
| *DNASE1L3* | 1 | CV-A6-associated hand |
| *ABHD6* | 1 | and mouth disease (severe vs mild) |
| *RPP14* | 1 | Hematocrit |
| *No data avaiable about RP11-80H18.4 in this dataset!* | NA | Hematocrit |
| *PXK* | 2 | Hematocrit |
| *KCTD6* | 2 | Hematocrit |
| *FAM3D* | 0 | Hematocrit |
| *PDHB* | 2 | Hematocrit |
| *ACOX2* | 1 | Hematocrit |
| *FAM107A* | 1 | Hematocrit |
| *C3orf67* | NA | Hematocrit |
| *FHIT* | 1 | Adenosine Deaminase levels |
| *FAM19A4* | 0 | CCL25 levels |
| *TMF1* | 1 | Cystatin D levels |
| *ARL6IP5* | 2 | interleukin 15 receptor subunit alpha levels |
| *FRMD4B* | 1 | interleukin-18 receptor 1 levels |
| *MITF* | 2 | Monocyte chemoattractant protein-4 levels |
| *ARHGEF26-AS1* | 0 | Monocyte chemoattractant protein-4 levels |
| *ARHGEF26-AS1* | 0 | Tumor necrosis factor beta levels |
| *DHX36* | 3 | Acute anterior uveitis in ankylosing spondylitis |
| *GPR149* | 0 | Peginterferon alfa-2a treatment response in chronic hepatitis B infection |
| *TPRA1* | 0 | Peginterferon alfa-2a treatment response in chronic hepatitis B infection |
| *MCM2* | 0 | Peginterferon alfa-2a treatment response in chronic hepatitis B infection |
| *MGLL* | 3 | Peginterferon alfa-2a treatment response in chronic hepatitis B infection |
| *KBTBD12* | 2 | Peginterferon alfa-2a treatment response in chronic hepatitis B infection |
| *WNT7B* | 2 | Peginterferon alfa-2a treatment response in chronic hepatitis B infection |
| *ZIC4* | 1 | Peginterferon alfa-2a treatment response in chronic hepatitis B infection |
| *ZIC1* | 0 | Waist-to-hip ratio adjusted for BMI |
| *CPB1* | 0 | Waist-to-hip ratio adjusted for BMI |
| *LINC00880* | 0 | Waist-to-hip ratio adjusted for BMI |
| *No data avaiable about RP11-6F2.5 in this dataset!* | NA | Waist-to-hip ratio adjusted for BMI |
| *CCNL1* | 1 | Waist-to-hip ratio adjusted for BMI |
| *No data avaiable about RP11-550I24.2 in this dataset!* | NA | Waist-to-hip ratio adjusted for BMI |
| *VEPH1* | 1 | Waist-to-hip ratio adjusted for BMI |
| *No data avaiable about C3ORF55 in this dataset!* | NA | Waist-to-hip ratio adjusted for BMI |
| *SHOX2* | 0 | Waist-to-hip ratio adjusted for BMI |
| *SPATA16* | 2 | White matter microstructure (radial diusivities) |
| *NLGN1* | 2 | White matter microstructure (radial diusivities) |
| *No data avaiable about RP11-225N10.1 in this dataset!* | NA | White matter microstructure (radial diusivities) |
| *ATP11B* | 1 | White matter microstructure (radial diusivities) |
| *No data avaiable about RP11-531F16.4 in this dataset!* | NA | White matter microstructure (radial diusivities) |
| *MCCC1* | 0 | White matter microstructure (radial diusivities) |
| *MCF2L2* | 0 | White matter microstructure (radial diusivities) |
| *KLHL6* | 3 | White matter microstructure (radial diusivities) |
| *ZNF679* | 2 | White matter microstructure (radial diusivities) |
| *ZNF680* | 1 | White matter microstructure (radial diusivities) |
| *ZNF107* | 1 | Type 2 diabetes |
| *ZNF273* | 1 | Type 2 diabetes |
| *CLDN3* | 1 | Type 2 diabetes |
| *WBSCR28* | 1 | Type 2 diabetes |
| *RELN* | 4 | Type 2 diabetes |
| *LIMK1* | 1 | Type 2 diabetes |
| *LAT2* | 1 | Type 2 diabetes |
| *CLIP2* | 1 | Type 2 diabetes |
| *GTF2IRD1P1* | 0 | Type 2 diabetes |
| *GTF2IRD1P1* | 0 | Type 2 diabetes |
| *STAG3L2* | 0 | Parkinson's disease progression (motor) |
| *GTF2IRD2* | 1 | Parkinson's disease progression (cognitive) |
| *PMS2P5* | 1 | Parkinson's disease progression (cognitive) |
| *No data avaiable about GATSL1 in this dataset!* | NA | Parkinson's disease progression (cognitive) |
| *IFRD1* | 0 | Parkinson's disease progression (cognitive) |
| *GPR85* | 1 | Parkinson's disease progression (cognitive) |
| *No data avaiable about RP11-736E3.1 in this dataset!* | NA | Parkinson's disease progression (composite) |
| *TSRM* | 0 | Parkinson's disease progression (composite) |
| *PPP1R3A* | 1 | Cardiovascular death or myocardial infarction in response to clopidogrel treatment |
| *FOXP2* | 2 | Cardiovascular death or myocardial infarction in response to clopidogrel treatment |
| *MDFIC* | 2 | Platelet reactivity in response to clopidogrel treatment |
| *LMOD2* | 0 | Cardiovascular death |
| *WASL* | 2 | myocardial infarction or stroke in response to clopidogrel treatment |
| *RNU6-11P* | 0 | Cardiovascular death |
| *HYAL4* | 1 | myocardial infarction or stroke in response to clopidogrel treatment |
| *SPAM1* | 0 | Cardiovascular death |
| *GPR37* | 0 | Stent thrombosis in response to clopidogrel treatment |
| *POT1* | 1 | Stent thrombosis in response to clopidogrel treatment |
| *STX3* | 1 | Stent thrombosis in response to clopidogrel treatment |
| *TCN1* | 1 | Stent thrombosis in response to clopidogrel treatment |
| *MS4A3* | 2 | Platelet reactivity in response to clopidogrel treatment |
| *MS4A2* | 2 | Diabetic neuropathy in type 2 diabetes |
| *MS4A4E* | 0 | Diabetic neuropathy in type 2 diabetes |
| *MS4A4A* | 2 | Macrovascular complications in type 2 diabetes |
| *MS4A6E* | 1 | Macrovascular complications in type 2 diabetes |
| *MS4A7* | 3 | Macrovascular complications in type 2 diabetes |
| *MS4A14* | 1 | Macrovascular complications in type 2 diabetes |
| *MS4A5* | 1 | Macrovascular complications in type 2 diabetes |
| *MS4A14* | 1 | Platelet reactivity in response to clopidogrel treatment |
| *SLC22A9* | 1 | Cardiovascular death or myocardial infarction in response to clopidogrel treatment |
| *HRASLS5* | 1 | Platelet reactivity in response to clopidogrel treatment |
| *HRASLS2* | 1 | Platelet reactivity in response to clopidogrel treatment |
| *C11orf84* | NA | Deliberate self-harm |
| *MARK2* | 3 | Deliberate self-harm |
| *MAP4K2* | 2 | Deliberate self-harm |
| *No data avaiable about RP11-353P15.1 in this dataset!* | NA | Deliberate self-harm |
| *No data avaiable about RP11-789A21.1 in this dataset!* | NA | Deliberate self-harm |
| *GPR65* | 2 | Deliberate self-harm |
| *No data avaiable about RP11-300J18.1 in this dataset!* | NA | Deliberate self-harm |
| *KCNK10* | 2 | Deliberate self-harm |
| *SPATA7* | 2 | Deliberate self-harm |
| *ZC3H14* | 2 | Deliberate self-harm |
| *TTC8* | 1 | White matter microstructure (mode of anisotropy) |
| *FOXN3* | 1 | White matter microstructure (mode of anisotropy) |
| *No data avaiable about RP1-168P16.2 in this dataset!* | NA | White matter microstructure (mode of anisotropy) |
| *SPG7* | 1 | White matter microstructure (mode of anisotropy) |
| *CPNE7* | 3 | White matter microstructure (mode of anisotropy) |
| *DPEP1* | 1 | White matter microstructure (mode of anisotropy) |
| *CHMP1A* | 0 | White matter microstructure (mode of anisotropy) |
| *SPATA33* | 0 | White matter microstructure (mode of anisotropy) |
| *CDK10* | 1 | White matter microstructure (mode of anisotropy) |
| *SPATA2L* | 0 | White matter microstructure (mode of anisotropy) |
| *VPS9D1* | 0 | White matter microstructure (mode of anisotropy) |
| *ZNF276* | 1 | White matter microstructure (mode of anisotropy) |
| *FANCA* | 1 | White matter microstructure (mode of anisotropy) |
| *SPIRE2* | 3 | White matter microstructure (mode of anisotropy) |
| *TCF25* | 2 | White matter microstructure (mode of anisotropy) |
| *MC1R* | 2 | Reaction time |
| *TUBB3* | 2 | Reaction time |
| *No data avaiable about RP5-1029F21.3 in this dataset!* | NA | Reaction time |
| *VPS53* | 2 | Reaction time |
| *NXN* | 2 | Reaction time |
| *GABRB2* | 4 | General cognitive ability |
| *YWHAE* | 2 | General cognitive ability |
| *OTOP2* | 2 | General cognitive ability |
| *OTOP3* | 0 | General cognitive ability |
| *KCTD2* | 1 | General cognitive ability |
| *No data avaiable about AC111186.1 in this dataset!* | NA | General cognitive ability |
| *NT5C* | 2 | General cognitive ability |
| *SUMO2* | 1 | Asthma |
| *H3F3B* | 0 | Asthma |
| *UNK* | 1 | Asthma |
| *UNC13D* | 2 | Asthma |
| *WBP2* | 2 | Asthma |
| *TRIM47* | 2 | Asthma |
| *TRIM65* | 2 | Peak expiratory flow |
| *FBF1* | 0 | Peak expiratory flow |
| *ACOX1* | 2 | Peak expiratory flow |
| *TEN1* | 0 | Peak expiratory flow |
| *EVPL* | 1 | Peak expiratory flow |
| *EXOC7* | 1 | Peak expiratory flow |
| *RNF157* | 1 | Peak expiratory flow |
| *CDH19* | 2 | Blood protein levels |
| *DSEL* | 1 | Blood protein levels |
| *No data avaiable about RP11-638L3.1 in this dataset!* | NA | Blood protein levels |
| *TMX3* | 1 | Blood protein levels |
| *CCDC102B* | 2 | Blood protein levels |
| *DOK6* | 2 | Blood protein levels |
| *CD226* | 1 | Blood protein levels |
| *RTTN* | 1 | Hematocrit |
| *SOCS6* | 1 | Hematocrit |
| *No data avaiable about RP11-41O4.3 in this dataset!* | NA | Hematocrit |
| *GTSCR1* | 0 | Hematocrit |
| *INSRR* | 1 | Hematocrit |
| *No data avaiable about CTB-133G6.1 in this dataset!* | NA | Hematocrit |
| *PEX11G* | 1 | Peak expiratory flow |
| *ZNF358* | 1 | Peak expiratory flow |
| *MCOLN1* | 2 | Trauma exposure in major depressive disorder-negative individuals |
| *PCP2* | 1 | White matter microstructure (radial diusivities) |
| *RETN* | 2 | White matter microstructure (radial diusivities) |
| *FCER2* | 0 | White matter microstructure (radial diusivities) |
| *CLEC4G* | 1 | White matter microstructure (radial diusivities) |
| *CD209* | 1 | White matter microstructure (radial diusivities) |
| *CLEC4M* | 0 | White matter microstructure (radial diusivities) |
| *CLEC4GP1* | 0 | White matter microstructure (radial diusivities) |
| *EXOSC3P2* | 0 | White matter microstructure (radial diusivities) |
| *EVI5L* | 1 | Adolescent idiopathic scoliosis |
| *MAP2K7* | 3 | Adolescent idiopathic scoliosis |
| *SNAPC2* | 2 | Adolescent idiopathic scoliosis |
| *No data avaiable about CTC-525D6.1 in this dataset!* | NA | Adolescent idiopathic scoliosis |
| *VSTM2B* | 0 | Adolescent idiopathic scoliosis |
| *POP4* | 1 | Adolescent idiopathic scoliosis |
| *C19orf12* | NA | Ease of getting up in the morning |
| *CCNE1* | 2 | Ease of getting up in the morning |
| *URI1* | 0 | Ease of getting up in the morning |
| *ZNF536* | 1 | Ease of getting up in the morning |
| *TSHZ3* | 1 | hurdle binary method) |
| *No data avaiable about AC011518.1 in this dataset!* | NA | Gut microbiota (bacterial taxa |
| *ZNF507* | 0 | Gut microbiota (bacterial taxa |
| *DPY19L3* | 1 | Gut microbiota (bacterial taxa |
| *PDCD5* | 2 | Gut microbiota (bacterial taxa |
| *ANKRD27* | 1 | Gut microbiota (bacterial taxa |
| *ECH1* | 2 | hurdle binary method) |
| *HNRNPL* | 0 | hurdle binary method) |
| *IFNL4* | 0 | Gut microbiota (bacterial taxa |
| *IFNL2* | 1 | hurdle binary method) |
| *SAMD4B* | 0 | Gut microbiota (bacterial taxa |
| *SUPT5H* | 3 | hurdle binary method) |
| *SLC1A5* | 1 | Gut microbiota (bacterial taxa |
| *AP2S1* | 3 | hurdle binary method) |
| *ARHGAP35* | 1 | Gut microbiota (bacterial taxa |
| *TMEM160* | 2 | Chronotype (sMEQ score) |
| *ZC3H4* | 1 | Evening vs. morning chronotype (self-assessed) |
| *SAE1* | 1 | Rate of cognitive decline in Alzheimer's disease |
| *BBC3* | 2 | Rate of cognitive decline in Alzheimer's disease |
| *CCDC9* | 2 | Rate of cognitive decline in Alzheimer's disease |
| *C5AR1* | 1 | Rate of cognitive decline in Alzheimer's disease |
| *SLC8A2* | 1 | Rate of cognitive decline in Alzheimer's disease |
| *KPTN* | 2 | Rate of cognitive decline in Alzheimer's disease |
| *NAPA-AS1* | 0 | Rate of cognitive decline in Alzheimer's disease |
| *NAPA-AS1* | 0 | White matter microstructure (radial diusivities) |
| *GLTSCR1* | 2 | White matter microstructure (radial diusivities) |
| *RPL23AP80* | 0 | White matter microstructure (radial diusivities) |
| *SULT2A1* | 1 | White matter microstructure (radial diusivities) |
| *ELSPBP1* | 1 | White matter microstructure (radial diusivities) |
| *CABP5* | 0 | White matter microstructure (radial diusivities) |
| *NANP* | 0 | White matter microstructure (radial diusivities) |
| *ZNF337* | 0 | White matter microstructure (radial diusivities) |
| *FAM182B* | 1 | White matter microstructure (radial diusivities) |
| *No data avaiable about RP4-610C12.1 in this dataset!* | NA | White matter microstructure (radial diusivities) |
| *No data avaiable about FRG1B in this dataset!* | NA | hurdle binary method) |
| *DEFB116* | 0 | Gut microbiota (bacterial taxa |
| *DEFB118* | 1 | Gut microbiota (bacterial taxa |
| *DEFB122* | 0 | hurdle binary method) |
| *HM13* | 1 | hurdle binary method) |
| *HM13-AS1* | 0 | Gut microbiota (bacterial taxa |
| *C20orf173* | NA | Gut microbiota (bacterial taxa |
| *ERGIC3* | 2 | Gut microbiota (bacterial taxa |
| *FER1L4* | 1 | hurdle binary method) |
| *CPNE1* | 2 | hurdle binary method) |
| *PHF20* | 2 | Gut microbiota (bacterial taxa |
| *SCAND1* | 1 | Gut microbiota (bacterial taxa |
| *KIAA1755* | 1 | rank normal transformation method) |
| *BPI* | 0 | Gut microbiota (bacterial taxa |
| *LBP* | 1 | hurdle binary method) |
| *SNHG17* | 0 | Gut microbiota (bacterial taxa |
| *ARHGAP40* | 0 | Gut microbiota (bacterial taxa |
| *SLC32A1* | 0 | Gut microbiota (bacterial taxa |
| *ACTR5* | 2 | hurdle binary method) |
| *PPP1R16B* | 1 | Gut microbiota (bacterial taxa |
| *MSN* | 3 | hurdle binary method) |
| *No data avaiable about RP6-159A1.3 in this dataset!* | NA | Gut microbiota (bacterial taxa |
| *VSIG4* | 0 | hurdle binary method) |
| *No data avaiable about RP11-30M20.1 in this dataset!* | NA | Gut microbiota (bacterial taxa |
| *HEPH* | 2 | hurdle binary method) |
| *No data avaiable about RP11-747I9.1 in this dataset!* | NA | hurdle binary method) |
| *OPHN1* | 2 | Gut microbiota (bacterial taxa |
| *OGT* | 2 | Gut microbiota (bacterial taxa |
| *NHSL2* | 0 | hurdle binary method) |
| *PLS3* | 0 | hurdle binary method) |
| *AGTR2* | 0 | Cognitive impairment in depression |
| *SOWAHD* | 0 | Eosinophil percentage of white cells |
| *RPL39* | 3 | Eosinophil percentage of white cells |
| *TMEM255A* | 0 | Eosinophil percentage of white cells |
| *ATP1B4* | 0 | Eosinophil percentage of white cells |
| *PIGX* | 0 | Eosinophil percentage of white cells |
| *PAK2* | 2 | Eosinophil percentage of white cells |
| *No data avaiable about U4 in this dataset!* | NA | Eosinophil percentage of white cells |
| *RNU6-42P* | 0 | Eosinophil percentage of white cells |
| *SENP5* | 2 | Eosinophil percentage of white cells |
| *DLG1* | 2 | Eosinophil percentage of white cells |
| *BDH1* | 1 | Hematocrit |
| *UGT2B7* | 0 | Hematocrit |
| *NIPAL1* | 0 | Hematocrit |
| *TXK* | 1 | Hematocrit |
| *SLAIN2* | 1 | Waist-to-hip ratio adjusted for BMI |
| *ZAR1* | 0 | Waist-to-hip ratio adjusted for BMI |
| *DCUN1D4* | 2 | Waist-to-hip ratio adjusted for BMI |
| *UNKL* | 1 | Waist-to-hip ratio adjusted for BMI |
| *CLCN7* | 2 | White matter microstructure (radial diusivities) |
| *PTX4* | 0 | White matter microstructure (radial diusivities) |
| *MAPK8IP3* | 1 | White matter microstructure (radial diusivities) |
| *TEC* | 3 | White matter microstructure (radial diusivities) |
| *FRYL* | 1 | Fasting plasma glucose |
| *OCIAD1* | 1 | Fasting plasma glucose |
| *CWH43* | 0 | Fasting plasma glucose |
| *No data avaiable about RP11-707A18.1 in this dataset!* | NA | Fasting plasma glucose |
| *EPHA5* | 2 | Fasting plasma glucose |
| *HTN1* | 1 | Hematocrit |
| *MUC7* | 0 | Hematocrit |
| *ENAM* | 0 | Hemoglobin concentration |
| *UTP3* | 1 | Hemoglobin concentration |
| *No data avaiable about RP11-777N19.1 in this dataset!* | NA | Hemoglobin concentration |
| *MIR297* | 0 | Hemoglobin concentration |
| *LYPLA1P2* | 0 | Hemoglobin concentration |
| *NDST4* | 3 | Hemoglobin concentration |
| *TRAM1L1* | 1 | Hemoglobin concentration |
| *No data avaiable about RP11-97E7.1 in this dataset!* | NA | Deliberate self-harm |
| *GALNTL6* | 1 | Deliberate self-harm |
| *GALNT7* | 1 | Deliberate self-harm |
| *RPL21P110* | 0 | Deliberate self-harm |
| *BORA* | 1 | Deliberate self-harm |
| *PIBF1* | 1 | Deliberate self-harm |
| *KLF5* | 1 | Deliberate self-harm |
| *RNY1P8* | 0 | Deliberate self-harm |
| *LINC00393* | 0 | Deliberate self-harm |
| *KLF12* | 0 | Deliberate self-harm |
| *LINC00381* | 1 | Deliberate self-harm |
| *SAV1* | 1 | Deliberate self-harm |
| *NIN* | 1 | White matter microstructure (axial diusivities) |
| *PYGL* | 2 | White matter microstructure (axial diusivities) |
| *ABHD12B* | 0 | White matter microstructure (axial diusivities) |
| *TRIM9* | 1 | White matter microstructure (axial diusivities) |
| *TMX1* | 1 | White matter microstructure (axial diusivities) |
| *FRMD6-AS2* | 1 | Waist-to-hip ratio adjusted for BMI |
| *FRMD6-AS2* | 1 | Left ventricular ejection fraction |
| *GNG2* | 2 | Left ventricular ejection fraction |
| *NID2* | 3 | Emotional recognition |
| *TXNDC16* | 1 | Emotional recognition |
| *TTLL5* | 2 | Emotional recognition |
| *IFT43* | 2 | Emotional recognition |
| *GPATCH2L* | 1 | Emotional recognition |
| *ESRRB* | 3 | Emotional recognition |
| *No data avaiable about C14ORF166B in this dataset!* | NA | Emotional recognition |
| *IRF2BPL* | 1 | Emotional recognition |
| *ZDHHC22* | 1 | Cleft lip with or without cleft palate |
| *TMEM63C* | 1 | Asthma |
| *SAMD15* | 0 | Asthma |
| *NOXRED1* | 0 | Asthma |
| *AKT1* | 1 | Asthma |
| *PLD4* | 1 | Asthma |
| *AHNAK2* | 0 | Asthma |
| *CDCA4* | 3 | Asthma |
| *BRF1* | 0 | Asthma |
| *MTA1* | 3 | Asthma |
| *No data avaiable about RP11-279F6.3 in this dataset!* | NA | Asthma |
| *No data avaiable about AC009677.1 in this dataset!* | NA | Asthma |
| *PLA2G15* | 0 | Asthma |
| *SLC7A6* | 1 | Asthma |
| *SLC7A6OS* | 0 | Intelligence |
| *PRMT7* | 0 | Intelligence |
| *SMPD3* | 0 | Intelligence |
| *ZFP90* | 3 | Intelligence |
| *SIGLEC12* | 0 | Intelligence |
| *SIGLEC6* | 1 | Intelligence |
| *SIGLEC5* | 1 | Hematocrit |
| *SIGLEC14* | 0 | Hematocrit |
| *ENPP6* | 2 | Hematocrit |
| *IRF2BP2* | 0 | Hematocrit |
| *RPL19P11* | 0 | Hematocrit |
| *No data avaiable about RP11-152K4.2 in this dataset!* | NA | Hematocrit |
| *DROSHA* | 1 | Hematocrit |
| *MTMR12* | 1 | Hematocrit |
| *ZFR* | 1 | Eosinophil counts |
| *PDZD2* | 2 | Eosinophil counts |
| *SUB1* | 1 | Eosinophil counts |
| *NPR3* | 3 | Eosinophil counts |
| *No data avaiable about CTD-2066L21.3 in this dataset!* | NA | Obese vs. thin |
| *GOLPH3* | 1 | Obese vs. thin |
| *TTC23L* | 1 | Obese vs. thin |
| *DNAJC21* | 1 | Obese vs. thin |
| *AGXT2* | 0 | Obesity (extreme) |
| *PRLR* | 3 | Obesity (extreme) |
| *SPEF2* | 1 | Waist-to-hip ratio adjusted for BMI |
| *No data avaiable about CTD-2113L7.1 in this dataset!* | NA | Waist-to-hip ratio adjusted for BMI |
| *IL7R* | 1 | Waist-to-hip ratio adjusted for BMI |
| *CAPSL* | 1 | Waist-to-hip ratio adjusted for BMI |
| *UGT3A1* | 0 | Waist-to-hip ratio adjusted for BMI |
| *UGT3A2* | 1 | Waist-to-hip ratio adjusted for BMI |
| *LMBRD2* | 1 | Waist-to-hip ratio adjusted for BMI |
| *RN7SKP229* | 0 | Waist-to-hip ratio adjusted for BMI |
| *RANBP3L* | 0 | General cognitive ability |
| *RNA5SP181* | 0 | General cognitive ability |
| *No data avaiable about CTD-2353F22.1 in this dataset!* | NA | General cognitive ability |
| *NIPBL* | 2 | General cognitive ability |
| *C5orf42* | NA | General cognitive ability |
| *CARD6* | 1 | General cognitive ability |
| *CDC7* | 4 | General cognitive ability |
| *No data avaiable about CTC-467M3.1 in this dataset!* | NA | Hematocrit |
| *MEF2C-AS1* | 1 | Hematocrit |
| *MIR3660* | 1 | Hematocrit |
| *No data avaiable about RP11-61G23.1 in this dataset!* | NA | Hematocrit |
| *POLR3G* | 1 | Hematocrit |
| *LYSMD3* | 0 | Autoimmune thyroid disease |
| *No data avaiable about GPR98 in this dataset!* | NA | Autoimmune thyroid disease |
| *No data avaiable about CTD-2266L18.1 in this dataset!* | NA | Autoimmune thyroid disease |
| *SEC61G* | 4 | Autoimmune thyroid disease |
| *PLCXD3* | 1 | Autoimmune thyroid disease |
| *OXCT1* | 0 | Autoimmune thyroid disease |
| *RN7SL383P* | 0 | Autoimmune thyroid disease |
| *MRPS30* | 2 | Autoimmune thyroid disease |
| *HCN1* | 0 | Autoimmune thyroid disease |
| *No data avaiable about CTD-2203A3.1 in this dataset!* | NA | Autoimmune thyroid disease |
| *EMB* | 1 | Autoimmune thyroid disease |
| *No data avaiable about CTD-2288O8.1 in this dataset!* | NA | Autoimmune thyroid disease |
| *ITGA1* | 3 | Autoimmune thyroid disease |
| *PELO* | 0 | Autoimmune thyroid disease |
| *PARP8* | 1 | Autoimmune thyroid disease |
| *ITGA2* | 2 | Autoimmune thyroid disease |
| *ISL1* | 2 | Autoimmune thyroid disease |
| *MOCS2* | 1 | Autoimmune thyroid disease |
| *No data avaiable about CTD-2366F13.1 in this dataset!* | NA | Autoimmune thyroid disease |
| *No data avaiable about AC022431.2 in this dataset!* | NA | Autoimmune thyroid disease |
| *ADAMTS6* | 1 | Gut microbiota (bacterial taxa |
| *CCDC125* | 0 | hurdle binary method) |
| *No data avaiable about RP11-60A8.1 in this dataset!* | NA | Gut microbiota (bacterial taxa |
| *FOXD1* | 0 | Gut microbiota (bacterial taxa |
| *PDE8B* | 1 | Gut microbiota (bacterial taxa |
| *WDR41* | 1 | hurdle binary method) |
| *TBCA* | 1 | hurdle binary method) |
| *AP3B1* | 3 | Gut microbiota (bacterial taxa |
| *SCAMP1* | 0 | hurdle binary method) |
| *LHFPL2* | 2 | Gut microbiota (bacterial taxa |
| *MIR4280* | 0 | Gut microbiota (bacterial taxa |
| *RASA1* | 2 | hurdle binary method) |
| *CCNH* | 1 | Gut microbiota (bacterial taxa |
| *TMEM161B* | 0 | hurdle binary method) |
| *TMEM161B-AS1* | 0 | Snoring |
| *LINC00461* | 1 | Snoring |
| *SNCAIP* | 1 | Snoring |
| *No data avaiable about RP11-166A12.1 in this dataset!* | NA | Snoring |
| *SNX2* | 0 | Snoring |
| *No data avaiable about RP11-434D11.4 in this dataset!* | NA | Snoring |
| *LMNB1* | 2 | Snoring |
| *C5orf63* | NA | Snoring |
| *HNRNPAB* | 1 | Snoring |
| *RNF111* | 3 | Snoring |
| *MYO1E* | 1 | Snoring |
| *PRRC1* | 1 | Snoring |
| *CTXN3* | 1 | Hematocrit |
| *No data avaiable about CTC-228N24.1 in this dataset!* | NA | Hematocrit |
| *No data avaiable about CTC-228N24.3 in this dataset!* | NA | Hematocrit |
| *SLC12A2* | 2 | Hematocrit |
| *FBN2* | 1 | Hematocrit |
| *CLK4* | 3 | Hematocrit |
| *ZNF354A* | 1 | Hematocrit |
| *MSX2* | 1 | Hematocrit |
| *AACSP1* | 0 | Hematocrit |
| *ZNF354B* | 1 | Hematocrit |
| *No data avaiable about RP3-416J7.4 in this dataset!* | NA | Hematocrit |
| *DUSP22* | 1 | Hematocrit |
| *IRF4* | 2 | Blood protein levels |
| *EXOC2* | 1 | Blood protein levels |
| *MYLK4* | 1 | Blood protein levels |
| *No data avaiable about RP11-532F6.5 in this dataset!* | NA | Blood protein levels |
| *SERPINB9* | 1 | Blood protein levels |
| *FOXQ1* | 3 | Blood protein levels |
| *FOXF2* | 2 | Blood protein levels |
| *No data avaiable about RP11-157J24.1 in this dataset!* | NA | Blood protein levels |
| *FOXC1* | 1 | Blood protein levels |
| *GMDS-AS1* | 1 | Blood protein levels |
| *NQO2* | 0 | Blood protein levels |
| *RIPK1* | 3 | Blood protein levels |
| *MYBPHL* | 1 | Blood protein levels |
| *TUBB2A* | 3 | Adolescent idiopathic scoliosis |
| *SLC22A23* | 1 | Adolescent idiopathic scoliosis |
| *FAM50B* | 0 | Adolescent idiopathic scoliosis |
| *No data avaiable about RP11-274H24.1 in this dataset!* | NA | Adolescent idiopathic scoliosis |
| *No data avaiable about RP11-420L9.4 in this dataset!* | NA | Adolescent idiopathic scoliosis |
| *ECI2* | 0 | Adolescent idiopathic scoliosis |
| *RPS3AP23* | 0 | Adolescent idiopathic scoliosis |
| *VTA1* | 1 | Adolescent idiopathic scoliosis |
| *No data avaiable about GPR126 in this dataset!* | NA | Adolescent idiopathic scoliosis |
| *No data avaiable about RP1-67K17.3 in this dataset!* | NA | Adolescent idiopathic scoliosis |
| *HIVEP2* | 0 | Gut microbiota (bacterial taxa |
| *No data avaiable about RP11-439L18.2 in this dataset!* | NA | rank normal transformation method) |
| *No data avaiable about RP11-439L18.3 in this dataset!* | NA | Gut microbiota (bacterial taxa |
| *AIG1* | 1 | rank normal transformation method) |
| *HFE* | 2 | Gut microbiota (bacterial taxa |
| *HIST1H4C* | 1 | Gut microbiota (bacterial taxa |
| *HIST1H1T* | 0 | rank normal transformation method) |
| *HIST1H2BC* | 3 | Gut microbiota (bacterial taxa |
| *HIST1H2AC* | 2 | Gut microbiota (bacterial taxa |
| *HIST1H1E* | 2 | rank normal transformation method) |
| *HIST1H2BD* | 1 | Gut microbiota (bacterial taxa |
| *HIST1H2BE* | 3 | rank normal transformation method) |
| *HIST1H4D* | 1 | rank normal transformation method) |
| *HIST1H3D* | 2 | Gut microbiota (bacterial taxa |
| *HIST1H2BF* | 3 | Gut microbiota (bacterial taxa |
| *SLC25A27* | 3 | Gut microbiota (bacterial taxa |
| *PLA2G7* | 1 | rank normal transformation method) |
| *No data avaiable about GPR116 in this dataset!* | NA | rank normal transformation method) |
| *No data avaiable about GPR110 in this dataset!* | NA | rank normal transformation method) |
| *TNFRSF21* | 3 | Gut microbiota (bacterial taxa |
| *No data avaiable about GPR111 in this dataset!* | NA | rank normal transformation method) |
| *No data avaiable about AC007652.1 in this dataset!* | NA | Gut microbiota (bacterial taxa |
| *HERPUD2* | 0 | rank normal transformation method) |
| *No data avaiable about AC083864.3 in this dataset!* | NA | rank normal transformation method) |
| *PP13004* | 0 | Gut microbiota (bacterial taxa |
| *No data avaiable about RP11-377H23.1 in this dataset!* | NA | rank normal transformation method) |
| *EEPD1* | 0 | rank normal transformation method) |
| *ANLN* | 2 | Gut microbiota (bacterial taxa |
| *PSORS1C2* | 1 | rank normal transformation method) |
| *CCHCR1* | 1 | rank normal transformation method) |
| *TCF19* | 0 | Gut microbiota (bacterial taxa |
| *POU5F1* | 1 | rank normal transformation method) |
| *No data avaiable about XXBAC-BPG181B23.7 in this dataset!* | NA | Gut microbiota (bacterial taxa |
| *HCP5* | 0 | rank normal transformation method) |
| *MICA* | 1 | Gut microbiota (bacterial taxa |
| *MICB* | 1 | rank normal transformation method) |
| *No data avaiable about XXBAC-BPG16N22.5 in this dataset!* | NA | Gut microbiota (bacterial taxa |
| *PPIAP9* | 0 | rank normal transformation method) |
| *MCCD1* | 0 | rank normal transformation method) |
| *DDX39B* | 0 | Gut microbiota (bacterial taxa |
| *ATP6V1G2* | 1 | Gut microbiota (bacterial taxa |
| *NFKBIL1* | 0 | Gut microbiota (bacterial taxa |
| *LTA* | 0 | rank normal transformation method) |
| *TNFSF13B* | 4 | rank normal transformation method) |
| *LST1* | 2 | Gut microbiota (bacterial taxa |
| *NCR3* | 0 | rank normal transformation method) |
| *UQCRHP1* | 0 | Gut microbiota (bacterial taxa |
| *AIF1* | 2 | rank normal transformation method) |
| *VNN3* | 0 | Gut microbiota (bacterial taxa |
| *PSORS1C3* | 0 | rank normal transformation method) |
| *No data avaiable about XXBAC-BPG299F13.17 in this dataset!* | NA | rank normal transformation method) |
| *HCG27* | 1 | White matter microstructure (radial diusivities) |
| *HLA-C* | 3 | White matter microstructure (radial diusivities) |
| *USP8P1* | 0 | White matter microstructure (radial diusivities) |
| *RPL3P2* | 0 | White matter microstructure (radial diusivities) |
| *WASF5P* | 0 | White matter microstructure (radial diusivities) |
| *No data avaiable about XXBAC-BPG248L24.13 in this dataset!* | NA | Type 2 diabetes |
| *No data avaiable about XXBAC-BPG248L24.10 in this dataset!* | NA | Type 2 diabetes |
| *HLA-B* | 2 | Type 2 diabetes |
| *No data avaiable about XXBAC-BPG248L24.12 in this dataset!* | NA | Type 2 diabetes |
| *DHFRP2* | 0 | Type 2 diabetes |
| *RNU6-283P* | 0 | Type 2 diabetes |
| *FGFR3P1* | 0 | Type 2 diabetes |
| *HLA-S* | 0 | Type 2 diabetes |
| *PRRC2A* | 0 | Type 2 diabetes |
| *SNRPC* | 1 | Type 2 diabetes |
| *NECAB2* | 1 | Type 2 diabetes |
| *SLC38A8* | 1 | General cognitive ability |
| *UHRF1BP1* | 1 | General cognitive ability |
| *TFAP2B* | 0 | General cognitive ability |
| *FTH1P5* | 0 | General cognitive ability |
| *No data avaiable about RP3-335N17.2 in this dataset!* | NA | General cognitive ability |
| *PKHD1* | 1 | General cognitive ability |
| *IL17A* | 1 | General cognitive ability |
| *IL17F* | 0 | General cognitive ability |
| *PAQR8* | 2 | General cognitive ability |
| *EFHC1* | 1 | Lung cancer (survival time) in never smokers |
| *TRAM2* | 0 | Lung cancer (survival time) in never smokers |
| *TRAM2-AS1* | 0 | Lung adenocarcinoma (survival time) in never smokers |
| *TMEM14A* | 1 | Lung adenocarcinoma (survival time) in never smokers |
| *GSTA7P* | 0 | Lung adenocarcinoma (survival time) in never smokers |
| *GSTA2* | 2 | Adolescent idiopathic scoliosis |
| *No data avaiable about RP1-152L7.1 in this dataset!* | NA | Adolescent idiopathic scoliosis |
| *GSTA1* | 1 | Adolescent idiopathic scoliosis |
| *No data avaiable about RP11-392A23.4 in this dataset!* | NA | Adolescent idiopathic scoliosis |
| *GSTA4* | 1 | Adolescent idiopathic scoliosis |
| *FYN* | 1 | Adolescent idiopathic scoliosis |
| *LAMA4* | 0 | Adolescent idiopathic scoliosis |
| *RFPL4B* | 0 | Adolescent idiopathic scoliosis |
| *No data avaiable about RP1-159M24.1 in this dataset!* | NA | Adolescent idiopathic scoliosis |
| *SOD2* | 3 | Adolescent idiopathic scoliosis |
| *WTAP* | 2 | Adolescent idiopathic scoliosis |
| *ACAT2* | 2 | Adolescent idiopathic scoliosis |
| *CYP51A1* | 1 | Adolescent idiopathic scoliosis |
| *No data avaiable about CTB-161K23.1 in this dataset!* | NA | Adolescent idiopathic scoliosis |
| *KRIT1* | 0 | Adolescent idiopathic scoliosis |
| *ANKIB1* | 1 | Adolescent idiopathic scoliosis |
| *No data avaiable about AC007566.10 in this dataset!* | NA | Waist-to-hip ratio adjusted for BMI |
| *FAM133B* | 1 | Waist-to-hip ratio adjusted for BMI |
| *CDK6* | 3 | Waist-to-hip ratio adjusted for BMI |
| *No data avaiable about AC002454.1 in this dataset!* | NA | Waist-to-hip ratio adjusted for BMI |
| *RN7SL7P* | 0 | Waist-to-hip ratio adjusted for BMI |
| *FBXL13* | 1 | Waist-to-hip ratio adjusted for BMI |
| *SLC26A5* | 1 | Waist-to-hip ratio adjusted for BMI |
| *FEZF1* | 1 | Waist-to-hip ratio adjusted for BMI |
| *CADPS2* | 2 | Waist-to-hip ratio adjusted for BMI |
| *SLC13A1* | 0 | Waist-to-hip ratio adjusted for BMI |
| *ASB15* | 0 | Waist-to-hip ratio adjusted for BMI |
| *PODXL* | 1 | Nontraumatic osteonecrosis of the femoral head |
| *PLXNA4* | 1 | Nontraumatic osteonecrosis of the femoral head |
| *CCT8L1P* | 0 | Nontraumatic osteonecrosis of the femoral head |
| *XRCC2* | 2 | Nontraumatic osteonecrosis of the femoral head |
| *ACTR3B* | 1 | Nontraumatic osteonecrosis of the femoral head |
| *DPP6* | 3 | White matter microstructure (radial diusivities) |
| *DPYSL2* | 3 | White matter microstructure (radial diusivities) |
| *ADRA1A* | 2 | White matter microstructure (radial diusivities) |
| *MIR548H4* | 0 | White matter microstructure (radial diusivities) |
| *STMN4* | 3 | White matter microstructure (radial diusivities) |
| *TRIM35* | 1 | White matter microstructure (radial diusivities) |
| *PTK2B* | 3 | White matter microstructure (radial diusivities) |
| *CHRNA2* | 2 | White matter microstructure (radial diusivities) |
| *GULOP* | 0 | White matter microstructure (radial diusivities) |
| *RECK* | 1 | Systolic blood pressure x educational attainment (some college) interaction (2df) |
| *HMGB3P24* | 0 | Systolic blood pressure x educational attainment (some college) interaction (2df) |
| *PAX5* | 1 | Systolic blood pressure x educational attainment (graduated college) interaction (2df) |
| *No data avaiable about RP11-791O21.5 in this dataset!* | NA | Mean arterial pressure x educational attainment (some college) interaction (2df) |
| *C9orf47* | NA | Mean arterial pressure x educational attainment (graduated college) interaction (2df) |
| *S1PR3* | 2 | Asthma |
| *SHC3* | 2 | Asthma |
| *SECISBP2* | 3 | Asthma |
| *SEMA4D* | 3 | Asthma |
| *COL27A1* | 2 | Asthma |
| *ORM1* | 0 | Asthma |
| *ORM2* | 1 | Asthma |
| *AKNA* | 3 | Asthma |
| *DFNB31* | 2 | Asthma |
| *ATP6V1G1* | 1 | Asthma |
| *C9orf91* | NA | Asthma |
| *No data avaiable about RP11-402G3.3 in this dataset!* | NA | Asthma |
| *TNFSF15* | 1 | Asthma |
| *TNFSF8* | 0 | Asthma |
| *TNC* | 3 | Asthma |
| *LHX2* | 3 | Asthma |
| *NEK6* | 3 | Asthma |
| *PSMB7* | 3 | Asthma |
| *NR5A1* | 1 | Hematocrit |
| *NR6A1* | 0 | Hematocrit |
| *OLFML2A* | 0 | Hematocrit |
| *LINC00701* | 0 | Hematocrit |
| *PFKP* | 3 | Hematocrit |
| *PITRM1* | 1 | Hematocrit |
| *No data avaiable about RP11-184A2.3 in this dataset!* | NA | Hematocrit |
| *MYO3A* | 0 | Hematocrit |
| *GAD2* | 1 | Hematocrit |
| *APBB1IP* | 2 | Blood protein levels |
| *PDSS1* | 2 | Blood protein levels |
| *ANKRD26* | 1 | Blood protein levels |
| *YME1L1* | 3 | Blood protein levels |
| *MSMB* | 1 | Blood protein levels |
| *NCOA4* | 1 | Blood protein levels |
| *TIMM23* | 2 | White matter microstructure (radial diusivities) |
| *LINC00843* | 0 | White matter microstructure (radial diusivities) |
| *USP54* | 1 | White matter microstructure (radial diusivities) |
| *SYNPO2L* | 1 | White matter microstructure (radial diusivities) |
| *AGAP5* | 0 | White matter microstructure (radial diusivities) |
| *SEC24C* | 2 | White matter microstructure (radial diusivities) |
| *FUT11* | 3 | White matter microstructure (radial diusivities) |
| *NIP7P1* | 0 | Drug-induced liver injury (flucloxacillin) |
| *MYOF* | 1 | Hematocrit |
| *CEP55* | 1 | Hematocrit |
| *FFAR4* | 1 | Hematocrit |
| *RBP4* | 1 | Hematocrit |
| *FRA10AC1* | 1 | Hematocrit |
| *LGI1* | 1 | Hematocrit |
| *SLC35G1* | 1 | Hematocrit |
| *PIPSL* | 0 | Hematocrit |
| *PLCE1* | 3 | Hematocrit |
| *CWF19L1* | 1 | Hematocrit |
| *PKD2L1* | 1 | Depressive symptoms x herpes simplex 1 infection interaction |
| *No data avaiable about RP11-34D15.2 in this dataset!* | NA | Depressive symptoms x herpes simplex 1 infection interaction |
| *SCD* | 0 | Depression x herpes simplex 1 infection interaction |
| *HIF1AN* | 1 | Sweet taste preference |
| *PAX2* | 1 | Sweet taste preference |
| *No data avaiable about FAM178A in this dataset!* | NA | Sweet taste preference |
| *No data avaiable about NEURL in this dataset!* | NA | Cardiorespiratory fitness (800m run time) |
| *SH3PXD2A* | 1 | Cardiorespiratory fitness (800m run time) |
| *No data avaiable about RP11-541N10.3 in this dataset!* | NA | Hematocrit |
| *OR4C13* | 0 | Hematocrit |
| *OR4C12* | 0 | Hematocrit |
| *No data avaiable about RP11-347H15.4 in this dataset!* | NA | Hematocrit |
| *FAM111A* | 0 | Hematocrit |
| *DTX4* | 2 | Hematocrit |
| *RPL31P28* | 0 | Hematocrit |
| *GCLC* | 3 | Hematocrit |
| *IBTK* | 1 | Hematocrit |
| *PRSS35* | 1 | Hematocrit |
| *SNAP91* | 2 | Hematocrit |
| *YME1L1* | 3 | Hematocrit |
| *SIM1* | 2 | Hematocrit |
| *ASCC3* | 1 | Hematocrit |
| *C6orf203* | NA | Hematocrit |
| *BEND3* | 0 | Hematocrit |
| *PDSS2* | 0 | Hematocrit |
| *SOBP* | 0 | Hematocrit |
| *SCML4* | 3 | Hematocrit |
| *SEC63* | 1 | Hematocrit |
| *OSTM1* | 2 | Hematocrit |
| *LACE1* | 3 | Hematocrit |
| *DNAJA1P4* | 0 | Hematocrit |
| *FRK* | 0 | Eosinophil counts |
| *NT5DC1* | 1 | Eosinophil counts |
| *COL10A1* | 0 | Eosinophil counts |
| *TSPYL4* | 0 | Eosinophil counts |
| *DSEL* | 1 | Eosinophil counts |
| *No data avaiable about RP1-155D22.1 in this dataset!* | NA | Eosinophil counts |
| *C6orf118* | NA | Gut microbiota (bacterial taxa |
| *PDE10A* | 2 | hurdle binary method) |
| *No data avaiable about RP11-252P19.1 in this dataset!* | NA | hurdle binary method) |
| *ARG1* | 3 | Gut microbiota (bacterial taxa |
| *ENPP3* | 0 | hurdle binary method) |
| *MIR548H5* | 0 | Gut microbiota (bacterial taxa |
| *ENPP1* | 2 | hurdle binary method) |
| *CTGF* | 2 | hurdle binary method) |
| *No data avaiable about RP11-69I8.3 in this dataset!* | NA | Gut microbiota (bacterial taxa |
| *MOXD1* | 3 | hurdle binary method) |
| *STX7* | 0 | hurdle binary method) |
| *TAAR1* | 1 | Gut microbiota (bacterial taxa |
| *LINC00326* | 0 | hurdle binary method) |
| *EYA4* | 0 | hurdle binary method) |
| *No data avaiable about RP3-323P13.2 in this dataset!* | NA | hurdle binary method) |
| *No data avaiable about RP4-662A9.2 in this dataset!* | NA | hurdle binary method) |
| *TCF21* | 0 | hurdle binary method) |
| *TBPL1* | 0 | Gut microbiota (bacterial taxa |
| *SLC2A12* | 1 | hurdle binary method) |
| *SGK1* | 1 | White matter microstructure (radial diusivities) |
| *FGFR1OP* | 2 | White matter microstructure (radial diusivities) |
| *CCR6* | 3 | White matter microstructure (radial diusivities) |
| *FAM20C* | 0 | World class endurance athleticism |
| *CHST12* | 1 | Migraine and/or diastolic blood pressure |
| *PDGFA* | 2 | Migraine and/or diastolic blood pressure |
| *No data avaiable about AC074389.1 in this dataset!* | NA | Migraine and/or systolic blood pressure |
| *SNX8* | 2 | Migraine and/or diastolic blood pressure |
| *SUN1* | 1 | Migraine and/or pulse pressure |
| *COX19* | 1 | Migraine and/or systolic blood pressure |
| *GRIFIN* | 0 | Migraine and/or systolic blood pressure |
| *TTYH3* | 2 | Migraine and/or systolic blood pressure |
| *ADAP1* | 1 | Migraine and/or systolic blood pressure |
| *CYP2W1* | 1 | Migraine and/or systolic blood pressure |
| *C7orf50* | NA | Migraine and/or pulse pressure |
| *IQCE* | 2 | Eosinophil counts |
| *BZW2* | 2 | Eosinophil percentage of white cells |
| *MPP6* | 3 | Eosinophil percentage of white cells |
| *DFNA5* | 1 | Eosinophil percentage of white cells |
| *No data avaiable about RP1-170O19.14 in this dataset!* | NA | Eosinophil percentage of white cells |
| *CYCS* | 2 | Eosinophil percentage of white cells |
| *C7orf31* | NA | Blood protein levels |
| *NPVF* | 2 | Blood protein levels |
| *No data avaiable about AC091705.1 in this dataset!* | NA | Adolescent idiopathic scoliosis |
| *No data avaiable about CTD-2227E11.1 in this dataset!* | NA | Adolescent idiopathic scoliosis |
| *MIR148A* | 1 | Adolescent idiopathic scoliosis |
| *NFE2L3* | 1 | Adolescent idiopathic scoliosis |
| *HOXA10* | 1 | rank normal transformation method) |
| *HOXA11* | 0 | Gut microbiota (bacterial taxa |
| *SH2B2* | 1 | rank normal transformation method) |
| *SMOC1* | 4 | Gut microbiota (bacterial taxa |
| *AHCYL2* | 1 | rank normal transformation method) |
| *SMKR1* | 0 | Gut microbiota (bacterial taxa |
| *No data avaiable about RP11-448A19.1 in this dataset!* | NA | rank normal transformation method) |
| *NRF1* | 3 | rank normal transformation method) |
| *UBE2H* | 1 | Gut microbiota (bacterial taxa |
| *No data avaiable about RP11-306G20.1 in this dataset!* | NA | Gut microbiota (bacterial taxa |
| *ZC3HC1* | 2 | Gut microbiota (bacterial taxa |
| *CEP41* | 1 | Gut microbiota (bacterial taxa |
| *RNU6-1010P* | 0 | rank normal transformation method) |
| *MKLN1* | 2 | rank normal transformation method) |
| *ATP6V0A4* | 1 | rank normal transformation method) |
| *KIAA1549* | 1 | Gut microbiota (bacterial taxa |
| *ZC3HAV1L* | 1 | rank normal transformation method) |
| *ZC3HAV1L* | 1 | Gut microbiota (bacterial taxa |
| *TTC26* | 0 | Gut microbiota (bacterial taxa |
| *C7orf55-LUC7L2* | NA | rank normal transformation method) |
| *HIPK2* | 1 | Gut microbiota (bacterial taxa |
| *TBXAS1* | 2 | rank normal transformation method) |
| *PARP12* | 1 | rank normal transformation method) |
| *RAB19* | 0 | rank normal transformation method) |
| *MKRN1* | 2 | rank normal transformation method) |
| *DENND2A* | 0 | Migraine without aura and/or pulse pressure |
| *BRAF* | 2 | Stroke |
| *No data avaiable about RP4-813F11.3 in this dataset!* | NA | Stroke |
| *TMEM178B* | 0 | Stroke |
| *MGAM* | 0 | Stroke |
| *CLEC5A* | 1 | Stroke |
| *TAS2R38* | 0 | Stroke |
| *CHCHD3* | 2 | Stroke |
| *EXOC4* | 2 | Stroke |
| *LRGUK* | 2 | Eosinophil percentage of white cells |
| *SLC35B4* | 3 | White matter microstructure (mode of anisotropy) |
| *SLC3A2* | 0 | Blood protein levels |
| *CHRM1* | 1 | Response to esketamine (responder status) in treatment resistant depression |
| *SLC22A6* | 1 | Response to esketamine (responder status) in treatment resistant depression |
| *SLC22A8* | 1 | Response to esketamine (responder status) in treatment resistant depression |
| *SLC22A10* | 1 | Response to esketamine in treatment resistant depression |
| *AKR1B1* | 2 | Response to esketamine in treatment resistant depression |
| *AKR1B10* | 2 | Response to esketamine in treatment resistant depression |
| *AKR1B15* | 1 | Response to esketamine in treatment resistant depression |
| *BPGM* | 3 | Response to esketamine in treatment resistant depression |
| *No data avaiable about AC009276.4 in this dataset!* | NA | Response to esketamine in treatment resistant depression |
| *CALD1* | 0 | Response to esketamine in treatment resistant depression |
| *AGBL3* | 0 | Response to esketamine in treatment resistant depression |
| *WDR91* | 0 | Response to esketamine in treatment resistant depression |
| *CNOT4* | 2 | Response to esketamine in treatment resistant depression |
| *NUP205* | 1 | Total PHF-tau (SNP x SNP interaction) |
| *C7orf73* | NA | Total PHF-tau (SNP x SNP interaction) |
| *SLC13A4* | 2 | Anti-Epstein-Barr virus nuclear antigen (EBNA) IgG levels |
| *FAM180A* | 0 | Anti-Epstein-Barr virus nuclear antigen (EBNA) IgG levels |
| *MTPN* | 0 | Coffee consumption |
| *No data avaiable about AC009784.3 in this dataset!* | NA | Coffee consumption |
| *PSMC1P3* | 0 | Coffee consumption |
| *RPTN* | 0 | Coffee consumption |
| *ZNF783* | 1 | Response to esketamine (remission) in treatment resistant depression |
| *No data avaiable about AC004941.3 in this dataset!* | NA | Response to esketamine (remission) in treatment resistant depression |
| *ZNF746* | 1 | Type 2 diabetes |
| *No data avaiable about ZNF767 in this dataset!* | NA | Type 2 diabetes |
| *KRBA1* | 1 | Type 2 diabetes |
| *ZNF467* | 1 | Type 2 diabetes |
| *DLGAP2* | 2 | Type 2 diabetes |
| *CLN8* | 3 | Type 2 diabetes |
| *ARHGEF10* | 3 | Type 2 diabetes |
| *KBTBD11* | 1 | Type 2 diabetes |
| *MYOM2* | 2 | Anorectal malformation |
| *CSMD1* | 2 | Anorectal malformation |
| *DEFA4* | 1 | Anorectal malformation |
| *DEFA10P* | 0 | Hematocrit |
| *DEFA10P* | 0 | Hematocrit |
| *DEFA1B* | 0 | Hematocrit |
| *DEFA11P* | 1 | Hematocrit |
| *DEFA7P* | 0 | Hematocrit |
| *DEFA5* | 0 | Lymphocyte counts |
| *VPS37A* | 0 | Lymphocyte counts |
| *MTMR7* | 1 | Lymphocyte counts |
| *ADAM24P* | 0 | Lymphocyte counts |
| *SLC7A2* | 2 | Lymphocyte counts |
| *PDGFRL* | 1 | Lymphocyte counts |
| *MTUS1* | 2 | Lymphocyte counts |
| *FGL1* | 1 | Lymphocyte counts |
| *PCM1* | 2 | Lymphocyte counts |
| *ASAH1* | 2 | Lymphocyte counts |
| *NAT1* | 2 | Hematocrit |
| *GNAT2* | 2 | Total PHF-tau (SNP x SNP interaction) |
| *C8orf4* | NA | Total PHF-tau (SNP x SNP interaction) |
| *ZMAT4* | 2 | Total PHF-tau (SNP x SNP interaction) |
| *RNU6-356P* | 0 | Total PHF-tau (SNP x SNP interaction) |
| *SFRP1* | 1 | Total PHF-tau (SNP x SNP interaction) |
| *GOLGA7* | 2 | Total PHF-tau (SNP x SNP interaction) |
| *No data avaiable about RP11-360L9.4 in this dataset!* | NA | Total PHF-tau (SNP x SNP interaction) |
| *NKX6-3* | 0 | Total PHF-tau (SNP x SNP interaction) |
| *ANK1* | 2 | Type 2 diabetes |
| *No data avaiable about RP11-930P14.1 in this dataset!* | NA | Type 2 diabetes |
| *KAT6A* | 0 | Type 2 diabetes |
| *AP3M2* | 2 | Myocardial infarction |
| *PLAT* | 2 | Myocardial infarction |
| *POLB* | 3 | Myocardial infarction |
| *SLC20A2* | 1 | Myocardial infarction |
| *SMIM19* | 0 | Myocardial infarction |
| *CEBPD* | 1 | Myocardial infarction |
| *PRKDC* | 2 | Myocardial infarction |
| *MCM4* | 1 | Myocardial infarction |
| *RNU6-295P* | 0 | Myocardial infarction |
| *EFCAB1* | 2 | Myocardial infarction |
| *SNAI2* | 1 | Myocardial infarction |
| *No data avaiable about RP11-10H3.1 in this dataset!* | NA | Myocardial infarction |
| *NKAIN3* | 0 | Herpes simplex virus 2 mgG-1 antibody levels |
| *GGH* | 0 | Chlamydia trachomatis PorB antibody levels |
| *TTPA* | 2 | Anti-Epstein-Barr virus IgG seropositivity |
| *YTHDF3* | 2 | Merkel cell polyomavirus VP1 antibody levels |
| *No data avaiable about RP11-32K4.1 in this dataset!* | NA | Toxoplasma gondii sag1 antibody levels |
| *PGM5* | 2 | Anti-varicella zoster virus IgG seropositivity |
| *No data avaiable about RP11-274B18.4 in this dataset!* | NA | Epstein-Barr virus ZEBRA antibody levels |
| *PIP5K1B* | 2 | Anti-herpes simplex virus 6 IE1A IgG seropositivity |
| *FAM122A* | 1 | Herpes simplex virus 6 IE1B antibody levels |
| *PRKACG* | 1 | Helicobacter pylori OMP antibody levels |
| *SFXN5* | 1 | Chlamydia trachomatis PorB antibody levels |
| *TJP2* | 2 | Chlamydia trachomatis PorB antibody levels |
| *FAM189A2* | 1 | Toxoplasma gondii sag1 antibody levels |
| *C9orf135* | NA | Varicella zoster virus glycoproteins E and I antibody levels |
| *MAMDC2* | 3 | Varicella zoster virus glycoproteins E and I antibody levels |
| *SMC5* | 0 | Blood protein levels |
| *KLF9* | 1 | Blood protein levels |
| *TRPM3* | 2 | Blood protein levels |
| *TMEM251* | 0 | Blood protein levels |
| *SPTLC1* | 2 | Blood protein levels |
| *IARS* | 1 | Blood protein levels |
| *CENPP* | 2 | Blood protein levels |
| *IPPK* | 0 | Blood protein levels |
| *DAB2IP* | 3 | Blood protein levels |
| *TTLL11* | 1 | Blood protein levels |
| *NDUFA8* | 0 | Blood protein levels |
| *PTGS1* | 3 | Male-pattern baldness |
| *OR1B1* | 0 | Gut microbiota relative abundance (Eubacterium belonging to family Erysipelotrichaceae) |
| *OR1L1* | 1 | Gut microbiota relative abundance (unclassified genus belonging to family Erysipelotrichaceae) |
| *OR1L4* | 0 | Gut microbiota relative abundance (unclassified genus belonging to family Erysipelotrichaceae) |
| *PDCL* | 2 | Gut microbiota relative abundance (unclassified genus belonging to family Erysipelotrichaceae) |
| *RABGAP1* | 3 | Gut microbiota relative abundance (Sutterella) |
| *SCAI* | 1 | Type 2 diabetes |
| *PPP6C* | 2 | Type 2 diabetes |
| *GAPVD1* | 3 | Type 2 diabetes |
| *MAPKAP1* | 2 | Type 2 diabetes |
| *PBX3* | 3 | Type 2 diabetes |
| *MVB12B* | 1 | Type 2 diabetes |
| *LMX1B* | 1 | Type 2 diabetes |
| *ZBTB43* | 1 | Type 2 diabetes |
| *RALGPS1* | 1 | Type 2 diabetes |
| *ANGPTL2* | 1 | Type 2 diabetes |
| *GARNL3* | 2 | Type 2 diabetes |
| *SLC2A8* | 2 | Type 2 diabetes |
| *LRSAM1* | 2 | Type 2 diabetes |
| *FAM129B* | 1 | Type 2 diabetes |
| *AKR1C4* | 1 | Type 2 diabetes |
| *AKR1C7P* | 0 | Type 2 diabetes |
| *UCN3* | 2 | Lymphocyte counts |
| *TUBAL3* | 1 | Lymphocyte counts |
| *ASB13* | 1 | Lymphocyte counts |
| *FAM208B* | 1 | Lymphocyte counts |
| *GDI2* | 0 | Lymphocyte counts |
| *ANKRD16* | 0 | Hemoglobin |
| *MCM10* | 1 | Hemoglobin |
| *UCMA* | 1 | Hemoglobin |
| *No data avaiable about AL138764.1 in this dataset!* | NA | Hemoglobin |
| *SEPHS1* | 1 | Hemoglobin |
| *BEND7* | 0 | Hemoglobin |
| *PRPF18* | 1 | Hemoglobin |
| *FRMD4A* | 1 | Hemoglobin |
| *No data avaiable about AL157392.1 in this dataset!* | NA | Hemoglobin |
| *CACNB2* | 1 | Hemoglobin |
| *BMPR1A* | 2 | Hemoglobin |
| *FAM35A* | 0 | Hemoglobin |
| *NOC3L* | 0 | Hemoglobin |
| *TBC1D12* | 0 | Hemoglobin |
| *HELLS* | 0 | Hemoglobin |
| *CYP2C18* | 0 | Lymphocyte counts |
| *CYP2C19* | 1 | Lymphocyte counts |
| *CYP2C9* | 1 | Lymphocyte counts |
| *CYP2C8* | 1 | Lymphocyte counts |
| *MSRA* | 0 | Hypertension |
| *No data avaiable about RP11-981G7.2 in this dataset!* | NA | Total PHF-tau (SNP x SNP interaction) |
| *GDA* | 2 | Coronary artery disease |
| *ZFAND5* | 0 | Coronary artery disease |
| *ALDH1A1* | 2 | Coronary artery disease |
| *ANXA1* | 2 | Coronary artery disease |
| *No data avaiable about RP11-401G5.1 in this dataset!* | NA | Coronary artery disease |
| *AP1M2* | 2 | Coronary artery disease |
| *SLC44A2* | 1 | Coronary artery disease |
| *MTMR9* | 1 | Coronary artery disease |
| *MTDH* | 2 | Coronary artery disease |
| *No data avaiable about C8ORF12 in this dataset!* | NA | Low density lipoprotein cholesterol levels |
| *FAM167A* | 1 | Low density lipoprotein cholesterol levels |
| *BLK* | 2 | Low density lipoprotein cholesterol levels |
| *LINC00208* | 2 | Low density lipoprotein cholesterol levels |
| *GATA4* | 1 | Low density lipoprotein cholesterol levels |
| *C8orf49* | NA | Low density lipoprotein cholesterol levels |
| *NEIL2* | 0 | Low density lipoprotein cholesterol levels |
| *FDFT1* | 0 | Low density lipoprotein cholesterol levels |
| *CTSB* | 3 | Low density lipoprotein cholesterol levels |
| *CHRNB3* | 1 | Low density lipoprotein cholesterol levels |
| *HGSNAT* | 0 | Low density lipoprotein cholesterol levels |
| *ENG* | 3 | Low density lipoprotein cholesterol levels |
| *PAK1* | 4 | Low density lipoprotein cholesterol levels |
| *ST6GALNAC6* | 1 | Low density lipoprotein cholesterol levels |
| *FAM102A* | 1 | Blood protein levels |
| *DEFB135* | 0 | Blood protein levels |
| *KIAA1456* | 1 | Blood protein levels |
| *RNU6-842P* | 0 | Household income |
| *No data avaiable about RP11-145O15.3 in this dataset!* | NA | General cognitive ability |
| *C8orf48* | NA | General cognitive ability |
| *SGCZ* | 2 | General cognitive ability |
| *TUSC3* | 0 | General cognitive ability |
| *MSR1* | 3 | Lymphocyte counts |
| *No data avaiable about RP11-13N12.1 in this dataset!* | NA | Lymphocyte counts |
| *RN7SL474P* | 0 | Lymphocyte counts |
| *FGF20* | 2 | Lymphocyte counts |
| *MICU3* | 0 | Lymphocyte counts |
| *ZDHHC22* | 1 | Lymphocyte counts |
| *BTAF1* | 1 | Lymphocyte counts |
| *CPEB3* | 2 | Lymphocyte counts |
| *USP31* | 1 | Low density lipoprotein cholesterol levels |
| *SCNN1G* | 2 | Low density lipoprotein cholesterol levels |
| *SCNN1B* | 2 | Low density lipoprotein cholesterol levels |
| *COG7* | 0 | Low density lipoprotein cholesterol levels |
| *PLK1* | 3 | Low density lipoprotein cholesterol levels |
| *IL27* | 2 | Low density lipoprotein cholesterol levels |
| *NUPR1* | 2 | Low density lipoprotein cholesterol levels |
| *No data avaiable about CCDC101 in this dataset!* | NA | Low density lipoprotein cholesterol levels |
| *CCDC25* | 2 | Low density lipoprotein cholesterol levels |
| *SCARA5* | 2 | Low density lipoprotein cholesterol levels |
| *SPIDR* | 0 | Low density lipoprotein cholesterol levels |
| *RAB2A* | 1 | Low density lipoprotein cholesterol levels |
| *CHD7* | 3 | Low density lipoprotein cholesterol levels |
| *No data avaiable about RP11-33I11.3 in this dataset!* | NA | Low density lipoprotein cholesterol levels |
| *CLVS1* | 1 | Low density lipoprotein cholesterol levels |
| *ASPH* | 3 | Waist circumference adjusted for body mass index |
| *DIP2C* | 2 | Waist circumference adjusted for body mass index |
| *CPA6* | 1 | Waist circumference adjusted for body mass index |
| *PREX2* | 1 | Waist circumference adjusted for body mass index |
| *C8orf34* | NA | Waist circumference adjusted for body mass index |
| *No data avaiable about RP11-600K15.1 in this dataset!* | NA | Waist circumference adjusted for body mass index |
| *SULF1* | 1 | Waist circumference adjusted for body mass index |
| *SLCO5A1* | 0 | Etoposide drug response (IC50) |
| *FZD4* | 2 | Cisplatin drug response (IC50) |
| *No data avaiable about RP11-736K20.6 in this dataset!* | NA | Type 2 diabetes |
| *TMEM135* | 0 | Type 2 diabetes |
| *No data avaiable about RP11-159H10.3 in this dataset!* | NA | Type 2 diabetes |
| *PRDM14* | 2 | Type 2 diabetes |
| *NCOA2* | 3 | Type 2 diabetes |
| *No data avaiable about RP11-298H24.1 in this dataset!* | NA | Type 2 diabetes |
| *No data avaiable about RP11-215N21.1 in this dataset!* | NA | Type 2 diabetes |
| *No data avaiable about C10ORF118 in this dataset!* | NA | Type 2 diabetes |
| *VWA2* | 1 | Type 2 diabetes |
| *AFAP1L2* | 0 | Type 2 diabetes |
| *ABLIM1* | 1 | Type 2 diabetes |
| *TAF9BP2* | 0 | Hematocrit |
| *FAM160B1* | 0 | Hematocrit |
| *No data avaiable about RP11-106M7.4 in this dataset!* | NA | Hematocrit |
| *TRUB1* | 1 | Hematocrit |
| *ATRNL1* | 1 | Hematocrit |
| *GFRA1* | 3 | Hematocrit |
| *PNLIPRP3* | 0 | Hematocrit |
| *FAM24B* | 1 | Hematocrit |
| *C10orf88* | NA | Hematocrit |
| *PSTK* | 1 | Hematocrit |
| *ACADSB* | 1 | Hematocrit |
| *HMX3* | 1 | Adolescent idiopathic scoliosis |
| *HMX2* | 1 | Adolescent idiopathic scoliosis |
| *BUB3* | 2 | Adolescent idiopathic scoliosis |
| *GPR26* | 3 | Adolescent idiopathic scoliosis |
| *CPXM2* | 0 | Adolescent idiopathic scoliosis |
| *ECHS1* | 2 | Adolescent idiopathic scoliosis |
| *PAOX* | 3 | Adolescent idiopathic scoliosis |
| *MTG1* | 0 | Adolescent idiopathic scoliosis |
| *SPRN* | 1 | Adolescent idiopathic scoliosis |
| *CYP2E1* | 2 | Total PHF-tau (SNP x SNP interaction) |
| *No data avaiable about AP006285.7 in this dataset!* | NA | Total PHF-tau (SNP x SNP interaction) |
| *FAM99A* | 0 | Total PHF-tau (SNP x SNP interaction) |
| *IFITM10* | 1 | Total PHF-tau (SNP x SNP interaction) |
| *CTSD* | 3 | Total PHF-tau (SNP x SNP interaction) |
| *LSP1* | 2 | Total PHF-tau (SNP x SNP interaction) |
| *No data avaiable about AC051649.12 in this dataset!* | NA | Total PHF-tau (SNP x SNP interaction) |
| *TNNT3* | 0 | Total PHF-tau (SNP x SNP interaction) |
| *OR51L1* | 0 | Gut microbiota relative abundance (Ruminococcus belonging to family Erysipelotrichaceae) |
| *LINC00958* | 0 | Gut microbiota relative abundance (unclassified genus belonging to family Lachnospiraceae) |
| *RASSF10* | 0 | Gut microbiota relative abundance (unclassified genus belonging to family Lachnospiraceae) |
| *No data avaiable about CTC-497E21.5 in this dataset!* | NA | Gut microbiota relative abundance (unclassified genus belonging to family Lachnospiraceae) |
| *ARNTL* | 2 | Gut microbiota relative abundance (Blautia) |
| *BTBD10* | 0 | Gut microbiota relative abundance (Blautia) |
| *PTH* | 0 | Gut microbiota relative abundance (Blautia) |
| *FAR1* | 1 | Gut microbiota relative abundance (Blautia) |
| *SPON1* | 1 | Gut microbiota relative abundance (Blautia) |
| *RRAS2* | 2 | Gut microbiota relative abundance (Blautia) |
| *COPB1* | 1 | Gut microbiota relative abundance (Blautia) |
| *PSMA1* | 1 | Gut microbiota relative abundance (Bifidobacterium) |
| *PDE3B* | 1 | Gut microbiota relative abundance (Bifidobacterium) |
| *CYP2R1* | 1 | Gut microbiota relative abundance (Bifidobacterium) |
| *CALCB* | 2 | Gut microbiota relative abundance (Bifidobacterium) |
| *CALCA* | 2 | Gut microbiota relative abundance (Bacteroides) |
| *INSC* | 1 | Gut microbiota relative abundance (Parabacteroides) |
| *No data avaiable about RP11-396O20.1 in this dataset!* | NA | Gut microbiota relative abundance (Parabacteroides) |
| *No data avaiable about RP11-396O20.2 in this dataset!* | NA | Gut microbiota relative abundance (Parabacteroides) |
| *SOX6* | 2 | Gut microbiota relative abundance (Prevotella) |
| *No data avaiable about CTD-3096P4.1 in this dataset!* | NA | Gut microbiota relative abundance (Prevotella) |
| *No data avaiable about CTD-2507G9.1 in this dataset!* | NA | Gut microbiota relative abundance (Prevotella) |
| *ANO3* | 0 | Gut microbiota relative abundance (Prevotella) |
| *MUC15* | 0 | Gut microbiota relative abundance (Streptococcus) |
| *SLC5A12* | 0 | Gut microbiota relative abundance (Streptococcus) |
| *FIBIN* | 0 | Gut microbiota relative abundance (unclassified genus belonging to family Clostridiaceae) |
| *CCDC34* | 1 | Gut microbiota relative abundance (unclassified genus belonging to family Clostridiaceae) |
| *LGR4* | 0 | Waist circumference adjusted for body mass index |
| *No data avaiable about RP11-159H22.2 in this dataset!* | NA | Waist circumference adjusted for body mass index |
| *LIN7C* | 0 | Waist circumference adjusted for body mass index |
| *BDNF-AS* | 0 | Waist circumference adjusted for body mass index |
| *BDNF-AS* | 0 | Waist circumference adjusted for body mass index |
| *KAT5* | 1 | Waist circumference adjusted for body mass index |
| *RNASEH2C* | 0 | Waist circumference adjusted for body mass index |
| *AP5B1* | 0 | Waist circumference adjusted for body mass index |
| *OVOL1* | 1 | Waist circumference adjusted for body mass index |
| *CFL1* | 2 | Reading ability (multivariate) |
| *EFEMP2* | 3 | Reading ability (multivariate) |
| *FIBP* | 2 | Reading ability (multivariate) |
| *FOSL1* | 2 | Reading ability (multivariate) |
| *DRAP1* | 2 | Reading ability (multivariate) |
| *TSGA10IP* | 0 | Rapid automised naming of letters |
| *EIF1AD* | 1 | Aspartate aminotransferase levels |
| *CST6* | 0 | Aspartate aminotransferase levels |
| *LRP5* | 2 | Aspartate aminotransferase levels |
| *PPP6R3* | 0 | Aspartate aminotransferase levels |
| *GALNT10* | 4 | Aspartate aminotransferase levels |
| *CPT1A* | 1 | Aspartate aminotransferase levels |
| *IGHMBP2* | 1 | Aspartate aminotransferase levels |
| *No data avaiable about RP11-554A11.6 in this dataset!* | NA | Aspartate aminotransferase levels |
| *TPCN2* | 3 | Aspartate aminotransferase levels |
| *No data avaiable about RP11-554A11.7 in this dataset!* | NA | Aspartate aminotransferase levels |
| *MYEOV* | 2 | Aspartate aminotransferase levels |
| *PDE2A* | 1 | Aspartate aminotransferase levels |
| *ARAP1* | 0 | Aspartate aminotransferase levels |
| *STARD10* | 3 | Aspartate aminotransferase levels |
| *ATG16L2* | 1 | Aspartate aminotransferase levels |
| *FCHSD2* | 2 | Aspartate aminotransferase levels |
| *No data avaiable about RP11-800A3.3 in this dataset!* | NA | Rapid automised naming of letters |
| *UCP2* | 0 | Male-pattern baldness |
| *KCNE3* | 1 | Male-pattern baldness |
| *POLD3* | 1 | Male-pattern baldness |
| *ZC3H12C* | 1 | Male-pattern baldness |
| *RDX* | 2 | Male-pattern baldness |
| *CEP164* | 3 | Male-pattern baldness |
| *DSCAML1* | 1 | Multisite chronic pain |
| *IL10RA* | 3 | Multisite chronic pain |
| *TMPRSS4-AS1* | 0 | Multisite chronic pain |
| *SCN2B* | 2 | Multisite chronic pain |
| *No data avaiable about AMICA1 in this dataset!* | NA | Multisite chronic pain |
| *MPZL3* | 1 | Multisite chronic pain |
| *MPZL2* | 1 | Multisite chronic pain |
| *GRAMD1B* | 0 | Multisite chronic pain |
| *SCN3B* | 1 | Multisite chronic pain |
| *ZNF202* | 0 | Multisite chronic pain |
| *OR6X1* | 1 | Multisite chronic pain |
| *OR10G7* | 2 | Multisite chronic pain |
| *DCPS* | 3 | Type 2 diabetes |
| *ST3GAL4* | 0 | Type 2 diabetes |
| *KIRREL3* | 2 | Type 2 diabetes |
| *KIRREL3-AS3* | 0 | Type 2 diabetes |
| *No data avaiable about RP11-168K9.2 in this dataset!* | NA | Type 2 diabetes |
| *No data avaiable about RP11-702B10.1 in this dataset!* | NA | Type 2 diabetes |
| *RECQL* | 0 | Type 2 diabetes |
| *No data avaiable about C12ORF39 in this dataset!* | NA | Type 2 diabetes |
| *GYS2* | 1 | Lymphocyte counts |
| *LDHB* | 1 | Lymphocyte counts |
| *KCNJ8* | 1 | Lymphocyte counts |
| *ABCC9* | 1 | Hemoglobin |
| *CMAS* | 3 | Hemoglobin |
| *ST8SIA1* | 0 | Hemoglobin |
| *C2CD5* | 0 | Hemoglobin |
| *ETNK1* | 1 | Hemoglobin |
| *No data avaiable about RP11-114G22.1 in this dataset!* | NA | Hemoglobin |
| *SOX5* | 2 | Hemoglobin |
| *MIR920* | 0 | Hemoglobin |
| *LINC00477* | 0 | Hemoglobin |
| *KNOP1P1* | 0 | Hemoglobin |
| *No data avaiable about RP11-615I16.1 in this dataset!* | NA | Blood protein levels |
| *BCAT1* | 2 | Blood protein levels |
| *IPO8* | 2 | Blood protein levels |
| *CAPRIN2* | 1 | Blood protein levels |
| *TSPAN11* | 1 | Blood protein levels |
| *FAM60A* | 0 | Blood protein levels |
| *DENND5B* | 0 | Blood protein levels |
| *No data avaiable about RP11-73B8.3 in this dataset!* | NA | Blood protein levels |
| *PUS7L* | 2 | Adolescent idiopathic scoliosis |
| *TMEM117* | 2 | Adolescent idiopathic scoliosis |
| *NELL2* | 1 | Adolescent idiopathic scoliosis |
| *ANO6* | 1 | Adolescent idiopathic scoliosis |
| *DIP2B* | 1 | Adolescent idiopathic scoliosis |
| *ATF1* | 3 | Adolescent idiopathic scoliosis |
| *METTL7A* | 1 | Adolescent idiopathic scoliosis |
| *SLC11A2* | 3 | Adolescent idiopathic scoliosis |
| *POU6F1* | 2 | Adolescent idiopathic scoliosis |
| *SMAGP* | 0 | Aspartate aminotransferase levels |
| *BIN2* | 1 | Aspartate aminotransferase levels |
| *CELA1* | 1 | Serum alkaline phosphatase levels |
| *GALNT6* | 0 | Opioid overdose severity score |
| *SLC4A8* | 2 | General cognitive ability |
| *SCN8A* | 2 | General cognitive ability |
| *ANKRD33* | 0 | General cognitive ability |
| *ACVRL1* | 1 | General cognitive ability |
| *ACVR1B* | 2 | General cognitive ability |
| *No data avaiable about C12ORF44 in this dataset!* | NA | General cognitive ability |
| *KRT80* | 1 | General cognitive ability |
| *KRT86* | 1 | Lymphocyte counts |
| *KRT85* | 0 | Lymphocyte counts |
| *KRT82* | 0 | Lymphocyte counts |
| *BAZ2A* | 2 | Lymphocyte counts |
| *ATP5B* | 1 | Lymphocyte counts |
| *PTGES3* | 1 | Lymphocyte counts |
| *NACA* | 1 | Lymphocyte counts |
| *PRIM1* | 1 | Lymphocyte counts |
| *HSD17B6* | 1 | Lymphocyte counts |
| *No data avaiable about RP11-74M13.4 in this dataset!* | NA | Lymphocyte counts |
| *SDR9C7* | 1 | Lymphocyte counts |
| *GPR182* | 0 | Lymphocyte counts |
| *HBCBP* | 0 | Lymphocyte counts |
| *ZBTB39* | 1 | Hematocrit |
| *TAC3* | 1 | Hematocrit |
| *MYO1A* | 1 | Hematocrit |
| *No data avaiable about TMEM194A in this dataset!* | NA | Hematocrit |
| *STAT6* | 2 | Hematocrit |
| *LRP1* | 1 | Motor fluctuations in levodopa treated Parkinson's disease |
| *SLC16A7* | 1 | Levodopa-induced dyskinesia in levodopa treated Parkinson's disease |
| *No data avaiable about RP11-154D9.1 in this dataset!* | NA | Levodopa-induced dyskinesia in levodopa treated Parkinson's disease |
| *FAM19A2* | 2 | Levodopa-induced dyskinesia in levodopa treated Parkinson's disease |
| *USP15* | 2 | Levodopa-induced dyskinesia in levodopa treated Parkinson's disease |
| *MON2* | 2 | Waist circumference adjusted for body mass index |
| *PHLDA1* | 3 | Ceramide levels (C24:0) |
| *NAP1L1* | 2 | Ceramide levels (C24:0) |
| *OSBPL8* | 2 | Ceramide levels (C24:0) |
| *No data avaiable about RP11-20E24.1 in this dataset!* | NA | Ceramide levels (C24:0) |
| *ZDHHC17* | 3 | Ceramide levels (C24:0) |
| *CSRP2* | 0 | Ceramide levels (C24:0) |
| *No data avaiable about C12ORF79 in this dataset!* | NA | Ceramide levels (C22:0) |
| *BTG1* | 2 | Lean nonalcoholic fatty liver disease |
| *No data avaiable about RP11-796E2.4 in this dataset!* | NA | Lean nonalcoholic fatty liver disease |
| *RPL21P106* | 0 | Bipolar disorder |
| *CLLU1OS* | 0 | Bipolar disorder |
| *C12orf74* | NA | Bipolar disorder |
| *PLEKHG7* | 0 | Bipolar disorder |
| *EEA1* | 1 | Bipolar disorder |
| *RNF19A* | 1 | Bipolar disorder |
| *MIR4471* | 0 | Type 2 diabetes |
| *No data avaiable about KB-1615E4.2 in this dataset!* | NA | Type 2 diabetes |
| *ANKRD46* | 1 | Type 2 diabetes |
| *SNX31* | 1 | Type 2 diabetes |
| *RRM2B* | 0 | Type 2 diabetes |
| *UBR5* | 2 | Type 2 diabetes |
| *RPS12P15* | 0 | Type 2 diabetes |
| *No data avaiable about KB-1980E6.3 in this dataset!* | NA | Coronary artery disease |
| *ODF1* | 1 | Coronary artery disease |
| *KLF10* | 0 | Coronary artery disease |
| *No data avaiable about RP11-127H5.1 in this dataset!* | NA | Coronary artery disease |
| *ZFPM2* | 2 | Coronary artery disease |
| *OXR1* | 3 | Coronary artery disease |
| *MAPK15* | 1 | Coronary artery disease |
| *EPPK1* | 0 | Coronary artery disease |
| *PLEC* | 0 | Coronary artery disease |
| *PARP10* | 1 | Coronary artery disease |
| *GRINA* | 1 | Coronary artery disease |
| *OPLAH* | 2 | Coronary artery disease |
| *CD274* | 0 | Coronary artery disease |
| *PDCD1LG2* | 0 | Blood protein levels |
| *No data avaiable about KIAA1432 in this dataset!* | NA | Blood protein levels |
| *ERMP1* | 1 | Blood protein levels |
| *KIAA2026* | 1 | Blood protein levels |
| *MLANA* | 0 | Blood protein levels |
| *RANBP6* | 1 | Blood protein levels |
| *No data avaiable about RP11-218I7.2 in this dataset!* | NA | Blood protein levels |
| *No data avaiable about RP11-575C20.1 in this dataset!* | NA | Lymphocyte counts |
| *IL33* | 1 | Lymphocyte counts |
| *No data avaiable about RP11-307L3.2 in this dataset!* | NA | Lymphocyte counts |
| *TPD52L3* | 0 | Lymphocyte counts |
| *GLDC* | 2 | Lymphocyte counts |
| *No data avaiable about RP11-335H2.2 in this dataset!* | NA | Lymphocyte counts |
| *CNTLN* | 1 | Alanine aminotransferase levels |
| *PABPC1P11* | 0 | Alanine aminotransferase levels |
| *ADAMTSL1* | 1 | Alanine aminotransferase levels |
| *No data avaiable about FAM154A in this dataset!* | NA | Alanine aminotransferase levels |
| *OBFC1* | 2 | Alanine aminotransferase levels |
| *COL17A1* | 0 | Alanine aminotransferase levels |
| *No data avaiable about WDR96 in this dataset!* | NA | Alanine aminotransferase levels |
| *GSTO1* | 1 | Adolescent idiopathic scoliosis |
| *TCF7L2* | 2 | Adolescent idiopathic scoliosis |
| *No data avaiable about RP11-481H12.1 in this dataset!* | NA | Adolescent idiopathic scoliosis |
| *HABP2* | 1 | Adolescent idiopathic scoliosis |
| *NRAP* | 2 | Adolescent idiopathic scoliosis |
| *CASP7* | 3 | Adolescent idiopathic scoliosis |
| *ADRB1* | 2 | Alanine aminotransferase levels |
| *UBE2V1P5* | 0 | Alanine aminotransferase levels |
| *POLR2L* | 2 | Alanine aminotransferase levels |
| *CHID1* | 1 | Alanine aminotransferase levels |
| *MUC2* | 1 | Alanine aminotransferase levels |
| *MUC5AC* | 2 | Alanine aminotransferase levels |
| *MUC5B* | 0 | Alanine aminotransferase levels |
| *BRSK2* | 3 | Alanine aminotransferase levels |
| *MOB2* | 1 | Alanine aminotransferase levels |
| *DUSP8* | 3 | Alanine aminotransferase levels |
| *ADM* | 1 | Alanine aminotransferase levels |
| *AMPD3* | 1 | Male-pattern baldness |
| *RNF141* | 3 | Male-pattern baldness |
| *LYVE1* | 1 | Male-pattern baldness |
| *MRVI1* | 1 | Male-pattern baldness |
| *EIF4G2* | 3 | Male-pattern baldness |
| *ZBED5-AS1* | 1 | Male-pattern baldness |
| *No data avaiable about AP003068.17 in this dataset!* | NA | Male-pattern baldness |
| *SLC22A20* | 0 | Male-pattern baldness |
| *POLA2* | 2 | Male-pattern baldness |
| *CDC42EP2* | 1 | Lymphocyte counts |
| *DPF2* | 1 | Lymphocyte counts |
| *SLC25A45* | 1 | Lymphocyte counts |
| *FRMD8* | 0 | Lymphocyte counts |
| *NEAT1* | 1 | Lymphocyte counts |
| *No data avaiable about AP000769.1 in this dataset!* | NA | Lymphocyte counts |
| *MALAT1* | 1 | Lymphocyte counts |
| *SCYL1* | 2 | Lymphocyte counts |
| *LTBP3* | 1 | Lymphocyte counts |
| *SSSCA1-AS1* | 0 | Lymphocyte counts |
| *SSSCA1-AS1* | 0 | Lymphocyte counts |
| *ALDH3B1* | 2 | Lymphocyte counts |
| *TCIRG1* | 3 | Carbamazepine-induced reaction with eosinophilia and systemic symptoms |
| *No data avaiable about CTD-2655K5.1 in this dataset!* | NA | Carbamazepine-induced serious cutaneous adverse reaction |
| *No data avaiable about SUV420H1 in this dataset!* | NA | Carbamazepine-induced serious cutaneous adverse reaction |
| *C11orf24* | NA | Gut microbiota alpha diversity (PD_whole_tree index) |
| *PRCP* | 0 | Gut microbiota alpha diversity (PD_whole_tree index) |
| *No data avaiable about C11ORF82 in this dataset!* | NA | Gut microbiota alpha diversity (PD_whole_tree index) |
| *RAB30* | 1 | Gut microbiota alpha diversity (PD_whole_tree index) |
| *RAB30-AS1* | 0 | Gut microbiota alpha diversity (PD_whole_tree index) |
| *PCF11* | 1 | Gut microbiota alpha diversity (PD_whole_tree index) |
| *DLG2* | 2 | Gut microbiota alpha diversity (Shannon index) |
| *TMEM126B* | 1 | Gut microbiota alpha diversity (Shannon index) |
| *HEPHL1* | 0 | Gut microbiota beta diversity (weighted UniFrac distance) |
| *PANX1* | 2 | Gut microbiota beta diversity (weighted UniFrac distance) |
| *GPR83* | 3 | General cognitive ability |
| *MRE11A* | 0 | General cognitive ability |
| *PIWIL4* | 1 | General cognitive ability |
| *FUT4* | 2 | General cognitive ability |
| *APOA4* | 2 | General cognitive ability |
| *APOC3* | 3 | Lymphocyte counts |
| *APOA1* | 3 | Lymphocyte counts |
| *SIK3* | 1 | Lymphocyte counts |
| *PAFAH1B2* | 1 | Lymphocyte counts |
| *SIDT2* | 1 | Lymphocyte counts |
| *TAGLN* | 1 | Lymphocyte counts |
| *PCSK7* | 0 | Lymphocyte counts |
| *RNF214* | 0 | Lymphocyte counts |
| *TECTA* | 1 | Aspartate aminotransferase levels |
| *No data avaiable about RP11-166D19.1 in this dataset!* | NA | Aspartate aminotransferase levels |
| *BLID* | 0 | Aspartate aminotransferase levels |
| *UBASH3B* | 1 | Aspartate aminotransferase levels |
| *CRTAM* | 0 | Aspartate aminotransferase levels |
| *C11orf63* | NA | Aspartate aminotransferase levels |
| *BSX* | 0 | Aspartate aminotransferase levels |
| *No data avaiable about RP11-335F8.2 in this dataset!* | NA | Aspartate aminotransferase levels |
| *HSPA8* | 3 | Aspartate aminotransferase levels |
| *CLMP* | 0 | Aspartate aminotransferase levels |
| *No data avaiable about CTD-2216M2.1 in this dataset!* | NA | Aspartate aminotransferase levels |
| *MIR4493* | 0 | Aspartate aminotransferase levels |
| *CLEC6A* | 0 | Aspartate aminotransferase levels |
| *No data avaiable about RP11-405A12.2 in this dataset!* | NA | Aspartate aminotransferase levels |
| *No data avaiable about RP11-664H17.1 in this dataset!* | NA | Aspartate aminotransferase levels |
| *No data avaiable about CTC-465D4.1 in this dataset!* | NA | Aspartate aminotransferase levels |
| *PDE3A* | 3 | Aspartate aminotransferase levels |
| *SLCO1C1* | 2 | Aspartate aminotransferase levels |
| *SLCO1B3* | 2 | Aspartate aminotransferase levels |
| *SLCO1B1* | 2 | Aspartate aminotransferase levels |
| *SLCO1A2* | 1 | Aspartate aminotransferase levels |
| *KMT2D* | 0 | Aspartate aminotransferase levels |
| *RHEBL1* | 2 | Aspartate aminotransferase levels |
| *ZDHHC22* | 1 | Aspartate aminotransferase levels |
| *LMBR1L* | 0 | Aspartate aminotransferase levels |
| *TUBA1B* | 3 | Aspartate aminotransferase levels |
| *TUBA1C* | 2 | Aspartate aminotransferase levels |
| *No data avaiable about RP11-161H23.5 in this dataset!* | NA | Aspartate aminotransferase levels |
| *PRPH* | 1 | Male-pattern baldness |
| *KCNH3* | 0 | Male-pattern baldness |
| *PRPF40B* | 2 | Male-pattern baldness |
| *FAM186B* | 1 | Male-pattern baldness |
| *TMBIM6* | 0 | Male-pattern baldness |
| *NCKAP5L* | 1 | Total PHF-tau (SNP x SNP interaction) |
| *BCDIN3D* | 1 | Total PHF-tau (SNP x SNP interaction) |
| *FAIM2* | 3 | Total PHF-tau (SNP x SNP interaction) |
| *No data avaiable about RP11-70F11.11 in this dataset!* | NA | Total PHF-tau (SNP x SNP interaction) |
| *AQP2* | 0 | Total PHF-tau (SNP x SNP interaction) |
| *AQP5* | 1 | Total PHF-tau (SNP x SNP interaction) |
| *GPD1* | 2 | Total PHF-tau (SNP x SNP interaction) |
| *COX14* | 0 | Total PHF-tau (SNP x SNP interaction) |
| *SLC35E3* | 0 | Total PHF-tau (SNP x SNP interaction) |
| *No data avaiable about RP11-611O2.2 in this dataset!* | NA | Total PHF-tau (SNP x SNP interaction) |
| *MDM2* | 3 | Total PHF-tau (SNP x SNP interaction) |
| *CPM* | 2 | Male-pattern baldness |
| *CPSF6* | 1 | Male-pattern baldness |
| *LYZ* | 3 | Male-pattern baldness |
| *YEATS4* | 2 | Male-pattern baldness |
| *No data avaiable about RP11-956E11.1 in this dataset!* | NA | Male-pattern baldness |
| *FRS2* | 3 | Male-pattern baldness |
| *CCT2* | 2 | Male-pattern baldness |
| *LRRC10* | 0 | Male-pattern baldness |
| *BEST3* | 0 | Eosinophil percentage of white cells |
| *MYRFL* | 0 | Eosinophil percentage of white cells |
| *No data avaiable about RP11-611E13.2 in this dataset!* | NA | Eosinophil percentage of white cells |
| *CNOT2* | 1 | Eosinophil percentage of white cells |
| *KCNMB4* | 2 | Eosinophil percentage of white cells |
| *PTPRB* | 2 | Hemoglobin concentration |
| *PTPRR* | 2 | Hemoglobin concentration |
| *RIC8B* | 2 | Hemoglobin concentration |
| *No data avaiable about C12ORF23 in this dataset!* | NA | Hemoglobin concentration |
| *CRY1* | 0 | Hemoglobin concentration |
| *KBTBD11* | 1 | Hemoglobin concentration |
| *WSCD2* | 1 | Hemoglobin concentration |
| *CMKLR1* | 1 | Hemoglobin concentration |
| *FICD* | 1 | Hemoglobin concentration |
| *TMEM119* | 2 | Hemoglobin concentration |
| *SELPLG* | 1 | Hemoglobin concentration |
| *No data avaiable about RP11-689B22.2 in this dataset!* | NA | Hemoglobin concentration |
| *CORO1C* | 1 | Hemoglobin concentration |
| *AZIN1* | 1 | Hemoglobin concentration |
| *No data avaiable about KB-1507C5.2 in this dataset!* | NA | Hemoglobin concentration |
| *FAM84B* | 1 | Hemoglobin concentration |
| *No data avaiable about RP11-89K10.1 in this dataset!* | NA | Hemoglobin concentration |
| *PCAT1* | 0 | Hemoglobin concentration |
| *GPIHBP1* | 2 | Hemoglobin concentration |
| *ZNF696* | 0 | Hemoglobin concentration |
| *TOP1MT* | 3 | Hemoglobin concentration |
| *HAUS6* | 1 | Hemoglobin concentration |
| *SCARNA8* | 0 | Hemoglobin concentration |
| *PLIN2* | 0 | Hemoglobin concentration |
| *SIT1* | 1 | Adolescent idiopathic scoliosis |
| *TPM2* | 2 | Adolescent idiopathic scoliosis |
| *TLN1* | 0 | Adolescent idiopathic scoliosis |
| *NPR2* | 2 | Adolescent idiopathic scoliosis |
| *TMEM8B* | 1 | Adolescent idiopathic scoliosis |
| *FAM221B* | 0 | Adolescent idiopathic scoliosis |
| *HRCT1* | 0 | Adolescent idiopathic scoliosis |
| *OR2S2* | 2 | Adolescent idiopathic scoliosis |
| *ZCCHC7* | 2 | Adolescent idiopathic scoliosis |
| *FRMPD1* | 0 | Adolescent idiopathic scoliosis |
| *DCAF10* | 1 | Adolescent idiopathic scoliosis |
| *TSHB* | 2 | Adolescent idiopathic scoliosis |
| *ALDH1B1* | 0 | Adolescent idiopathic scoliosis |
| *CNTNAP3* | 1 | Adolescent idiopathic scoliosis |
| *TRPM6* | 3 | White matter microstructure (fractional anisotropy) |
| *OSTF1* | 3 | White matter microstructure (fractional anisotropy) |
| *C9orf84* | NA | White matter microstructure (fractional anisotropy) |
| *A1CF* | 0 | White matter microstructure (fractional anisotropy) |
| *PRKG1* | 0 | White matter microstructure (fractional anisotropy) |
| *MIR605* | 0 | White matter microstructure (fractional anisotropy) |
| *DKK1* | 1 | White matter microstructure (fractional anisotropy) |
| *MBL2* | 1 | White matter microstructure (fractional anisotropy) |
| *STRBP* | 3 | White matter microstructure (fractional anisotropy) |
| *CRB2* | 1 | White matter microstructure (fractional anisotropy) |
| *DENND1A* | 1 | White matter microstructure (fractional anisotropy) |
| *TTC16* | 1 | White matter microstructure (fractional anisotropy) |
| *SH2D3C* | 1 | White matter microstructure (fractional anisotropy) |
| *CELSR2* | 2 | PR interval |
| *RALGDS* | 2 | PR interval |
| *REXO4* | 1 | PR interval |
| *ADAMTS13* | 2 | PR interval |
| *FAM107B* | 1 | PR interval |
| *ASAH2* | 1 | PR interval |
| *AKR1B10P1* | 0 | PR interval |
| *DNAJC12* | 2 | PR interval |
| *RNU6-478P* | 0 | PR interval |
| *GHITM* | 2 | Schizophrenia (MTAG) |
| *C10orf99* | NA | Schizophrenia (MTAG) |
| *LRIT2* | 1 | Schizophrenia (MTAG) |
| *RGR* | 1 | Schizophrenia (MTAG) |
| *MINPP1* | 2 | Schizophrenia (MTAG) |
| *PAPSS2* | 1 | Schizophrenia (MTAG) |
| *ATAD1* | 0 | Attention deficit hyperactivity disorder |
| *CFL1P1* | 0 | Attention deficit hyperactivity disorder |
| *No data avaiable about PPAPDC1A in this dataset!* | NA | Hemoglobin concentration |
| *ADAM12* | 2 | Hemoglobin concentration |
| *OLFML1* | 2 | Hemoglobin concentration |
| *OVCH2* | 0 | Hemoglobin concentration |
| *PPFIBP2* | 3 | Diffuse plaques (SNP x SNP interaction) |
| *CYB5R2* | 1 | Diffuse plaques (SNP x SNP interaction) |
| *ZNF143* | 2 | Diffuse plaques (SNP x SNP interaction) |
| *WEE1* | 2 | Diffuse plaques (SNP x SNP interaction) |
| *OR10A6* | 0 | Diffuse plaques (SNP x SNP interaction) |
| *NLRP10* | 0 | Arterial stiffness (carotid-femoral pulse wave velocity) |
| *EIF3FP3* | 0 | General cognitive ability |
| *TUBA1A* | 4 | General cognitive ability |
| *RIC3* | 3 | General cognitive ability |
| *LMO1* | 0 | General cognitive ability |
| *IPO7* | 1 | General cognitive ability |
| *SWAP70* | 3 | General cognitive ability |
| *SBF2-AS1* | 0 | Schizophrenia (MTAG) |
| *SAA1* | 3 | Schizophrenia (MTAG) |
| *HPS5* | 1 | Schizophrenia (MTAG) |
| *GTF2H1* | 3 | Schizophrenia (MTAG) |
| *ANO5* | 1 | Schizophrenia (MTAG) |
| *No data avaiable about CTD-2140G10.1 in this dataset!* | NA | Schizophrenia (MTAG) |
| *No data avaiable about RP11-34N19.1 in this dataset!* | NA | Schizophrenia (MTAG) |
| *LDHC* | 0 | Coronary artery disease |
| *SPTY2D1* | 0 | Coronary artery disease |
| *TMEM86A* | 0 | Coronary artery disease |
| *IGSF22* | 1 | Coronary artery disease |
| *PTPN5* | 3 | Coronary artery disease |
| *CSRP3* | 1 | Coronary artery disease |
| *No data avaiable about RP11-428C19.4 in this dataset!* | NA | Coronary artery disease |
| *NAV2* | 2 | Coronary artery disease |
| *DBX1* | 1 | Coronary artery disease |
| *HTATIP2* | 1 | Coronary artery disease |
| *PRMT3* | 2 | Coronary artery disease |
| *SLC6A5* | 1 | Coronary artery disease |
| *NELL1* | 1 | Coronary artery disease |
| *TRIM49B* | 0 | Coronary artery disease |
| *TRIM64C* | 0 | Coronary artery disease |
| *FOLH1* | 1 | Coronary artery disease |
| *No data avaiable about EMR2 in this dataset!* | NA | Coronary artery disease |
| *OR7A10* | 0 | Coronary artery disease |
| *TYRL* | 0 | Coronary artery disease |
| *No data avaiable about RP11-707M1.1 in this dataset!* | NA | Coronary artery disease |
| *OR5R1* | 0 | Coronary artery disease |
| *OR5AP2* | 1 | Coronary artery disease |
| *OR9Q1* | 0 | Coronary artery disease |
| *OR5B12* | 1 | Diffuse plaques (SNP x SNP interaction) |
| *OR5B21* | 1 | Diffuse plaques (SNP x SNP interaction) |
| *LPXN* | 2 | Diffuse plaques (SNP x SNP interaction) |
| *ZFP91* | 0 | Diffuse plaques (SNP x SNP interaction) |
| *CNTF* | 2 | Diffuse plaques (SNP x SNP interaction) |
| *GLYAT* | 1 | Diffuse plaques (SNP x SNP interaction) |
| *No data avaiable about AP000445.3 in this dataset!* | NA | Type 2 diabetes |
| *OR5B3* | 0 | Type 2 diabetes |
| *EHBP1L1* | 1 | Type 2 diabetes |
| *MAP3K11* | 3 | Type 2 diabetes |
| *No data avaiable about PCNXL3 in this dataset!* | NA | Type 2 diabetes |
| *SIPA1L2* | 1 | Type 2 diabetes |
| *RN7SL309P* | 0 | Type 2 diabetes |
| *RHOD* | 2 | Type 2 diabetes |
| *KDM2A* | 2 | Type 2 diabetes |
| *SSH3* | 0 | Type 2 diabetes |
| *CLCF1* | 2 | Type 2 diabetes |
| *CARNS1* | 2 | Type 2 diabetes |
| *RPS6KB2* | 3 | Type 2 diabetes |
| *PTPRCAP* | 1 | Type 2 diabetes |
| *CORO1B* | 3 | Type 2 diabetes |
| *CABP4* | 1 | Type 2 diabetes |
| *NAALAD2* | 1 | Type 2 diabetes |
| *CHORDC1* | 1 | Type 2 diabetes |
| *DISC1FP1* | 1 | General cognitive ability |
| *MIR4490* | 0 | General cognitive ability |
| *MIR1261* | 0 | General cognitive ability |
| *OPCML* | 0 | General cognitive ability |
| *No data avaiable about RP11-448P19.1 in this dataset!* | NA | General cognitive ability |
| *SPATA19* | 1 | General cognitive ability |
| *No data avaiable about RP11-338H14.1 in this dataset!* | NA | General cognitive ability |
| *No data avaiable about RP11-644L4.1 in this dataset!* | NA | General cognitive ability |
| *FAM76B* | 0 | Lymphocyte counts |
| *CEP57* | 2 | Lymphocyte counts |
| *FOXM1* | 1 | Lymphocyte counts |
| *TEAD4* | 2 | Lymphocyte counts |
| *TSPAN9* | 0 | Lymphocyte counts |
| *PRMT8* | 1 | Lymphocyte counts |
| *No data avaiable about EFCAB4B in this dataset!* | NA | Lymphocyte counts |
| *PARP11* | 1 | Lymphocyte counts |
| *No data avaiable about RP11-264F23.3 in this dataset!* | NA | Lymphocyte counts |
| *CCND2* | 1 | Lymphocyte counts |
| *No data avaiable about C12ORF5 in this dataset!* | NA | Lymphocyte counts |
| *FGF6* | 1 | Lymphocyte counts |
| *C12orf4* | NA | Lymphocyte counts |
| *DYRK4* | 1 | Hematocrit |
| *GALNT8* | 1 | Hematocrit |
| *RN7SL222P* | 0 | Hematocrit |
| *No data avaiable about CTD-2383M3.1 in this dataset!* | NA | Hematocrit |
| *ARHGAP42* | 1 | Hematocrit |
| *PGR* | 2 | Hematocrit |
| *TRPC6* | 3 | Hematocrit |
| *C11orf70* | NA | Type 2 diabetes |
| *YAP1* | 3 | Type 2 diabetes |
| *TMEM123* | 1 | Type 2 diabetes |
| *MMP7* | 1 | Type 2 diabetes |
| *MMP20* | 0 | Type 2 diabetes |
| *No data avaiable about RP11-817J15.2 in this dataset!* | NA | Type 2 diabetes |
| *MMP8* | 0 | Type 2 diabetes |
| *MMP10* | 3 | Type 2 diabetes |
| *MMP10* | 3 | Type 2 diabetes |
| *MMP3* | 3 | Type 2 diabetes |
| *MMP12* | 0 | Type 2 diabetes |
| *C11orf53* | NA | Type 2 diabetes |
| *COLCA2* | 0 | Type 2 diabetes |
| *MIR4491* | 0 | Type 2 diabetes |
| *POU2AF1* | 0 | Type 2 diabetes |
| *LAYN* | 1 | Type 2 diabetes |
| *SIK2* | 3 | Type 2 diabetes |
| *IL18* | 2 | Type 2 diabetes |
| *BCO2* | 0 | Type 2 diabetes |
| *PTS* | 1 | Lymphocyte counts |
| *No data avaiable about RP11-65M17.3 in this dataset!* | NA | Lymphocyte counts |
| *NCAM1* | 2 | Lymphocyte counts |
| *TTC12* | 1 | Lymphocyte counts |
| *KLRB1* | 0 | Lymphocyte counts |
| *CLEC2D* | 1 | Lymphocyte counts |
| *CLECL1* | 1 | Lymphocyte counts |
| *CD69* | 0 | Hematocrit |
| *KLRF1* | 1 | Hematocrit |
| *No data avaiable about KLRAP1 in this dataset!* | NA | Hematocrit |
| *YBX3* | 2 | Hematocrit |
| *ETV6* | 0 | Adolescent idiopathic scoliosis |
| *BCL2L14* | 2 | Adolescent idiopathic scoliosis |
| *NLRP6* | 1 | Adolescent idiopathic scoliosis |
| *MANSC1* | 0 | Adolescent idiopathic scoliosis |
| *No data avaiable about LOH12CR1 in this dataset!* | NA | Adolescent idiopathic scoliosis |
| *DUSP16* | 2 | Adolescent idiopathic scoliosis |
| *CREBL2* | 1 | Adolescent idiopathic scoliosis |
| *GPR19* | 1 | Adolescent idiopathic scoliosis |
| *CDKN1B* | 2 | Adolescent idiopathic scoliosis |
| *APOLD1* | 0 | Adolescent idiopathic scoliosis |
| *GPRC5A* | 1 | Adolescent idiopathic scoliosis |
| *No data avaiable about RP11-392P7.6 in this dataset!* | NA | Adolescent idiopathic scoliosis |
| *EFEMP1* | 2 | Adolescent idiopathic scoliosis |
| *No data avaiable about C12ORF36 in this dataset!* | NA | Self-rated health |
| *GRIN2B* | 2 | Self-rated health |
| *RNU6-491P* | 0 | Self-rated health |
| *RPL30P11* | 0 | Fractures (paediatric) |
| *ATF7IP* | 2 | Hematocrit |
| *PLBD1* | 1 | Hematocrit |
| *MIR4697* | 0 | Hematocrit |
| *IGSF9B* | 2 | Hematocrit |
| *JAM3* | 1 | Hematocrit |
| *NCAPD3* | 1 | Simvastatin-induced myopathy |
| *THYN1* | 1 | Simvastatin-induced myopathy |
| *ACAD8* | 2 | Simvastatin-induced myopathy |
| *B3GAT1* | 1 | Simvastatin-induced myopathy |
| *No data avaiable about AP003062.1 in this dataset!* | NA | Simvastatin-induced myopathy |
| *IQSEC3* | 1 | Low density lipoprotein cholesterol levels |
| *SLC6A12* | 1 | Low density lipoprotein cholesterol levels |
| *SLC6A13* | 1 | Low density lipoprotein cholesterol levels |
| *KDM5A* | 1 | Low density lipoprotein cholesterol levels |
| *CCDC77* | 0 | Low density lipoprotein cholesterol levels |
| *B4GALNT3* | 1 | Low density lipoprotein cholesterol levels |
| *NINJ2* | 3 | Total PHF-tau (SNP x SNP interaction) |
| *WNK1* | 1 | Total PHF-tau (SNP x SNP interaction) |
| *RAD52* | 0 | Total PHF-tau (SNP x SNP interaction) |
| *ERC1* | 2 | Total PHF-tau (SNP x SNP interaction) |
| *LINC00942* | 0 | Total PHF-tau (SNP x SNP interaction) |
| *WNT5B* | 0 | Total PHF-tau (SNP x SNP interaction) |
| *ADIPOR2* | 2 | Total PHF-tau (SNP x SNP interaction) |
| *CACNA2D4* | 1 | Total PHF-tau (SNP x SNP interaction) |
| *DCP1B* | 0 | Total PHF-tau (SNP x SNP interaction) |
| *C12orf60* | NA | Total PHF-tau (SNP x SNP interaction) |
| *ART4* | 1 | Low density lipoprotein cholesterol levels |
| *MANSC4* | 0 | Low density lipoprotein cholesterol levels |
| *KRT80* | 1 | Low density lipoprotein cholesterol levels |
| *KRT18* | 2 | Low density lipoprotein cholesterol levels |
| *B3GALT5* | 3 | Low density lipoprotein cholesterol levels |
| *DCDC2* | 0 | Low density lipoprotein cholesterol levels |
| *TESPA1* | 0 | Low density lipoprotein cholesterol levels |
| *ANKRD52* | 0 | Low density lipoprotein cholesterol levels |
| *PCSK9* | 4 | Low density lipoprotein cholesterol levels |
| *CNPY2* | 1 | Low density lipoprotein cholesterol levels |
| *TSPAN2* | 1 | Low density lipoprotein cholesterol levels |
| *IL23A* | 1 | Low density lipoprotein cholesterol levels |
| *STAT2* | 2 | Low density lipoprotein cholesterol levels |
| *APOF* | 2 | Low density lipoprotein cholesterol levels |
| *TIMELESS* | 0 | Simvastatin-induced myopathy |
| *SPRYD4* | 2 | Atorvastatin-induced myopathy |
| *GLS2* | 2 | Atorvastatin-induced myopathy |
| *RBMS2* | 2 | Atorvastatin-induced myopathy |
| *KRR1* | 3 | Atorvastatin-induced myopathy |
| *No data avaiable about RP11-114H23.1 in this dataset!* | NA | Atorvastatin-induced myopathy |
| *No data avaiable about RP11-486A14.2 in this dataset!* | NA | Atorvastatin-induced myopathy |
| *NUDT4* | 3 | Statin-induced myopathy (severe) |
| *MRPL42* | 2 | Statin-induced myopathy (severe) |
| *RN7SL737P* | 0 | Length of menstrual cycle |
| *SOCS2-AS1* | 0 | Length of menstrual cycle |
| *SOCS2-AS1* | 0 | Myocardial infarction |
| *CRADD* | 0 | Myocardial infarction |
| *No data avaiable about RP11-1060G2.2 in this dataset!* | NA | Myocardial infarction |
| *No data avaiable about RP11-74K11.1 in this dataset!* | NA | Myocardial infarction |
| *TRAFD1* | 1 | Myocardial infarction |
| *HECTD4* | 1 | Myocardial infarction |
| *RPL6* | 2 | Myocardial infarction |
| *PTPN11* | 3 | Myocardial infarction |
| *OAS1* | 3 | Myocardial infarction |
| *OAS3* | 2 | Myocardial infarction |
| *SDS* | 2 | Myocardial infarction |
| *LHX5* | 2 | Myocardial infarction |
| *RBM19* | 1 | Myocardial infarction |
| *No data avaiable about SETD8 in this dataset!* | NA | Myocardial infarction |
| *RILPL2* | 0 | Myocardial infarction |
| *DDX55* | 0 | Myocardial infarction |
| *GTF2H3* | 2 | Myocardial infarction |
| *DHX37* | 2 | Celiac disease |
| *AACSP1* | 0 | Celiac disease |
| *TMEM132B* | 0 | Male-pattern baldness |
| *No data avaiable about RP11-575F12.1 in this dataset!* | NA | Male-pattern baldness |
| *No data avaiable about RP11-955H22.1 in this dataset!* | NA | Male-pattern baldness |
| *No data avaiable about RP11-351O2.1 in this dataset!* | NA | Male-pattern baldness |
| *TMEM132C* | 2 | Male-pattern baldness |
| *SLC15A4* | 2 | Male-pattern baldness |
| *GLT1D1* | 1 | Male-pattern baldness |
| *TMEM132D* | 2 | Male-pattern baldness |
| *PIWIL1* | 1 | Male-pattern baldness |
| *RIMBP2* | 1 | Male-pattern baldness |
| *STX2* | 1 | Waist circumference adjusted for body mass index |
| *No data avaiable about GPR133 in this dataset!* | NA | Waist circumference adjusted for body mass index |
| *No data avaiable about RP11-292I17.1 in this dataset!* | NA | Waist circumference adjusted for body mass index |
| *SFSWAP* | 0 | Waist circumference adjusted for body mass index |
| *MMP17* | 1 | Waist circumference adjusted for body mass index |
| *ULK1* | 1 | Waist circumference adjusted for body mass index |
| *EP400* | 2 | Waist circumference adjusted for body mass index |
| *EP400NL* | 1 | Waist circumference adjusted for body mass index |
| *DDX51* | 1 | Waist circumference adjusted for body mass index |
| *NOC4L* | 2 | Waist circumference adjusted for body mass index |
| *No data avaiable about RP13-977J11.2 in this dataset!* | NA | Waist circumference adjusted for body mass index |
| *GALNT9* | 3 | Waist circumference adjusted for body mass index |
| *RNASE6* | 1 | Waist circumference adjusted for body mass index |
| *RNASE1* | 3 | High myopia |
| *RNASE3* | 0 | High myopia |
| *RNASE2* | 0 | High myopia |
| *METTL17* | 0 | High myopia |
| *NDRG2* | 2 | High myopia |
| *BEGAIN* | 1 | High myopia |
| *SPRED1* | 2 | High myopia |
| *RASGRP1* | 1 | High myopia |
| *No data avaiable about RP11-275I4.2 in this dataset!* | NA | Hemoglobin |
| *C15orf53* | NA | Hemoglobin |
| *No data avaiable about RP11-624L4.1 in this dataset!* | NA | Hemoglobin |
| *No data avaiable about RP11-265N7.2 in this dataset!* | NA | Hemoglobin |
| *C15orf54* | NA | Hemoglobin |
| *THBS1* | 2 | Hemoglobin |
| *EIF2AK4* | 0 | Hemoglobin |
| *SRP14* | 3 | Hemoglobin |
| *SRP14-AS1* | 0 | Iron status biomarkers (ferritin levels) |
| *BMF* | 3 | Iron status biomarkers (ferritin levels) |
| *ANKRD20A9P* | 0 | Iron status biomarkers (ferritin levels) |
| *TUBA3C* | 1 | Iron status biomarkers (ferritin levels) |
| *TPTE2* | 1 | Iron status biomarkers (ferritin levels) |
| *TRIM13* | 1 | Iron status biomarkers (ferritin levels) |
| *KCNRG* | 1 | Iron status biomarkers (total iron binding capacity) |
| *DLEU2* | 1 | Iron status biomarkers (total iron binding capacity) |
| *DLEU1* | 0 | Iron status biomarkers (total iron binding capacity) |
| *LINC00871* | 0 | Iron status biomarkers (ferritin levels) |
| *RPL10L* | 1 | Iron status biomarkers (ferritin levels) |
| *MDGA2* | 1 | Iron status biomarkers (ferritin levels) |
| *LINC00648* | 0 | Iron status biomarkers (ferritin levels) |
| *No data avaiable about RP11-326E7.1 in this dataset!* | NA | Iron status biomarkers (ferritin levels) |
| *RPS29* | 1 | Male-pattern baldness |
| *MGAT2* | 1 | Male-pattern baldness |
| *DNAAF2* | 0 | Male-pattern baldness |
| *POLE2* | 1 | Male-pattern baldness |
| *KLHDC1* | 3 | Male-pattern baldness |
| *DIO3OS* | 0 | Male-pattern baldness |
| *DIO3OS* | 0 | Male-pattern baldness |
| *PPP2R5C* | 1 | Male-pattern baldness |
| *WDR20* | 1 | Male-pattern baldness |
| *MOK* | 1 | Male-pattern baldness |
| *ZNF839* | 0 | Male-pattern baldness |
| *TECPR2* | 1 | Male-pattern baldness |
| *ANKRD9* | 1 | Gut microbiota relative abundance (unclassified genus belonging to family Clostridiaceae) |
| *No data avaiable about CTD-2555C10.3 in this dataset!* | NA | Gut microbiota relative abundance (unclassified genus belonging to family Clostridiaceae) |
| *RN7SL546P* | 0 | Gut microbiota relative abundance (unclassified genus belonging to family Clostridiaceae) |
| *RCOR1* | 1 | Gut microbiota relative abundance (unclassified genus belonging to the order Clostridiales) |
| *TRAF3* | 3 | Gut microbiota relative abundance (unclassified genus belonging to the order Clostridiales) |
| *PSPC1* | 2 | Gut microbiota relative abundance (unclassified genus belonging to the order Clostridiales) |
| *ZMYM5* | 0 | Gut microbiota relative abundance (unclassified genus belonging to the order Clostridiales) |
| *ZMYM2* | 1 | Gut microbiota relative abundance (unclassified genus belonging to the order Clostridiales) |
| *GJA3* | 0 | Gut microbiota relative abundance (unclassified genus belonging to the order Clostridiales) |
| *CRYL1* | 3 | Gut microbiota relative abundance (unclassified genus belonging to the order Clostridiales) |
| *IFT88* | 1 | Gut microbiota relative abundance (Ruminococcus belonging to family Erysipelotrichaceae) |
| *No data avaiable about AL512655.1 in this dataset!* | NA | Gut microbiota relative abundance (Ruminococcus belonging to family Erysipelotrichaceae) |
| *MIR5007* | 1 | Gut microbiota relative abundance (unclassified genus belonging to family Ruminococcaceae) |
| *HNF4GP1* | 0 | Gut microbiota relative abundance (unclassified genus belonging to family Ruminococcaceae) |
| *PRR20A* | 0 | Gut microbiota relative abundance (unclassified genus belonging to family Ruminococcaceae) |
| *PRR20E* | 0 | Gut microbiota relative abundance (unclassified genus belonging to family Ruminococcaceae) |
| *PCDH17* | 3 | Gut microbiota relative abundance (unclassified genus belonging to family Ruminococcaceae) |
| *RNA5SP30* | 0 | Gut microbiota relative abundance (unclassified genus belonging to family Ruminococcaceae) |
| *LINC00374* | 0 | Gut microbiota relative abundance (unclassified genus belonging to family Ruminococcaceae) |
| *MMP14* | 3 | Gut microbiota relative abundance (Faecalibacterium) |
| *NLRP10* | 0 | Gut microbiota relative abundance (Oscillospira) |
| *RBM23* | 0 | Gut microbiota relative abundance (Coprococcus) |
| *PRMT5* | 2 | Gut microbiota relative abundance (Coprococcus) |
| *No data avaiable about RP11-298I3.1 in this dataset!* | NA | Gut microbiota relative abundance (Coprococcus) |
| *HAUS4* | 1 | Gut microbiota relative abundance (Coprococcus) |
| *AJUBA* | 1 | Gut microbiota relative abundance (Dorea) |
| *SOS2* | 1 | Adult diffuse glioma (IDH mutation) |
| *L2HGDH* | 1 | Adult diffuse glioma (IDH mutation) |
| *CDKL1* | 1 | Adult diffuse glioma (IDH mutation) |
| *MAP4K5* | 1 | Adult diffuse glioma (IDH mutation) |
| *RPL3P3* | 0 | Adult diffuse glioma (IDH wildtype) |
| *EXOC5* | 1 | Adult diffuse glioma (IDH wildtype) |
| *NAA30* | 1 | Adult diffuse glioma (IDH mutation |
| *SLC35F4* | 3 | 1p/19q codeletion) |
| *C14orf37* | NA | Adult diffuse glioma (IDH mutation |
| *No data avaiable about RP11-349A22.5 in this dataset!* | NA | 1p/19q non-codeleted) |
| *ARID4A* | 1 | 1p/19q non-codeleted) |
| *KIAA0586* | 1 | Adult diffuse glioma (IDH mutation |
| *DACT1* | 3 | 1p/19q non-codeleted) |
| *No data avaiable about CTD-2315A10.2 in this dataset!* | NA | 1p19q codeletion) |
| *No data avaiable about CTD-2315A10.1 in this dataset!* | NA | Alanine aminotransferase levels |
| *DAAM1* | 2 | Alanine aminotransferase levels |
| *GPR135* | 1 | Alanine aminotransferase levels |
| *CCDC175* | 0 | Alanine aminotransferase levels |
| *CDC42BPB* | 3 | Alanine aminotransferase levels |
| *No data avaiable about RP11-736N17.8 in this dataset!* | NA | Alanine aminotransferase levels |
| *EXOC3L4* | 0 | Alanine aminotransferase levels |
| *TNFAIP2* | 2 | Alanine aminotransferase levels |
| *EIF5* | 1 | Alanine aminotransferase levels |
| *No data avaiable about RP11-600F24.1 in this dataset!* | NA | Alanine aminotransferase levels |
| *MARK3* | 2 | Alanine aminotransferase levels |
| *IGHE* | 0 | Alanine aminotransferase levels |
| *IGHG2* | 0 | Alanine aminotransferase levels |
| *IGHA1* | 0 | Alanine aminotransferase levels |
| *IGHEP1* | 0 | Mitochondrial DNA copy number |
| *IGHG1* | 1 | Mitochondrial DNA copy number |
| *IGHG3* | 0 | Mitochondrial DNA copy number |
| *No data avaiable about RP11-417P24.1 in this dataset!* | NA | Mitochondrial DNA copy number |
| *No data avaiable about AC124997.1 in this dataset!* | NA | Mitochondrial DNA copy number (white blood cells) |
| *ATP10A* | 2 | Factor VII activity |
| *No data avaiable about RP11-1084I9.1 in this dataset!* | NA | Factor VII activity |
| *No data avaiable about AC009878.2 in this dataset!* | NA | Factor VII activity |
| *GABRB3* | 3 | Factor VII activity |
| *GABRA5* | 1 | Factor VII activity |
| *GABRG3* | 1 | Gut microbiota relative abundance (Sutterella) |
| *SLC24A5* | 0 | Gut microbiota relative abundance (Prevotella) |
| *SLC12A1* | 1 | Gut microbiota relative abundance (Faecalibacterium) |
| *DUT* | 1 | Gut microbiota relative abundance (Faecalibacterium) |
| *FBN1* | 2 | Gut microbiota relative abundance (Oscillospira) |
| *No data avaiable about RP11-227D13.1 in this dataset!* | NA | Gut microbiota relative abundance (Oscillospira) |
| *CEP152* | 2 | Gut microbiota relative abundance (Oscillospira) |
| *QRICH2* | 1 | General cognitive ability |
| *PRPSAP1* | 1 | General cognitive ability |
| *SPHK1* | 2 | General cognitive ability |
| *UBE2O* | 1 | General cognitive ability |
| *AANAT* | 0 | General cognitive ability |
| *RHBDF2* | 0 | Lymphocyte counts |
| *PRCD* | 1 | Lymphocyte counts |
| *SRSF10P1* | 0 | Lymphocyte counts |
| *MEX3C* | 0 | Lymphocyte counts |
| *No data avaiable about RP11-267C16.1 in this dataset!* | NA | Total PHF-tau (SNP x SNP interaction) |
| *No data avaiable about RP11-25O3.1 in this dataset!* | NA | Aspartate aminotransferase levels |
| *SDCCAG3P2* | 0 | Aspartate aminotransferase levels |
| *MBD2* | 1 | Male-pattern baldness |
| *POLI* | 2 | Male-pattern baldness |
| *STARD6* | 3 | Male-pattern baldness |
| *RAB27B* | 1 | Male-pattern baldness |
| *CCDC68* | 0 | Male-pattern baldness |
| *No data avaiable about CTD-2171N6.1 in this dataset!* | NA | Male-pattern baldness |
| *TCF4* | 2 | Waist circumference adjusted for body mass index |
| *No data avaiable about RP11-397A16.1 in this dataset!* | NA | Waist circumference adjusted for body mass index |
| *No data avaiable about RP11-397A16.3 in this dataset!* | NA | Waist circumference adjusted for body mass index |
| *ZNF426* | 2 | Waist circumference adjusted for body mass index |
| *ZNF561* | 1 | Type 2 diabetes |
| *ZNF490* | 1 | Type 2 diabetes |
| *MAN2B1* | 3 | Type 2 diabetes |
| *DHPS* | 2 | General cognitive ability |
| *No data avaiable about CTD-2192J16.26 in this dataset!* | NA | General cognitive ability |
| *TNPO2* | 2 | General cognitive ability |
| *LYL1* | 2 | Lymphocyte counts |
| *TRMT1L* | 1 | Lymphocyte counts |
| *NACC1* | 1 | Lymphocyte counts |
| *No data avaiable about CTC-250I14.3 in this dataset!* | NA | Lymphocyte counts |
| *STX10* | 1 | Lymphocyte counts |
| *IER2* | 1 | Lymphocyte counts |
| *CACNA1A* | 3 | Lymphocyte counts |
| *MIR27A* | 0 | Lymphocyte counts |
| *NANOS3* | 0 | Lymphocyte counts |
| *RFPL4A* | 0 | Waist circumference adjusted for body mass index |
| *NLRP11* | 0 | Waist circumference adjusted for body mass index |
| *NLRP4* | 1 | Waist circumference adjusted for body mass index |
| *NLRP13* | 1 | Waist circumference adjusted for body mass index |
| *NLRP8* | 0 | Waist circumference adjusted for body mass index |
| *ZNF787* | 1 | Waist circumference adjusted for body mass index |
| *GALP* | 2 | General cognitive ability |
| *RNF6* | 1 | General cognitive ability |
| *CDK8* | 2 | General cognitive ability |
| *WASF3* | 1 | General cognitive ability |
| *No data avaiable about GPR126 in this dataset!* | NA | General cognitive ability |
| *USP12* | 2 | Lymphocyte counts |
| *RPL21P110* | 0 | Lymphocyte counts |
| *GNPNAT1* | 1 | Lymphocyte counts |
| *FERMT2* | 2 | Lymphocyte counts |
| *DDHD1* | 1 | Lymphocyte counts |
| *No data avaiable about RP11-547D23.1 in this dataset!* | NA | Lymphocyte counts |
| *No data avaiable about AL163953.3 in this dataset!* | NA | Lymphocyte counts |
| *No data avaiable about AL162759.1 in this dataset!* | NA | Lymphocyte counts |
| *MIR5580* | 1 | Lymphocyte counts |
| *BMP4* | 2 | Myocardial infarction |
| *CDKN3* | 0 | Myocardial infarction |
| *SAMD4A* | 1 | Myocardial infarction |
| *GCH1* | 2 | Myocardial infarction |
| *WDHD1* | 1 | Myocardial infarction |
| *SOCS4* | 1 | Lymphocyte counts |
| *MAPK1IP1L* | 1 | Lymphocyte counts |
| *LGALS3* | 3 | Lymphocyte counts |
| *No data avaiable about RP11-698F20.3 in this dataset!* | NA | Lymphocyte counts |
| *No data avaiable about RP11-665C16.6 in this dataset!* | NA | Lymphocyte counts |
| *FBXO34* | 1 | Lymphocyte counts |
| *ATG14* | 1 | Lymphocyte counts |
| *TBPL2* | 1 | Lymphocyte counts |
| *KTN1-AS1* | 0 | Lymphocyte counts |
| *RPL7AP4* | 0 | Myocardial infarction |
| *KTN1-AS1* | 0 | Myocardial infarction |
| *RPL13AP3* | 0 | Myocardial infarction |
| *LINC00520* | 0 | Type 2 diabetes |
| *No data avaiable about AL163952.2 in this dataset!* | NA | Type 2 diabetes |
| *TMEM260* | 0 | Type 2 diabetes |
| *OTX2* | 0 | Type 2 diabetes |
| *OTX2-AS1* | 0 | Type 2 diabetes |
| *GTF3A* | 3 | Type 2 diabetes |
| *MTIF3* | 2 | Type 2 diabetes |
| *LNX2* | 3 | Type 2 diabetes |
| *GSX1* | 1 | Hemoglobin |
| *PDX1-AS1* | 0 | Hemoglobin |
| *PDX1-AS1* | 0 | Hemoglobin |
| *CDX2* | 1 | Hemoglobin |
| *FLT3LG* | 1 | Hemoglobin |
| *TSPAN32* | 2 | Hemoglobin |
| *LINC00347* | 0 | Hemoglobin |
| *No data avaiable about AL162571.1 in this dataset!* | NA | Hemoglobin |
| *CTAGE11P* | 1 | Hemoglobin |
| *TBC1D4* | 1 | Hemoglobin |
| *COMMD6* | 1 | Hemoglobin |
| *UCHL3* | 2 | Word spelling |
| *LMO7* | 1 | Non-word reading |
| *No data avaiable about C13ORF45 in this dataset!* | NA | Word spelling |
| *CLN5* | 2 | Non-word reading |
| *FBXL3* | 1 | Word reading |
| *MYCBP2* | 0 | Word reading |
| *SCEL* | 1 | Word reading |
| *SLAIN1* | 1 | Word reading |
| *EDNRB-AS1* | 0 | Word reading |
| *EDNRB-AS1* | 0 | Phoneme awareness |
| *RNF219-AS1* | 0 | Phoneme awareness |
| *LINC00446* | 0 | Phoneme awareness |
| *POU4F1* | 1 | Phoneme awareness |
| *RNF219-AS1* | 0 | Phoneme awareness |
| *ZNF841* | 1 | Phoneme awareness |
| *ZNF616* | 0 | Phoneme awareness |
| *ZNF578* | 1 | Reading ability (multivariate) |
| *ZNF808* | 1 | Word spelling |
| *ZNF701* | 0 | Non-word reading |
| *NBEA* | 0 | Non-word reading |
| *ALG5* | 1 | Word spelling |
| *SUPT20H* | 0 | Alanine aminotransferase levels |
| *CSNK1A1L* | 0 | Alanine aminotransferase levels |
| *LINC01048* | 0 | Alanine aminotransferase levels |
| *POSTN* | 2 | Alanine aminotransferase levels |
| *TRPC4* | 2 | Alanine aminotransferase levels |
| *UFM1* | 2 | Alanine aminotransferase levels |
| *FREM2* | 1 | Alanine aminotransferase levels |
| *STOML3* | 0 | Alanine aminotransferase levels |
| *LHFPL2* | 2 | Aspartate aminotransferase levels |
| *COG6* | 3 | Aspartate aminotransferase levels |
| *LINC00332* | 1 | Aspartate aminotransferase levels |
| *RN7SKP229* | 0 | Aspartate aminotransferase levels |
| *LINC00598* | 0 | Aspartate aminotransferase levels |
| *No data avaiable about AL133318.1 in this dataset!* | NA | Aspartate aminotransferase levels |
| *C15orf41* | NA | Aspartate aminotransferase levels |
| *MEIS2* | 1 | Aspartate aminotransferase levels |
| *TMCO5A* | 0 | Aspartate aminotransferase levels |
| *KBTBD6* | 1 | Aspartate aminotransferase levels |
| *NAA16* | 0 | Aspartate aminotransferase levels |
| *OR7E36P* | 0 | Aspartate aminotransferase levels |
| *RGCC* | 0 | Aspartate aminotransferase levels |
| *VWA8* | 0 | Aspartate aminotransferase levels |
| *DGKH* | 2 | Type 2 diabetes |
| *TPT1-AS1* | 0 | Type 2 diabetes |
| *COG3* | 1 | Type 2 diabetes |
| *No data avaiable about FAM194B in this dataset!* | NA | Type 2 diabetes |
| *SIAH3* | 1 | Type 2 diabetes |
| *ZC3H13* | 1 | Type 2 diabetes |
| *CPB2* | 0 | Type 2 diabetes |
| *LCP1* | 3 | Type 2 diabetes |
| *LRRC63* | 0 | Type 2 diabetes |
| *SCFD1* | 2 | Type 2 diabetes |
| *COCH* | 1 | Type 2 diabetes |
| *STRN3* | 2 | Type 2 diabetes |
| *AP4S1* | 1 | Type 2 diabetes |
| *HEATR5A* | 1 | Lymphocyte counts |
| *NPAS3* | 0 | Lymphocyte counts |
| *EGLN3* | 1 | Lymphocyte counts |
| *SPTSSA* | 2 | Lymphocyte counts |
| *AKAP11* | 1 | Lymphocyte counts |
| *TNFSF11* | 2 | Lymphocyte counts |
| *EPSTI1* | 0 | Hematocrit |
| *DNAJC15* | 2 | Hematocrit |
| *ENOX1* | 1 | Hematocrit |
| *LACC1* | 1 | Hematocrit |
| *LINC00284* | 0 | Hematocrit |
| *SMIM2* | 0 | Hematocrit |
| *SERP2* | 0 | Hematocrit |
| *TSC22D1* | 1 | Oral cavity cancer |
| *LINC00330* | 0 | Oropharynx cancer |
| *NUFIP1* | 1 | Hypopharyngeal or laryngeal cancer |
| *GTF2F2* | 2 | Head and neck squamous cell carcinoma |
| *SERPINE3* | 0 | Sudden cardiac arrest in coronary artery disease |
| *INTS6* | 1 | Type 2 diabetes |
| *WDFY2* | 1 | Type 2 diabetes |
| *DHRS12* | 1 | Type 2 diabetes |
| *ATP7B* | 1 | Type 2 diabetes |
| *TPTE2P2* | 0 | Type 2 diabetes |
| *THSD1* | 3 | Type 2 diabetes |
| *VPS36* | 2 | Type 2 diabetes |
| *PCDH8* | 0 | Type 2 diabetes |
| *OLFM4* | 0 | Myocardial infarction |
| *LINC01065* | 0 | Myocardial infarction |
| *No data avaiable about AL450423.1 in this dataset!* | NA | Myocardial infarction |
| *LINC00558* | 0 | Myocardial infarction |
| *LINC00458* | 0 | Myocardial infarction |
| *TGFB1I1* | 3 | Myocardial infarction |
| *SLC5A2* | 0 | Myocardial infarction |
| *C16orf58* | NA | Myocardial infarction |
| *No data avaiable about RP11-452L6.7 in this dataset!* | NA | Myocardial infarction |
| *VN1R64P* | 0 | Lymphocyte counts |
| *No data avaiable about RP11-1166P10.6 in this dataset!* | NA | Lymphocyte counts |
| *KLF8P1* | 0 | Lymphocyte counts |
| *CBLN1* | 2 | Lymphocyte counts |
| *C16orf78* | NA | Lymphocyte counts |
| *ZNF423* | 0 | Hematocrit |
| *No data avaiable about RP11-429P3.3 in this dataset!* | NA | Hematocrit |
| *HEATR3* | 0 | Hematocrit |
| *PAPD5* | 0 | Hematocrit |
| *ADCY7* | 1 | Hematocrit |
| *BRD7* | 0 | Hematocrit |
| *NKD1* | 1 | Hematocrit |
| *No data avaiable about RP11-401P9.5 in this dataset!* | NA | Male-pattern baldness |
| *SNX20* | 1 | Male-pattern baldness |
| *NOD2* | 0 | Male-pattern baldness |
| *No data avaiable about AC022816.2 in this dataset!* | NA | Male-pattern baldness |
| *RPL23AP76* | 0 | Male-pattern baldness |
| *CDRT7* | 1 | Male-pattern baldness |
| *PMP22* | 3 | Male-pattern baldness |
| *CDRT4* | 0 | Male-pattern baldness |
| *ADORA2B* | 3 | Male-pattern baldness |
| *ZSWIM7* | 1 | Male-pattern baldness |
| *NCOR1* | 2 | Male-pattern baldness |
| *PIEZO2* | 1 | Male-pattern baldness |
| *GNAL* | 2 | Male-pattern baldness |
| *CHMP1B* | 1 | Male-pattern baldness |
| *ZNF665* | 0 | Male-pattern baldness |
| *VN1R4* | 1 | Male-pattern baldness |
| *MIR526B* | 0 | Male-pattern baldness |
| *BPIFB1* | 0 | Low density lipoprotein cholesterol levels |
| *CDK5RAP1* | 2 | Lymphocyte counts |
| *SNTA1* | 3 | Lymphocyte counts |
| *CBFA2T2* | 0 | Lymphocyte counts |
| *E2F1* | 2 | Lymphocyte counts |
| *PXMP4* | 2 | Lymphocyte counts |
| *ZNF341* | 1 | Lymphocyte counts |
| *CHMP4B* | 1 | Lymphocyte counts |
| *TPM3P2* | 0 | Lymphocyte counts |
| *RALY* | 1 | Lymphocyte counts |
| *EIF2S2* | 3 | Lymphocyte counts |
| *ASIP* | 0 | Lymphocyte counts |
| *AHCYL2* | 1 | Lymphocyte counts |
| *ITCH* | 2 | Lymphocyte counts |
| *DYNLRB1* | 0 | Lymphocyte counts |
| *PIGU* | 2 | Lymphocyte counts |
| *RNU7-144P* | 0 | Lymphocyte counts |
| *PREX1* | 0 | Lymphocyte counts |
| *ARFGEF2* | 0 | Lymphocyte counts |
| *CSE1L* | 2 | Iron status biomarkers (total iron binding capacity) |
| *STAU1* | 3 | Iron status biomarkers (iron levels) |
| *DDX27* | 2 | Iron status biomarkers (transferrin saturation) |
| *ZNFX1* | 0 | Iron status biomarkers (transferrin saturation) |
| *ZFAS1* | 0 | Iron status biomarkers (transferrin saturation) |
| *KCNB1* | 0 | Iron status biomarkers (transferrin saturation) |
| *RBM38* | 0 | Blood protein levels |
| *CTCFL* | 1 | Blood protein levels |
| *PCK1* | 3 | Blood protein levels |
| *ZBP1* | 1 | Blood protein levels |
| *PMEPA1* | 0 | Blood protein levels |
| *C20orf85* | NA | Blood protein levels |
| *ANKRD60* | 0 | Blood protein levels |
| *PPP4R1L* | 0 | Blood protein levels |
| *RAB22A* | 1 | Blood protein levels |
| *APCDD1L* | 0 | Alanine aminotransferase levels |
| *APCDD1L-AS1* | 0 | Alanine aminotransferase levels |
| *RNPEPL1* | 1 | Alanine aminotransferase levels |
| *GNAS-AS1* | 1 | Alanine aminotransferase levels |
| *GNAS-AS1* | 1 | Alanine aminotransferase levels |
| *NELFCD* | 0 | Alanine aminotransferase levels |
| *TUBB1* | 2 | Alanine aminotransferase levels |
| *ATP5E* | 0 | Alanine aminotransferase levels |
| *No data avaiable about SLMO2 in this dataset!* | NA | Alanine aminotransferase levels |
| *ZNF831* | 1 | Alanine aminotransferase levels |
| *EDN3* | 2 | Alanine aminotransferase levels |
| *RGS19* | 3 | Alanine aminotransferase levels |
| *OPRL1* | 3 | Alanine aminotransferase levels |
| *NPBWR2* | 2 | Alanine aminotransferase levels |
| *MYT1L* | 4 | Alanine aminotransferase levels |
| *PCMTD2* | 2 | Gut microbiota relative abundance (Oscillospira) |
| *MIR3156-3* | 0 | Gut microbiota relative abundance (Ruminococcus belonging to family Lachnospiraceae) |
| *FAM227A* | 1 | Gut microbiota relative abundance (Ruminococcus belonging to family Lachnospiraceae) |
| *TOMM22* | 1 | Gut microbiota relative abundance (unassigned genus belonging to family Clostridiales) |
| *JOSD1* | 0 | Type 2 diabetes |
| *GTPBP1* | 2 | Type 2 diabetes |
| *SUN2* | 1 | Type 2 diabetes |
| *NPTXR* | 0 | Type 2 diabetes |
| *CBX6* | 0 | Type 2 diabetes |
| *APOBEC3A* | 1 | Type 2 diabetes |
| *APOBEC3G* | 0 | Type 2 diabetes |
| *CBX7* | 1 | Lymphocyte counts |
| *No data avaiable about AL031590.1 in this dataset!* | NA | Lymphocyte counts |
| *MRPL33* | 2 | Lymphocyte counts |
| *SYNGR1* | 2 | Lymphocyte counts |
| *TAB1* | 2 | Lymphocyte counts |
| *MGAT3* | 2 | Lymphocyte counts |
| *MIEF1* | 0 | Rapid automised naming of pictures |
| *RPS19BP1* | 2 | Rapid automised naming of digits |
| *CACNA1I* | 2 | Short-term memory (digit-span task) |
| *ENTHD1* | 0 | Rapid automised naming of pictures |
| *SYTL5* | 0 | Rapid automised naming of pictures |
| *MIR548AJ2* | 1 | Rapid automised naming of digits |
| *RNU6-330P* | 0 | Rapid automised naming of pictures |
| *KIAA2022* | 1 | Rapid automised naming of pictures |
| *ABCB7* | 0 | Rapid automised naming of digits |
| *HMGN5* | 0 | Non-word reading |
| *POU3F4* | 2 | Non-word reading |
| *MIR548I4* | 0 | Factor VII activity |
| *HDX* | 1 | Factor VII activity |
| *CRB1* | 0 | Factor VII activity |
| *PCDH20* | 2 | Factor VII activity |
| *HIGD1AP2* | 0 | Factor VII activity |
| *PTMAP5* | 0 | Male-pattern baldness |
| *GYG1P2* | 0 | Male-pattern baldness |
| *No data avaiable about RP11-464I4.1 in this dataset!* | NA | Male-pattern baldness |
| *SLITRK1* | 3 | Male-pattern baldness |
| *LINC00333* | 0 | Male-pattern baldness |
| *SLITRK6* | 1 | Male-pattern baldness |
| *TXNL1P1* | 0 | Gut microbiota relative abundance (unclassified genus belonging to family Erysipelotrichaceae) |
| *MIR4500HG* | 1 | Gut microbiota relative abundance (unclassified genus belonging to family Erysipelotrichaceae) |
| *SLITRK5* | 2 | Gut microbiota alpha diversity (Chao1 index) |
| *RPL29P29* | 0 | Gut microbiota alpha diversity (Chao1 index) |
| *LINC00433* | 1 | Gut microbiota alpha diversity (Chao1 index) |
| *LINC00560* | 0 | Gut microbiota alpha diversity (Chao1 index) |
| *LINC00410* | 0 | Gut microbiota alpha diversity (Chao1 index) |
| *MIR17HG* | 0 | Gut microbiota alpha diversity (Chao1 index) |
| *GPC5* | 3 | Gut microbiota alpha diversity (Chao1 index) |
| *GPC6* | 2 | Gut microbiota alpha diversity (PD_whole_tree index) |
| *UGGT2* | 1 | Type 2 diabetes |
| *HS6ST3* | 2 | Type 2 diabetes |
| *LINC00359* | 0 | Type 2 diabetes |
| *MBNL2* | 2 | Type 2 diabetes |
| *RAP2A* | 1 | Type 2 diabetes |
| *IPO5* | 0 | Type 2 diabetes |
| *FARP1* | 2 | Type 2 diabetes |
| *RNF113B* | 1 | Type 2 diabetes |
| *STK24* | 1 | Lymphocyte counts |
| *SLC15A1* | 0 | Lymphocyte counts |
| *DOCK9* | 0 | Lymphocyte counts |
| *DOCK9-AS2* | 0 | Lymphocyte counts |
| *UBAC2* | 0 | Lymphocyte counts |
| *LINC00911* | 0 | Lymphocyte counts |
| *No data avaiable about RP11-497E19.2 in this dataset!* | NA | Lymphocyte counts |
| *FLRT2* | 2 | Lymphocyte counts |
| *No data avaiable about RP11-322L20.1 in this dataset!* | NA | Hemoglobin |
| *No data avaiable about RP11-594C13.1 in this dataset!* | NA | Hemoglobin |
| *GALC* | 2 | Hemoglobin |
| *GLRX5* | 1 | Hemoglobin |
| *TCL6* | 1 | Hemoglobin |
| *TCL1A* | 1 | Hemoglobin |
| *No data avaiable about RP11-164H13.1 in this dataset!* | NA | Eosinophil percentage of white cells |
| *No data avaiable about LINC00617 in this dataset!* | NA | Eosinophil percentage of white cells |
| *C14orf132* | NA | Eosinophil percentage of white cells |
| *BDKRB2* | 2 | Eosinophil percentage of white cells |
| *BDKRB1* | 2 | Eosinophil percentage of white cells |
| *BCL11B* | 2 | Eosinophil percentage of white cells |
| *No data avaiable about CTD-2200A16.1 in this dataset!* | NA | Eosinophil percentage of white cells |
| *SETD3* | 0 | Eosinophil percentage of white cells |
| *CCDC85C* | 0 | Hemoglobin concentration |
| *HHIPL1* | 1 | Hemoglobin concentration |
| *GGACT* | 0 | Hemoglobin concentration |
| *TMTC4* | 3 | Hemoglobin concentration |
| *NALCN-AS1* | 0 | Hemoglobin concentration |
| *NALCN-AS1* | 0 | Hemoglobin concentration |
| *ITGBL1* | 3 | Hemoglobin concentration |
| *FGF14* | 3 | Hemoglobin concentration |
| *FGF14-IT1* | 0 | Hemoglobin concentration |
| *TPP2* | 2 | Hemoglobin concentration |
| *ARGLU1* | 1 | White matter microstructure (fractional anisotropy) |
| *FAM155A* | 2 | White matter microstructure (fractional anisotropy) |
| *MYO16* | 1 | White matter microstructure (fractional anisotropy) |
| *LINC00676* | 0 | White matter microstructure (fractional anisotropy) |
| *IRS2* | 1 | White matter microstructure (fractional anisotropy) |
| *COL4A1* | 1 | White matter microstructure (fractional anisotropy) |
| *COL4A2* | 3 | White matter microstructure (fractional anisotropy) |
| *RAB20* | 0 | White matter microstructure (fractional anisotropy) |
| *CARS2* | 0 | Nonalcoholic fatty liver disease |
| *ING1* | 1 | Nonalcoholic fatty liver disease |
| *LINC00346* | 0 | Nonalcoholic fatty liver disease |
| *ANKRD10* | 1 | Nonalcoholic fatty liver disease |
| *OTUD7A* | 1 | Percent liver fat |
| *TEX29* | 0 | Waist-to-hip ratio adjusted for BMI |
| *No data avaiable about RP11-65D24.2 in this dataset!* | NA | Waist-to-hip ratio adjusted for BMI |
| *RALBP1* | 2 | Waist-to-hip ratio adjusted for BMI |
| *No data avaiable about GAREM in this dataset!* | NA | Waist-to-hip ratio adjusted for BMI |
| *MEP1B* | 2 | Waist-to-hip ratio adjusted for BMI |
| *ASXL3* | 1 | Waist-to-hip ratio adjusted for BMI |
| *NOL4* | 3 | Waist-to-hip ratio adjusted for BMI |
| *DTNA* | 3 | Waist-to-hip ratio adjusted for BMI |
| *MAPRE2* | 2 | Waist-to-hip ratio adjusted for BMI |
| *LINGO3* | 0 | Hepatocyte growth factor levels |
| *LSM7* | 3 | Hepatocyte growth factor levels |
| *GPIHBP1* | 2 | PR interval |
| *ZNF599* | 1 | PR interval |
| *SCN1B* | 1 | Hemoglobin concentration |
| *HPN* | 0 | Hemoglobin concentration |
| *HPN-AS1* | 0 | Hemoglobin concentration |
| *IGFL1* | 1 | Hemoglobin concentration |
| *No data avaiable about AL136302.1 in this dataset!* | NA | Hemoglobin concentration |
| *SOX1* | 0 | Hemoglobin concentration |
| *ARHGEF40* | 2 | Alcohol consumption (drinks per week) (MTAG) |
| *ZNF219* | 1 | Alcohol consumption (drinks per week) (MTAG) |
| *OR5AU1* | 0 | Alcohol consumption (drinks per week) (MTAG) |
| *HNRNPC* | 1 | Alcohol consumption (drinks per week) (MTAG) |
| *RPGRIP1* | 1 | Alcohol consumption (drinks per week) (MTAG) |
| *SALL2* | 3 | Alcohol consumption (drinks per week) (MTAG) |
| *NPIPB9* | 0 | Alcohol consumption (drinks per week) (MTAG) |
| *ATXN2L* | 1 | White matter microstructure (fractional anisotropy) |
| *TUFM* | 1 | Diffuse plaques (SNP x SNP interaction) |
| *SH2B1* | 0 | Diffuse plaques (SNP x SNP interaction) |
| *ATP2A1* | 1 | Diffuse plaques (SNP x SNP interaction) |
| *RABEP2* | 1 | Diffuse plaques (SNP x SNP interaction) |
| *CD19* | 1 | Diffuse plaques (SNP x SNP interaction) |
| *NFATC2IP* | 0 | Vaginal microbiome composition (L. iners) |
| *SPNS1* | 1 | Vaginal microbiome composition (L. iners) |
| *PRR14* | 0 | Vaginal microbiome composition (G. vaginalis) |
| *SRCAP* | 0 | Vaginal microbiome composition (Shannon diversity index) |
| *No data avaiable about RP11-2C24.5 in this dataset!* | NA | Vaginal microbiome composition (Shannon diversity index) |
| *BCL7C* | 1 | Height |
| *AHCTF1P1* | 0 | PR interval |
| *CTF2P* | 0 | PR interval |
| *FBXL19* | 2 | PR interval |
| *ORAI3* | 0 | PR interval |
| *SETD1A* | 1 | PR interval |
| *STX1B* | 1 | PR interval |
| *STX4* | 3 | PR interval |
| *ZNF646* | 1 | PR interval |
| *PRSS53* | 0 | PR interval |
| *VKORC1* | 2 | Hemoglobin concentration |
| *BCKDK* | 1 | Hemoglobin concentration |
| *FUS* | 3 | Hemoglobin concentration |
| *PYCARD* | 3 | Hemoglobin concentration |
| *PSKH1* | 1 | Hemoglobin concentration |
| *LCAT* | 3 | Hemoglobin concentration |
| *DPEP2* | 1 | circulating leptin levels adjusted for BMI |
| *NFATC3* | 2 | circulating leptin levels adjusted for BMI |
| *NOB1* | 3 | circulating leptin levels adjusted for BMI |
| *WWP2* | 0 | circulating leptin levels adjusted for BMI |
| *PDXDC2P* | 1 | circulating leptin levels adjusted for BMI |
| *PDPR* | 2 | circulating leptin levels adjusted for BMI |
| *CLEC18C* | 0 | circulating leptin levels adjusted for BMI |
| *CELF4* | 2 | circulating leptin levels adjusted for BMI |
| *MIR4318* | 1 | circulating leptin levels adjusted for BMI |
| *No data avaiable about RP11-19F9.1 in this dataset!* | NA | circulating leptin levels adjusted for BMI |
| *No data avaiable about LINC00669 in this dataset!* | NA | circulating leptin levels adjusted for BMI |
| *RPL7AP66* | 0 | circulating leptin levels adjusted for BMI |
| *No data avaiable about RNU7-145P in this dataset!* | NA | circulating leptin levels adjusted for BMI |
| *PIK3C3* | 2 | circulating leptin levels adjusted for BMI |
| *LINC00907* | 0 | circulating leptin levels adjusted for BMI |
| *LRIT2* | 1 | circulating leptin levels |
| *TMEM147* | 1 | circulating leptin levels |
| *ATP4A* | 0 | circulating leptin levels |
| *IGFLR1* | 1 | circulating leptin levels |
| *ARHGAP33* | 1 | circulating leptin levels |
| *ZNF600* | 0 | circulating leptin levels |
| *ZNF281* | 1 | circulating leptin levels |
| *ZNF320* | 1 | circulating leptin levels |
| *ZNF888* | 0 | circulating leptin levels |
| *ZNF160* | 1 | circulating leptin levels |
| *GMPR2* | 1 | circulating leptin levels |
| *No data avaiable about CTD-2591A6.2 in this dataset!* | NA | circulating leptin levels |
| *FOXG1* | 0 | circulating leptin levels |
| *No data avaiable about C14ORF23 in this dataset!* | NA | circulating leptin levels |
| *No data avaiable about RP11-562L8.1 in this dataset!* | NA | circulating leptin levels |
| *RNU11-5P* | 0 | circulating leptin levels adjusted for BMI |
| *PRKD1* | 2 | circulating leptin levels adjusted for BMI |
| *NUBPL* | 1 | Diffuse plaques (SNP x SNP interaction) |
| *PLCB2* | 2 | Diffuse plaques (SNP x SNP interaction) |
| *DISP2* | 1 | Diffuse plaques (SNP x SNP interaction) |
| *KNSTRN* | 0 | Diffuse plaques (SNP x SNP interaction) |
| *IVD* | 2 | Diffuse plaques (SNP x SNP interaction) |
| *BAHD1* | 1 | Diffuse plaques (SNP x SNP interaction) |
| *CASC5* | 2 | Spontaneous coronary artery dissection |
| *RAD51* | 1 | Spontaneous coronary artery dissection |
| *GCHFR* | 0 | Hemoglobin concentration |
| *DNAJC17* | 1 | Hemoglobin concentration |
| *SPINT1* | 3 | Hemoglobin concentration |
| *VPS18* | 1 | Hemoglobin concentration |
| *No data avaiable about RP11-540O11.1 in this dataset!* | NA | Hemoglobin concentration |
| *DLL4* | 2 | Hemoglobin concentration |
| *CHAC1* | 0 | Hemoglobin concentration |
| *INO80* | 0 | Hemoglobin concentration |
| *GALNT16* | 0 | Hemoglobin concentration |
| *ERH* | 1 | Hemoglobin concentration |
| *SLC39A9* | 0 | Hemoglobin concentration |
| *PLEKHD1* | 0 | Hemoglobin concentration |
| *No data avaiable about KIAA0247 in this dataset!* | NA | Hemoglobin concentration |
| *SRSF5* | 1 | Hemoglobin concentration |
| *SLC10A1* | 1 | Autoimmune traits |
| *AK7* | 0 | Mental disorder (without autoimmune disease) |
| *PAPOLA* | 1 | Mental disorder (without autoimmune disease) |
| *No data avaiable about RP11-61O1.1 in this dataset!* | NA | Autoimmune disease and mental disorder |
| *VRK1* | 0 | Hematocrit |
| *LINC00618* | 0 | Hematocrit |
| *IGHVII-22-1* | 0 | Hematocrit |
| *HOMER2P2* | 0 | Hematocrit |
| *No data avaiable about RP11-204N11.1 in this dataset!* | NA | Hematocrit |
| *No data avaiable about C14ORF64 in this dataset!* | NA | Hematocrit |
| *C14orf177* | NA | Serum uric acid levels |
| *No data avaiable about RP11-566J3.2 in this dataset!* | NA | Serum uric acid levels |
| *DLK1* | 1 | Serum uric acid levels |
| *MEG3* | 1 | Serum uric acid levels |
| *RTL1* | 1 | Serum uric acid levels |
| *No data avaiable about AL117190.3 in this dataset!* | NA | Serum uric acid levels |
| *MEG8* | 0 | Serum uric acid levels |
| *SIVA1* | 1 | Waist-to-hip ratio adjusted for BMI |
| *IGHVII-33-1* | 0 | Waist-to-hip ratio adjusted for BMI |
| *IGHV4-39* | 0 | Waist-to-hip ratio adjusted for BMI |
| *HOMER2P1* | 0 | Waist-to-hip ratio adjusted for BMI |
| *IGHV3-47* | 0 | Type 2 diabetes |
| *FAM189A1* | 1 | Type 2 diabetes |
| *ARHGAP11B* | 0 | Type 2 diabetes |
| *TJP1* | 2 | Allergic disease (asthma |
| *FAN1* | 0 | hay fever and/or eczema) (age of onset) |
| *TRPM1* | 0 | hay fever and/or eczema) (age of onset) |
| *KLF13* | 3 | Allergic disease (asthma |
| *GJD2* | 0 | hay fever and/or eczema) (age of onset) |
| *RFX7* | 1 | hay fever and/or eczema) (age of onset) |
| *BNIP2* | 1 | Allergic disease (asthma |
| *LINGO1* | 2 | Allergic disease (asthma |
| *ANKRD34C* | 0 | hay fever and/or eczema) (age of onset) |
| *TMED3* | 2 | Allergic disease (asthma |
| *KIAA1024* | 1 | Allergic disease (asthma |
| *MTHFS* | 1 | Allergic disease (asthma |
| *ST20* | 0 | hay fever and/or eczema) (age of onset) |
| *No data avaiable about C15ORF37 in this dataset!* | NA | Allergic disease (asthma |
| *BCL2A1* | 1 | Allergic disease (asthma |
| *ZFAND6* | 2 | Allergic disease (asthma |
| *PAFAH1B2* | 1 | hay fever and/or eczema) (age of onset) |
| *LINC00927* | 1 | Allergic disease (asthma |
| *ARNT2* | 2 | hay fever and/or eczema) (age of onset) |
| *No data avaiable about RP11-379K22.2 in this dataset!* | NA | Allergic disease (asthma |
| *ABHD17C* | 0 | hay fever and/or eczema) (age of onset) |
| *No data avaiable about KIAA1199 in this dataset!* | NA | Allergic disease (asthma |
| *MESDC2* | 2 | hay fever and/or eczema) (age of onset) |
| *No data avaiable about C15ORF26 in this dataset!* | NA | Allergic disease (asthma |
| *IL16* | 1 | hay fever and/or eczema) (age of onset) |
| *XYLT1* | 2 | Allergic disease (asthma |
| *NPIPA8* | 0 | hay fever and/or eczema) (age of onset) |
| *NOMO2* | 0 | Allergic disease (asthma |
| *RPS15A* | 3 | Allergic disease (asthma |
| *No data avaiable about RP11-114H24.4 in this dataset!* | NA | hay fever and/or eczema) (age of onset) |
| *TBC1D2B* | 3 | Allergic disease (asthma |
| *SH2D7* | 0 | Allergic disease (asthma |
| *ACSBG1* | 3 | hay fever and/or eczema) (age of onset) |
| *No data avaiable about RP11-762H8.3 in this dataset!* | NA | Allergic disease (asthma |
| *DNAJA4* | 3 | hay fever and/or eczema) (age of onset) |
| *WDR61* | 1 | Allergic disease (asthma |
| *IREB2* | 2 | hay fever and/or eczema) (age of onset) |
| *PSMA4* | 3 | hay fever and/or eczema) (age of onset) |
| *CHRNA5* | 2 | hay fever and/or eczema) (age of onset) |
| *CHRNA3* | 2 | Allergic disease (asthma |
| *CHRNB4* | 0 | hay fever and/or eczema) (multivariate analysis) |
| *ADAMTS7* | 1 | Allergic disease (asthma |
| *MORF4L1* | 2 | Allergic disease (asthma |
| *CTSH* | 2 | Allergic disease (asthma |
| *RASGRF1* | 2 | hay fever and/or eczema) (multivariate analysis) |
| *UBE2Q2P1* | 1 | Allergic disease (asthma |
| *ZSCAN2* | 2 | hay fever and/or eczema) (multivariate analysis) |
| *WDR73* | 1 | Allergic disease (asthma |
| *NMBR* | 4 | hay fever and/or eczema) (multivariate analysis) |
| *SEC11A* | 0 | Allergic disease (asthma |
| *ZNF592* | 1 | hay fever and/or eczema) (multivariate analysis) |
| *ALPK3* | 3 | hay fever and/or eczema) (multivariate analysis) |
| *SLC28A1* | 0 | Allergic disease (asthma |
| *PDE8A* | 2 | hay fever and/or eczema) (multivariate analysis) |
| *AGBL1* | 2 | hay fever and/or eczema) (multivariate analysis) |
| *NTRK3* | 2 | Allergic disease (asthma |
| *MRPS11* | 0 | hay fever and/or eczema) (multivariate analysis) |
| *DET1* | 1 | hay fever and/or eczema) (multivariate analysis) |
| *MIR1179* | 0 | hay fever and/or eczema) (multivariate analysis) |
| *PMM2* | 2 | Allergic disease (asthma |
| *CARHSP1* | 2 | Allergic disease (asthma |
| *USP7* | 1 | hay fever and/or eczema) (multivariate analysis) |
| *C16orf72* | NA | hay fever and/or eczema) (multivariate analysis) |
| *RPL21P119* | 0 | Allergic disease (asthma |
| *No data avaiable about RP11-297M9.1 in this dataset!* | NA | hay fever and/or eczema) (multivariate analysis) |
| *GTF3C1* | 2 | Allergic disease (asthma |
| *KIAA0556* | 2 | hay fever and/or eczema) (multivariate analysis) |
| *GSG1L* | 1 | Adolescent idiopathic scoliosis |
| *XPO6* | 1 | Waist-to-hip ratio adjusted for BMI |
| *SBK1* | 1 | Waist-to-hip ratio adjusted for BMI |
| *NPIPB6* | 0 | Waist-to-hip ratio adjusted for BMI |
| *CLN3* | 3 | Waist-to-hip ratio adjusted for BMI |
| *APOBR* | 1 | Waist-to-hip ratio adjusted for BMI |
| *SNX29P2* | 0 | Waist-to-hip ratio adjusted for BMI |
| *NPIPB11* | 0 | Waist-to-hip ratio adjusted for BMI |
| *No data avaiable about RP11-345J4.8 in this dataset!* | NA | Waist-to-hip ratio adjusted for BMI |
| *SLC7A5P1* | 0 | hay fever and/or eczema) (multivariate analysis) |
| *CA5AP1* | 0 | PR interval |
| *PIK3R5* | 2 | PR interval |
| *CNTN1* | 4 | PR interval |
| *STX8* | 1 | PR interval |
| *USP43* | 2 | PR interval |
| *GLP2R* | 3 | PR interval |
| *GAS7* | 1 | PR interval |
| *PHB* | 2 | Eosinophil percentage of white cells |
| *NGFR* | 2 | Eosinophil percentage of white cells |
| *FAM117A* | 1 | Eosinophil percentage of white cells |
| *KAT7* | 1 | Eosinophil percentage of white cells |
| *DLX4* | 1 | Eosinophil percentage of white cells |
| *VMP1* | 1 | Eosinophil percentage of white cells |
| *RNU6-450P* | 0 | Eosinophil percentage of white cells |
| *TUBD1* | 2 | Eosinophil percentage of white cells |
| *RPS6KB1* | 2 | Hemoglobin concentration |
| *ICOSLG* | 1 | Hemoglobin concentration |
| *AIRE* | 1 | Hemoglobin concentration |
| *PFKL* | 2 | Hemoglobin concentration |
| *C21orf2* | NA | Hemoglobin concentration |
| *TRPM2* | 1 | Hemoglobin concentration |
| *LRRC3* | 2 | Hemoglobin concentration |
| *TSPEAR* | 0 | Hemoglobin concentration |
| *RBFOX2* | 0 | Hemoglobin concentration |
| *APOL3* | 1 | Hemoglobin concentration |
| *APOL2* | 0 | Hemoglobin concentration |
| *APOL1* | 1 | Hemoglobin concentration |
| *MYH9* | 2 | Hemoglobin concentration |
| *CACNG2* | 2 | Hemoglobin concentration |
| *PVALB* | 2 | Autism spectrum disorder (MTAG) |
| *NCF4* | 3 | Autism spectrum disorder (MTAG) |
| *CSF2RB* | 2 | Autism spectrum disorder (MTAG) |
| *MPST* | 2 | Autism spectrum disorder (MTAG) |
| *KCTD17* | 1 | Autism spectrum disorder (MTAG) |
| *TMPRSS6* | 1 | Depressive symptoms (MTAG) |
| *IL2RB* | 1 | Depressive symptoms (MTAG) |
| *C1QTNF6* | 0 | Depressive symptoms (MTAG) |
| *RAC2* | 2 | Depressive symptoms (MTAG) |
| *CYTH4* | 1 | Depressive symptoms (MTAG) |
| *CARD10* | 2 | PR interval |
| *LGALS2* | 1 | PR interval |
| *GGA1* | 3 | Depressive symptoms (MTAG) |
| *TRIOBP* | 2 | Depressive symptoms (MTAG) |
| *H1F0* | 3 | Depressive symptoms (MTAG) |
| *GCAT* | 1 | Depressive symptoms (MTAG) |
| *CLCN5* | 1 | Depressive symptoms (MTAG) |
| *DGKK* | 1 | Depressive symptoms (MTAG) |
| *SHROOM4* | 1 | Depressive symptoms (MTAG) |
| *MORF4L2* | 3 | Depressive symptoms (MTAG) |
| *NRK* | 0 | Liver fibrosis and steatohepatitis severity (MRI cT1 measure) |
| *SERPINA7* | 0 | Liver fibrosis and steatohepatitis severity (MRI cT1 measure) |
| *CXorf57* | NA | Liver fibrosis and steatohepatitis severity (MRI cT1 measure) |
| *MIR548AN* | 0 | Liver fibrosis and steatohepatitis severity (MRI cT1 measure) |
| *RNF128* | 0 | Liver fibrosis and steatohepatitis severity (MRI cT1 measure) |
| *MORC4* | 1 | General cognitive ability |
| *NUP62CL* | 1 | General cognitive ability |
| *PIH1D3* | 0 | General cognitive ability |
| *COL4A6* | 2 | General cognitive ability |
| *FAM58A* | 0 | Eosinophil percentage of white cells |
| *DUSP9* | 1 | Eosinophil percentage of white cells |
| *ARHGAP40* | 0 | Eosinophil percentage of white cells |
| *OPN1LW* | 0 | Eosinophil percentage of white cells |
| *TKTL1* | 1 | Eosinophil percentage of white cells |
| *FLNA* | 2 | Eosinophil percentage of white cells |
| *EMD* | 1 | Eosinophil percentage of white cells |
| *UBL4A* | 1 | Eosinophil percentage of white cells |
| *TBL1Y* | 1 | Eosinophil percentage of white cells |
| *IDH2* | 3 | Eosinophil percentage of white cells |
| *MYO18A* | 2 | Eosinophil percentage of white cells |
| *TWF1P1* | 0 | Eosinophil percentage of white cells |
| *CRYBA1* | 1 | Eosinophil percentage of white cells |
| *NUFIP2* | 0 | Eosinophil percentage of white cells |
| *No data avaiable about RP11-296K13.4 in this dataset!* | NA | Eosinophil percentage of white cells |
| *TAOK1* | 2 | Eosinophil percentage of white cells |
| *ABHD15* | 1 | Eosinophil percentage of white cells |
| *GIT1* | 2 | Eosinophil percentage of white cells |
| *ANKRD13B* | 1 | Eosinophil percentage of white cells |
| *CORO6* | 2 | Hemoglobin concentration |
| *SSH2* | 2 | Hemoglobin concentration |
| *EFCAB5* | 0 | Hemoglobin concentration |
| *SEMA4B* | 2 | Hemoglobin concentration |
| *NGRN* | 1 | Hemoglobin concentration |
| *ZNF774* | 1 | Hemoglobin concentration |
| *CRTC3* | 2 | Hemoglobin concentration |
| *SPATA8* | 2 | Attention deficit hyperactivity disorder |
| *SLC6A4* | 0 | Attention deficit hyperactivity disorder |
| *LINC00923* | 0 | Attention deficit hyperactivity disorder |
| *ARRDC4* | 3 | Attention deficit hyperactivity disorder (MTAG) |
| *FAM169B* | 2 | Attention deficit hyperactivity disorder (MTAG) |
| *XPNPEP2* | 0 | Attention deficit hyperactivity disorder (MTAG) |
| *ZDHHC9* | 1 | Attention deficit hyperactivity disorder (MTAG) |
| *No data avaiable about RP4-537K23.4 in this dataset!* | NA | Attention deficit hyperactivity disorder (MTAG) |
| *BCORL1* | 1 | Autism spectrum disorder |
| *ZNF280C* | 1 | Depressive symptoms |
| *ENOX2* | 1 | Depressive symptoms |
| *No data avaiable about AC144835.1 in this dataset!* | NA | Waist-to-hip ratio adjusted for BMI |
| *MEF2A* | 2 | Waist-to-hip ratio adjusted for BMI |
| *No data avaiable about RP11-35O15.1 in this dataset!* | NA | Waist-to-hip ratio adjusted for BMI |
| *IGF1R* | 2 | Waist-to-hip ratio adjusted for BMI |
| *TTC23L* | 1 | Waist-to-hip ratio adjusted for BMI |
| *PGPEP1L* | 0 | Waist-to-hip ratio adjusted for BMI |
| *SYNM* | 0 | Waist-to-hip ratio adjusted for BMI |
| *LRRC28* | 0 | Diffuse plaques (SNP x SNP interaction) |
| *ADAMTS17* | 1 | Diffuse plaques (SNP x SNP interaction) |
| *CERS3* | 0 | Diffuse plaques (SNP x SNP interaction) |
| *LYSMD4* | 0 | Diffuse plaques (SNP x SNP interaction) |
| *ASB7* | 2 | Diffuse plaques (SNP x SNP interaction) |
| *CHSY1* | 1 | Diffuse plaques (SNP x SNP interaction) |
| *SNRPA1* | 1 | Diffuse plaques (SNP x SNP interaction) |
| *PCSK6* | 1 | Diffuse plaques (SNP x SNP interaction) |
| *LRRK1* | 2 | Diffuse plaques (SNP x SNP interaction) |
| *No data avaiable about RP11-424I19.2 in this dataset!* | NA | Diffuse plaques (SNP x SNP interaction) |
| *FLYWCH1* | 0 | Diffuse plaques (SNP x SNP interaction) |
| *PKMYT1* | 0 | Breast cancer |
| *TMEM106A* | 1 | Breast cancer |
| *LINC00854* | 1 | Invasive breast cancer |
| *MXRA7* | 1 | Breast cancer |
| *JMJD6* | 1 | Breast cancer |
| *MGAT5B* | 1 | Invasive breast cancer |
| *CDIP1* | 0 | Invasive breast cancer |
| *C16orf96* | NA | Invasive breast cancer |
| *UBALD1* | 0 | Breast cancer |
| *GRIN2A* | 2 | Coronary artery disease |
| *CHAF1B* | 3 | Coronary artery disease |
| *IL21R* | 1 | Coronary artery disease |
| *SULT1A1* | 2 | Coronary artery disease |
| *NPIPB8* | 0 | Hemoglobin concentration |
| *No data avaiable about RP11-523L20.2 in this dataset!* | NA | Hemoglobin concentration |
| *ABCC12* | 0 | Hemoglobin concentration |
| *ABCC11* | 1 | Hemoglobin concentration |
| *SIAH1* | 1 | Hemoglobin concentration |
| *N4BP1* | 1 | Hemoglobin concentration |
| *EDC4* | 1 | Hemoglobin concentration |
| *PSMB10* | 2 | Hemoglobin concentration |
| *DUS2* | 0 | Hemoglobin concentration |
| *CDH3* | 3 | Hemoglobin concentration |
| *CDH12* | 4 | Hemoglobin concentration |
| *TANGO6* | 0 | Hemoglobin concentration |
| *HAS3* | 2 | Hemoglobin concentration |
| *SNTB2* | 2 | Hemoglobin concentration |
| *VPS4A* | 2 | Hematocrit |
| *TERF2* | 1 | Hematocrit |
| *CYB5B* | 1 | Hematocrit |
| *NFAT5* | 2 | Hematocrit |
| *NQO1* | 3 | Waist-to-hip ratio adjusted for BMI |
| *AARS* | 2 | Waist-to-hip ratio adjusted for BMI |
| *No data avaiable about AC005152.3 in this dataset!* | NA | Waist-to-hip ratio adjusted for BMI |
| *SOX9* | 2 | Waist-to-hip ratio adjusted for BMI |
| *SLC39A11* | 1 | Waist-to-hip ratio adjusted for BMI |
| *SSTR2* | 2 | Waist-to-hip ratio adjusted for BMI |
| *FAM104A* | 1 | Waist-to-hip ratio adjusted for BMI |
| *ZNF433* | 1 | Waist-to-hip ratio adjusted for BMI |
| *ZNF844* | 0 | Waist-to-hip ratio adjusted for BMI |
| *ZNF788* | 1 | Coronary artery disease |
| *CELSR2* | 2 | Coronary artery disease |
| *USF2* | 1 | Coronary artery disease |
| *HAMP* | 1 | Coronary artery disease |
| *CD226* | 1 | Hematocrit |
| *FFAR2* | 1 | Invasive breast cancer |
| *DMKN* | 1 | Breast cancer (estrogen-receptor negative |
| *GAPDHS* | 1 | Adolescent idiopathic scoliosis |
| *MBTPS1* | 1 | Adolescent idiopathic scoliosis |
| *HSDL1* | 0 | Adolescent idiopathic scoliosis |
| *DNAAF1* | 0 | Adolescent idiopathic scoliosis |
| *TAF1C* | 1 | Adolescent idiopathic scoliosis |
| *ADAD2* | 0 | Adolescent idiopathic scoliosis |
| *KCNG4* | 2 | Adolescent idiopathic scoliosis |
| *WFDC1* | 2 | Adolescent idiopathic scoliosis |
| *ATP2C2* | 0 | Adolescent idiopathic scoliosis |
| *TLDC1* | 0 | Adolescent idiopathic scoliosis |
| *COTL1* | 1 | Adolescent idiopathic scoliosis |
| *GID4* | 0 | Adolescent idiopathic scoliosis |
| *MYO15A* | 0 | Adolescent idiopathic scoliosis |
| *LLGL1* | 1 | Adolescent idiopathic scoliosis |
| *TOP3A* | 1 | Cerebrospinal fluid t-tau:AB1-42 ratio |
| *GFAP* | 3 | Serum uric acid levels |
| *C1QL1* | 0 | PR interval |
| *DCAKD* | 0 | PR interval |
| *NMT1* | 2 | PR interval |
| *PLCD3* | 2 | PR interval |
| *ACBD4* | 1 | PR interval |
| *HEXIM1* | 1 | PR interval |
| *No data avaiable about RP13-890H12.2 in this dataset!* | NA | PR interval |
| *No data avaiable about CTD-2020K17.1 in this dataset!* | NA | Severe COVID-19 infection with respiratory failure (analysis I) |
| *FMNL1* | 2 | Severe COVID-19 infection with respiratory failure (analysis I) |
| *SPATA32* | 0 | Severe COVID-19 infection with respiratory failure (analysis I) |
| *MAP3K14* | 2 | Severe COVID-19 infection with respiratory failure (analysis I) |
| *RNA5SP443* | 0 | Eosinophil percentage of white cells |
| *ARHGAP27* | 0 | Eosinophil percentage of white cells |
| *KANSL1-AS1* | 0 | Eosinophil percentage of white cells |
| *No data avaiable about RP11-259G18.2 in this dataset!* | NA | Eosinophil percentage of white cells |
| *No data avaiable about RP11-259G18.3 in this dataset!* | NA | Eosinophil percentage of white cells |
| *No data avaiable about RP11-259G18.1 in this dataset!* | NA | Eosinophil percentage of white cells |
| *LRRC37A* | 1 | Eosinophil percentage of white cells |
| *NSFP1* | 1 | Eosinophil percentage of white cells |
| *LRRC37A2* | 0 | White matter microstructure (fractional anisotropy) |
| *ARL17A* | 0 | White matter microstructure (fractional anisotropy) |
| *NSFP1* | 1 | White matter microstructure (fractional anisotropy) |
| *WNT3A* | 2 | White matter microstructure (fractional anisotropy) |
| *WNT9B* | 1 | White matter microstructure (fractional anisotropy) |
| *GOSR2* | 1 | White matter microstructure (fractional anisotropy) |
| *No data avaiable about RP11-156P1.3 in this dataset!* | NA | White matter microstructure (fractional anisotropy) |
| *PPP1R1B* | 3 | White matter microstructure (fractional anisotropy) |
| *STARD3* | 1 | White matter microstructure (fractional anisotropy) |
| *PNMT* | 0 | White matter microstructure (fractional anisotropy) |
| *PGAP3* | 1 | White matter microstructure (fractional anisotropy) |
| *ERBB2* | 2 | White matter microstructure (fractional anisotropy) |
| *BAIAP2* | 3 | White matter microstructure (fractional anisotropy) |
| *AATK* | 2 | Waist-to-hip ratio adjusted for BMI |
| *ARHGAP28* | 0 | Waist-to-hip ratio adjusted for BMI |
| *CYB5A* | 0 | Waist-to-hip ratio adjusted for BMI |
| *NFIC* | 3 | Waist-to-hip ratio adjusted for BMI |
| *No data avaiable about C19ORF77 in this dataset!* | NA | Waist-to-hip ratio adjusted for BMI |
| *FZR1* | 2 | Waist-to-hip ratio adjusted for BMI |
| *ARRDC5* | 0 | Waist-to-hip ratio adjusted for BMI |
| *UHRF1BP1* | 1 | Waist-to-hip ratio adjusted for BMI |
| *KDM4B* | 2 | White matter microstructure (fractional anisotropy) |
| *TINCR* | 0 | White matter microstructure (fractional anisotropy) |
| *SAFB2* | 1 | White matter microstructure (fractional anisotropy) |
| *SAFB2* | 1 | White matter microstructure (fractional anisotropy) |
| *LONP1* | 1 | White matter microstructure (fractional anisotropy) |
| *DUS3L* | 2 | White matter microstructure (fractional anisotropy) |
| *NRTN* | 0 | White matter microstructure (fractional anisotropy) |
| *FUT6* | 0 | White matter microstructure (fractional anisotropy) |
| *FUT3* | 0 | White matter microstructure (fractional anisotropy) |
| *FUT5* | 0 | General cognitive ability |
| *NDUFA11* | 1 | General cognitive ability |
| *VMAC* | 0 | General cognitive ability |
| *RANBP3L* | 0 | General cognitive ability |
| *RFX2* | 3 | General cognitive ability |
| *ACSBG2* | 0 | PR interval |
| *MLLT10* | 2 | PR interval |
| *No data avaiable about CTD-2081K17.2 in this dataset!* | NA | PR interval |
| *UQCRFS1* | 0 | PR interval |
| *NUDT19* | 0 | PR interval |
| *TDRD12* | 1 | PR interval |
| *SLC7A9* | 1 | PR interval |
| *CEP89* | 1 | PR interval |
| *No data avaiable about C19ORF40 in this dataset!* | NA | PR interval |
| *RHPN2* | 1 | PR interval |
| *GPATCH1* | 1 | PR interval |
| *No data avaiable about KB-67B5.12 in this dataset!* | NA | PR interval |
| *CCT8L2* | 1 | PR interval |
| *CDC45* | 0 | PR interval |
| *TBX1* | 2 | PR interval |
| *TXNRD2* | 1 | Schizophrenia (MTAG) |
| *COMT* | 1 | Schizophrenia (MTAG) |
| *ARVCF* | 0 | Schizophrenia (MTAG) |
| *TANGO2* | 0 | Schizophrenia (MTAG) |
| *DGCR8* | 1 | Schizophrenia (MTAG) |
| *RTN4R* | 2 | Schizophrenia (MTAG) |
| *SPECC1L* | 0 | Eosinophil percentage of white cells |
| *ADORA2A* | 0 | Eosinophil percentage of white cells |
| *ADORA2A-AS1* | 0 | Eosinophil percentage of white cells |
| *UPB1* | 1 | Eosinophil percentage of white cells |
| *GGT1* | 1 | Eosinophil percentage of white cells |
| *PIWIL3* | 0 | Alcohol consumption (drinks per week) (MTAG) |
| *SGSM1* | 1 | Alcohol consumption (drinks per week) (MTAG) |
| *TMEM211* | 1 | Alcohol consumption (drinks per week) (MTAG) |
| *KIAA1671* | 1 | Alcohol consumption (drinks per week) (MTAG) |
| *ARSE* | 0 | Problematic alcohol use |
| *MXRA5* | 0 | Problematic alcohol use |
| *No data avaiable about AC108683.1 in this dataset!* | NA | Problematic alcohol use |
| *NLGN4X* | 1 | Problematic alcohol use |
| *VCX3B* | 0 | Diffuse plaques (SNP x SNP interaction) |
| *FAM9B* | 0 | Diffuse plaques (SNP x SNP interaction) |
| *TBL1X* | 3 | Diffuse plaques (SNP x SNP interaction) |
| *SHROOM2* | 1 | Diffuse plaques (SNP x SNP interaction) |
| *HCCS* | 0 | Diffuse plaques (SNP x SNP interaction) |
| *ARHGAP6* | 3 | Diffuse plaques (SNP x SNP interaction) |
| *FRMPD4* | 1 | Diffuse plaques (SNP x SNP interaction) |
| *CXorf21* | NA | Diffuse plaques (SNP x SNP interaction) |
| *FTHL17* | 1 | Diffuse plaques (SNP x SNP interaction) |
| *DMD* | 1 | Diffuse plaques (SNP x SNP interaction) |
| *MIR548F5* | 1 | Diffuse plaques (SNP x SNP interaction) |
| *MAGED1* | 2 | Diffuse plaques (SNP x SNP interaction) |
| *STARD8* | 1 | Coronary artery disease |
| *PJA1* | 2 | Coronary artery disease |
| *LINC00269* | 0 | Coronary artery disease |
| *FAM155B* | 0 | Coronary artery disease |
| *EDA* | 0 | Coronary artery disease |
| *DLG3* | 1 | Coronary artery disease |
| *TEX11* | 3 | Coronary artery disease |
| *LINC00629* | 0 | Coronary artery disease |
| *PLAC1* | 0 | Coronary artery disease |
| *KANK2* | 1 | Coronary artery disease |
| *DOCK6* | 2 | Coronary artery disease |
| *No data avaiable about C19ORF80 in this dataset!* | NA | Coronary artery disease |
| *TSPAN16* | 1 | Coronary artery disease |
| *RAB3D* | 2 | Coronary artery disease |
| *No data avaiable about DKFZP761J1410 in this dataset!* | NA | Coronary artery disease |
| *EPOR* | 3 | Eosinophil percentage of white cells |
| *RGL3* | 1 | Eosinophil percentage of white cells |
| *ACP5* | 3 | Eosinophil percentage of white cells |
| *ZNF823* | 1 | Eosinophil percentage of white cells |
| *ZNF441* | 0 | Eosinophil percentage of white cells |
| *ZNF491* | 0 | Eosinophil percentage of white cells |
| *ZNF440* | 1 | Eosinophil percentage of white cells |
| *ZNF439* | 1 | Eosinophil percentage of white cells |
| *ZNF696* | 0 | Eosinophil percentage of white cells |
| *ZNF700* | 1 | Eosinophil percentage of white cells |
| *ZNF763* | 0 | Eosinophil percentage of white cells |
| *PRKCSH* | 1 | Eosinophil percentage of white cells |
| *ELAVL3* | 2 | Eosinophil percentage of white cells |
| *THBD* | 3 | Eosinophil percentage of white cells |
| *CD93* | 1 | Eosinophil percentage of white cells |
| *NXT1* | 1 | Hemoglobin concentration |
| *NAPB* | 3 | Hemoglobin concentration |
| *ZNF90* | 0 | Hemoglobin concentration |
| *ZNF486* | 1 | Hemoglobin concentration |
| *No data avaiable about MIR1270-1 in this dataset!* | NA | Hemoglobin concentration |
| *ZNF626* | 1 | Hemoglobin concentration |
| *ZNF675* | 1 | Hemoglobin concentration |
| *ZNF726* | 1 | Hemoglobin concentration |
| *ZNF254* | 1 | Hemoglobin concentration |
| *LGALS14* | 0 | Hemoglobin concentration |
| *FCGBP* | 1 | Hemoglobin concentration |
| *RNU6-945P* | 0 | Hemoglobin concentration |
| *TMEM150B* | 0 | Hemoglobin concentration |
| *FAM71E2* | 1 | Hemoglobin concentration |
| *IL11* | 1 | Hemoglobin concentration |
| *TMEM190* | 0 | Hemoglobin concentration |
| *ZSCAN5A* | 1 | Hemoglobin concentration |
| *ZNF460* | 1 | Hemoglobin concentration |
| *ZNF304* | 0 | Hemoglobin concentration |
| *ZNF547* | 0 | Hemoglobin concentration |
| *ATRNL1* | 1 | Hemoglobin concentration |
| *ADAM33* | 2 | Hemoglobin concentration |
| *SIGLEC12* | 0 | Hemoglobin concentration |
| *HSPA12B* | 1 | Blood protein levels |
| *C20orf27* | NA | Blood protein levels |
| *LINC00658* | 0 | Blood protein levels |
| *SPEF1* | 3 | Blood protein levels |
| *CDC25B* | 1 | Blood protein levels |
| *MAVS* | 2 | Blood protein levels |
| *PANK2* | 2 | Blood protein levels |
| *RNF24* | 2 | Blood protein levels |
| *No data avaiable about AP000459.7 in this dataset!* | NA | Type 2 diabetes |
| *No data avaiable about AP000470.2 in this dataset!* | NA | Type 2 diabetes |
| *MAP3K7CL* | 0 | Type 2 diabetes |
| *LINC00189* | 1 | Type 2 diabetes |
| *BACH1* | 2 | Type 2 diabetes |
| *IFNAR2* | 3 | Type 2 diabetes |
| *IL10RB* | 1 | Type 2 diabetes |
| *IFNAR1* | 1 | Type 2 diabetes |
| *MIR802* | 0 | Type 2 diabetes |
| *CBR3* | 3 | Type 2 diabetes |
| *MORC3* | 2 | Type 2 diabetes |
| *No data avaiable about AP000695.6 in this dataset!* | NA | Lymphocyte counts |
| *CLDN14* | 1 | Lymphocyte counts |
| *SIM2* | 1 | Lymphocyte counts |
| *HLCS* | 3 | Lymphocyte counts |
| *PSMG1* | 2 | Lymphocyte counts |
| *BRWD1* | 1 | Hematocrit |
| *IGSF5* | 0 | Hematocrit |
| *PCP4* | 1 | Hematocrit |
| *LINC00323* | 1 | Hematocrit |
| *BACE2* | 3 | Hematocrit |
| *FAM3B* | 0 | Adolescent idiopathic scoliosis |
| *HMX2* | 1 | Adolescent idiopathic scoliosis |
| *TMPRSS2* | 1 | Adolescent idiopathic scoliosis |
| *HMGN1* | 2 | Adolescent idiopathic scoliosis |
| *RNF6P1* | 0 | Adolescent idiopathic scoliosis |
| *WRB* | 1 | Adolescent idiopathic scoliosis |
| *LCA5L* | 0 | Adolescent idiopathic scoliosis |
| *HSF2BP* | 0 | Pancreatic cancer |
| *RRP1B* | 1 | Allergic march (atopic dermatitis to allergic rhinitis) |
| *PDXK* | 1 | Allergic march (atopic dermatitis to asthma) |
| *CSTB* | 2 | Hemoglobin concentration |
| *AGPAT3* | 1 | Hemoglobin concentration |
| *No data avaiable about AP001057.1 in this dataset!* | NA | Hemoglobin concentration |
| *LINC00111* | 0 | Hemoglobin concentration |
| *RIPK4* | 2 | Hemoglobin concentration |
| *PRDM15* | 2 | Hemoglobin concentration |
| *C2CD2* | 0 | Blood protein levels |
| *UMODL1* | 1 | Blood protein levels |
| *ABCG1* | 1 | Blood protein levels |
| *TFF3* | 1 | Blood protein levels |
| *KRTAP10-4* | 0 | Neuroticism |
| *KRTAP10-13P* | 0 | Smoking status (ever vs never smokers) |
| *UBE2G2* | 1 | Smoking status (ever vs never smokers) |
| *PTTG1IP* | 1 | White matter microstructure (fractional anisotropy) |
| *ITGB2* | 2 | White matter microstructure (fractional anisotropy) |
| *SEC14L4* | 0 | White matter microstructure (fractional anisotropy) |
| *SEC14L6* | 1 | White matter microstructure (fractional anisotropy) |
| *GAL3ST1* | 1 | White matter microstructure (fractional anisotropy) |
| *TCN2* | 2 | White matter microstructure (fractional anisotropy) |
| *SLC35E4* | 1 | White matter microstructure (fractional anisotropy) |
| *ZBED4* | 1 | White matter microstructure (fractional anisotropy) |
| *ALG12* | 1 | PR interval |
| *PIM3* | 1 | PR interval |
| *IL17REL* | 0 | PR interval |
| *TTLL8* | 0 | PR interval |
| *MLC1* | 1 | Schizophrenia (MTAG) |
| *MOV10L1* | 1 | Schizophrenia (MTAG) |
| *TRABD2A* | 0 | Schizophrenia (MTAG) |
| *SELO* | 0 | Schizophrenia (MTAG) |
| *SHANK3* | 1 | Schizophrenia (MTAG) |
| *TUBGCP6* | 2 | Schizophrenia (MTAG) |
| *HDAC10* | 3 | Schizophrenia (MTAG) |
| *MAPK12* | 3 | Schizophrenia (MTAG) |
| *MAPK11* | 2 | Schizophrenia (MTAG) |
| *PLXNB2* | 1 | Schizophrenia (MTAG) |
| *DENND6B* | 0 | Schizophrenia (MTAG) |
| *PPP6R2* | 1 | Schizophrenia (MTAG) |
| *VCX3A* | 0 | Schizophrenia (MTAG) |
| *PFKFB1* | 1 | Schizophrenia (MTAG) |
| *ALAS2* | 0 | Eosinophil percentage of white cells |
| *PAGE2* | 1 | Eosinophil percentage of white cells |
| *No data avaiable about RP11-622K12.1 in this dataset!* | NA | Eosinophil percentage of white cells |
| *USP51* | 1 | Eosinophil percentage of white cells |
| *RRAGB* | 2 | Eosinophil percentage of white cells |
| *KLF8P1* | 0 | Eosinophil percentage of white cells |
| *No data avaiable about RP11-445O16.3 in this dataset!* | NA | Hemoglobin concentration |
| *SPIN3* | 0 | Hemoglobin concentration |
| *SPIN2A* | 0 | Hemoglobin concentration |
| *FAAH2* | 0 | Hemoglobin concentration |
| *ZXDA* | 3 | Hemoglobin concentration |
| *KRT8P17* | 0 | Hemoglobin concentration |
| *No data avaiable about RP11-3D23.1 in this dataset!* | NA | Alcohol consumption (drinks per week) (MTAG) |
| *ARHGEF9* | 1 | Alcohol consumption (drinks per week) (MTAG) |
| *ZC4H2* | 1 | Alcohol consumption (drinks per week) (MTAG) |
| *No data avaiable about AL645819.1 in this dataset!* | NA | Alcohol consumption (drinks per week) (MTAG) |
| *DCAF12L2* | 1 | Alcohol consumption (drinks per week) (MTAG) |
| *DCAF12L1* | 0 | Alcohol consumption (drinks per week) (MTAG) |
| *CHMP3* | 1 | Alcohol consumption (drinks per week) (MTAG) |
| *DACH2* | 0 | Alcohol consumption (drinks per week) (MTAG) |
| *KLHL4* | 1 | Alcohol consumption (drinks per week) (MTAG) |
| *NAP1L3* | 3 | Neuritic plaques (SNP x SNP interaction) |
| *FAM133A* | 0 | Neuritic plaques (SNP x SNP interaction) |
| *GLUD2* | 1 | Neuritic plaques (SNP x SNP interaction) |
| *No data avaiable about RP13-192B19.2 in this dataset!* | NA | Neuritic plaques (SNP x SNP interaction) |
| *SLITRK2* | 3 | Neuritic plaques (SNP x SNP interaction) |
| *MPP1* | 2 | Neuritic plaques (SNP x SNP interaction) |
| *KDM5D* | 1 | Neuritic plaques (SNP x SNP interaction) |
| *EIF1AY* | 1 | Diffuse plaques (SNP x SNP interaction) |
