# Supplementary material for: Prioritization of risk genes for Alzheimer’s disease: an analysis framework using spatial and temporal gene expression data in the human brain based on support vector machine
Source: Front Genet. 2023 Oct 6;14:1190863. doi: 10.3389/fgene.2023.1190863 (PMC10587557; doi:10.3389/fgene.2023.1190863)
Supplement: Supplementary file 7 [file Table5.DOCX]

**Supplementary Table S5.** The result of some known AD risk genes’ prioritization.

| Gene | Probability |
| --- | --- |
| *PICALM* | 0.783 |
| *APOE* | 0.727 |
| *BIN1* | 0.723 |
